# Countries' experiences scaling up national breastfeeding, protection, promotion and support programmes: Comparative case studies analysis

**DOI:** 10.1111/mcn.13358

**Published:** 2022-04-19

**Authors:** Sonia Hernández‐Cordero, Rafael Pérez‐Escamilla, Paul Zambrano, Isabelle Michaud‐Létourneau, Vania Lara‐Mejía, Bianca Franco‐Lares

**Affiliations:** ^1^ Instituto de Investigaciones para el Desarrollo con Equidad (EQUIDE) Universidad Iberoamericana México City Mexico; ^2^ Department of Social and Behavioral Sciences Yale School of Public Health New Haven Connecticut USA; ^3^ Alive & Thrive Southeast Asia/FHI 360 Manila Philippines; ^4^ Department of Social and Preventive Medicine, School of Public Health Université de Montréal Montreal Quebec Canada

**Keywords:** breastfeeding, breastfeeding promotion, breastfeeding support, breast‐milk substitutes, policy, policy making

## Abstract

Scaling up effective interventions, policies and programmes can improve breastfeeding (BF) outcomes. Furthermore, considerable interest exists in learning from relatively recent successful efforts that can inform further scaling up, with appropriate adaptations, across countries. The purpose of this four‐country case studies analysis was to examine why and how improvements in BF practices occurred across four contrasting countries; Burkina Faso, the Philippines, Mexico and the United States of America. Literature reviews and key informant interviews were conducted to document BF trends over time, in addition to why and how BF protection, promotion and support policies and programmes were implemented at a national level. A qualitative thematic analysis was conducted. The ‘Breastfeeding Gear Model’ and RE‐AIM (Reach; Effectiveness; Adoption; Implementation; and Maintenance) frameworks were used to understand and map the factors facilitating or hindering the scale up of the national programmes and corresponding improvements in BF practices. Each of the studied countries had different processes and timing to implement and scale up programmes to promote, protect and support breastfeeding. However, in all four countries, evidence‐based advocacy, multisectoral political will, financing, research and evaluation, and coordination were key to fostering an enabling environment for BF. Furthermore, in all countries, lack of adequate maternity protection and the aggressive marketing of the breast‐milk substitutes industry remains a strong source of negative feedback loops that are undermining investments in BF programmes. Country‐specific best practices included innovative legislative measures (Philippines), monitoring and evaluation systems (United States of America), engagement of civil society (Mexico) and behavior change communication BF promotion (Burkina Faso) initiatives. There is an urgent need to improve maternity protection and to strongly enforce the WHO Code of Marketing of Breast‐Milk Substitutes.

## Key messages

- The effective scale up of national breastfeeding (BF) policies and programs has been identified as a global health priority.
- There is a need to systematically conduct and compare case studies to understand drivers for the successful dissemination and scale up of national breastfeeding policies and programmes.
- Implementation science frameworks were key to systematically identify key drivers for the scale up of BF protection, promotion, and support policies and programmes in Burkina Faso, Mexico, the Philippines and the United States.
- Advocacy, multisectoral political will, financing, monitoring and evaluation, and coordination were key for enabling the BF environments.
- In all countries, there was a strong need to improve maternity protection and to tighten regulation and enforcement of the WHO Code of Marketing of Breast‐Milk Substitutes.

## Graphical abstract

The purpose of these four‐country comparative case studies was to examine why and how improvements in breastfeeding (BF) practices occurred across four contrasting countries: Burkina Faso, the Philippines, Mexico and the United States of America. Advocacy, political will, evaluation, multisectoral coordination, monitoring and funding are key to the successful scaling up and impact of national BF programmes. There is an urgent need to improve maternity protection and regulation of the marketing of breast‐milk substitutes.

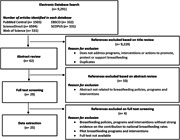

## 1 INTRODUCTION

The contribution of breastfeeding (BF) to child survival, health and development, as well as maternal health and national development, has been well documented (Victora et al., 2016). Protecting, promoting and supporting BF is highly cost‐effective (United Nations Children's Fund [UNICEF] & World Health Organization [WHO], 2017; Walters et al., 2019). Hence, scaling up national BF policies and programmes effectively has been identified as a global health priority (Pérez‐Escamilla & Hall Moran, 2016). Unfortunately, only 48.6% of infants younger than 6 months are exclusively breastfed in low‐ and middle‐income countries (Neves et al., 2021).

There is evidence that optimal BF behaviors, including exclusive BF (EBF), can rapidly improve when countries implement effective programmes (Pérez‐Escamilla et al., 2012). The scaling up of BF programmes could prevent an estimated 823,000 child deaths and 20,000 breast cancer deaths every year (Rollins et al., 2016). Also, it could reduce morbidity (Sankar et al., 2015; Victora et al., 2016) and improve children's educational potential and, probably, their income as adults (Victora et al., 2015).

The 2019 edition of The *State of the World's Children* reported an average global increase in EBF from 35% in 2005 to 42% by 2017, with some countries showing greater increases (United Nations Children's Fund (UNICEF), 2019). These findings have been recently confirmed (Neves et al., 2021). However, despite this increase, most countries are still far from reaching the 2025 EBF target of World Health Assembly Resolution 65.6, which aims to increase the rate of EBF in the first 6 months up to at least 50% (WHO, 2012). Therefore, there is an urgent need to better understand how successful scaling up efforts in countries has happened, in spite of having contrasting political, economic, social, cultural and health care systems contexts. Comparative case studies that are systematically conducted and analyzed are needed to generate this crucial evidence for informing the successful global dissemination, scale up, and maintenanc of effective national BF programmes. The objective of these case studies was to identify key facilitators and barriers for BF scale up at the national level across four countries with contrasting contexts, but where the rate of EBF improvement has been substantial during the past decade.

## 2 METHODS

We developed and followed a systematic methodology in the development of this comparative four‐country case study, as outlined in the following section.

### 2.1 Country selection

The inclusion criteria for country selection were (i) located in one of the following regions of the world: Asia, North America, Latin America or Sub‐Saharan Africa, and (ii) having positive EBF trends over the last decade. Among all countries that met the criteria mentioned above, those with the highest rate of change in EBF were identified (Table 1). Subsequently, one country was selected, per region of the world covered, based on (iii) a close consensus among the first three authors; (iv) the willingness of key informants (KIs) to participate and (v) the availability of the following information: BF policy documents; evidence of exisiting programmes to promote, protect and support BF at the national or sub‐national level; peer‐reviewed publications describing EBF trends and BF programmes, or policies. As a result, the countries selected were Burkina Faso, the Philippines, Mexico and the United States of America.

**Table 1:** EBF change rate among children under 6 months, by country

Country	Survey/source	EBF rate for infants <6 months (year)		EBF change rate^a^
North America and Latin America				
Belize	Belize Multiple Indicator Cluster Survey (Statistical Institute of Belize & UNICEF Belize, 2012, 2017)	14.7% (2011)	33.2% (2015–2016)	4.6
United States of America	National Health and Nutrition Examination Survey‐NHANES (Center for Disease Control and Prevention (CDC), 2017)	26.4% (2013–2014)	34.7% (2015–2016)	4.2
Mexico	Encuesta Nacional de Salud y Nutrición‐ENSANUT (Gutiérrez et al., 2012; Shamah‐Levy et al., 2020)	14.4% (2012)	28.6% (2018–2019)	2.4
Peru	Encuesta Demográfica y de Salud Familiar‐ENDES (Instituto Nacional de Estadística e Informática, 2018, 2019)	64.2% (2017)	66.4% (2018)	2.2
Asia				
Thailand	Thailand Multiple Indicator Cluster Survey (National Statistical Office of Thailand & United Nations Children's Fund, 2013, 2016)	12.3% (2012–2013)	23% (2015–2016)	3.6
Bangladesh	Bangladesh Demographic and Health Survey (National Institute of Population Research and Training NIPORT, Mitra and Associates, & ICF International, 2016; National Institute of Population Research and Training NIPORT & ICF International, 2019)	55.3% (2014)	65% (2017–2018)	3.2
State of Palestine	Palestinian Multiple Indicator Cluster Survey (Palestinian Central Bureau of Statistics, 2013, 2015)	28.7% (2010)	38.1% (2014)	2.4
Philippines	Expanded National Nutrition Survey (Food and Nutrition Research Institute and Department of Science and Technology, 2013, 2019)	52.3% (2013)	56.4% (2018–2019)	0.8
Sub‐Saharan Africa				
Burkina Faso	Enquête Nutritionnelle Nationale (Ministère de la santé, 2012, 2020)	38.2% (2012)	64.3% (2020)	3.3
Eswatini	Swaziland Multiple Indicator Cluster Survey (Central Statistical Office and UNICEF, 2011, 2016)	43.8% (2010)	63.8% (2014)	5.0
Sierra Leone	Sierra Leone Demographic and Health Survey (Statistics Sierra Leone SSL and ICF International, 2014, 2020)	31.4% (2013)	54.1% (2019)	3.8

Abbreviation: EBF, exclusive breastfeeding.

^a^ The EBF change rate was calculated as the sum of the last two national EBF prevalence rates divided by the period in years.

### 2.2 Case studies development

The country's case studies were designed to present information regarding BF outcomes trends over time, and the implementation of policies, programmes or actions to promote, protect and support BF at the national or sub‐national level. We used a variety of approaches to access, document and synthesize the information, including: (1) a literature review to identify reports and peer‐reviewed articles describing the process of implementation, monitoring and evaluation of policies, programmes and actions; and (2) KI interviews as needed to confirm and/or gather new information. Barriers and facilitators were identified using qualitative thematic analysis and then mapped to the Breastfeeding Gear Model (BFGM) (Pérez‐Escamilla et al., 2012) and RE‐AIM frameworks. RE‐AIM assesses (1) Reach: number of people impacted; (2) Effectiveness: impact; (3) Adoption: inclusion in the policy process; (4) Implementation: fidelity, adaptations and costs; and (5) Maintenance: extent to which it becomes part of the organization's standard practices (Jilcott et al., 2007).

#### 2.2.1 Literature review

We conducted a literature review of the academic and grey literature addressing the diffusion, dissemination, uptake and sustainability of BF programmes, policies and interventions in the four countries included. English and Spanish documents published between January 2010 and March 2021 were included. The search was performed in five electronic databases, including EBSCO, PubMed Central, Web of Science, SCOPUS and ScienceDirect. The key terms used included: ‘((exclusive breastfeeding OR breastfeeding promotion OR breastfeeding programmes) AND (breastfeeding program OR breastfeeding protection OR nutrition policy) AND (Scale‐up OR Sustainability) AND (Mexico OR Burkina Faso OR Philippines OR US))’.

#### 2.2.2 Key Informants interviews

We selected the key informants (KIs) based on their extensive knowledge of the BF environment in each country. We conducted in‐depth semistructured interviews remotely (Appendix S1) with individuals representing several sectors (society, academia, government authorities and international organizations) working on the protection, promotion and support of BF in each of the countries. The interview guide was developed similarly to previous case studies implemented by our team for the 2017 Lancet Early Childhood Development Series (Richter et al., 2017). Before the interview, a consent form (Appendix S2) was emailed to all interviewees for their written approval. The interviews were conducted through the Zoom platform by two authors (V. L.‐M. and S. H.‐C.), during the months of June to August 2021. Authorization to the interview was requested. The study was approved by the International Review Board of Universidad Iberoamericana in Mexico City.

#### 2.2.3 Qualitative thematic analysis

The case studies were analyzed using thematic analysis and as stated before, mapped to two frameworks, the BFGM and RE‐AIM. The BFGM is a Health Care Complex Adaptive Systems framework that has been successfully tested in eight countries across five world regions to identify or strengthen policies needed to enable the BF environments (Pérez‐Escamilla et al., 2012). We used the RE‐AIM implementation framework to identify the drivers for improvements in BF practices in each country. The RE‐AIM framework has been successfully used by our team in other recent case studies analyses of evidence‐based obesity policies and programmes (Pérez‐Escamilla et al., 2021). The BFGM allowed for these findings to be integrated into a pragmatic dynamic systems model.

### 2.3 Data analysis

Data were extracted from the articles and reports identified through the literature review using a structured extraction form with the following information fields: (i) study design; (ii) population and intervention or program characteristics; (iii) key impact outcomes; and (iv) program or intervention effectiveness. To understand the policy and program implementation features in each country, B. F.‐L. and V. L.‐M. extracted data using RE‐AIM (Jilcott et al., 2007). The extraction was performed by country and type of intervention. KIs provided needed information that was not available from the literature review.

KI interviews were transcribed *verbatim* with an online program (*Happyscribe*). Coauthors V. L.‐M. and B. F.‐L. checked the transcripts for accuracy before importing them into *Deedose* (version 9.0.17). The codes were based on the BFGM components or ‘gears': (i) advocacy; (ii) political will; (iii) legislation; (iv) funding and resources; (v) training and programmes implementation; (vi) behavior change communication campaigns; (vii) research and evaluation; and (viii) coordination (Pérez‐Escamilla et al., 2012). Coding was carried out by V. L.‐M. and B. F.‐L. in *Dedoose* (version 9.0.17) using the codebook developed for this study (Appendix S3). Finally, the four‐country case studies were first analyzed by country based on the RE‐AIM framework and the BFGM and then were compared across countries to identify common key facilitators and barriers based on an iterative consensus approach coordinated by S. H.‐C. and R. P.‐E.

## 3 RESULTS

### 3.1 Literature review

**Figure 1:**
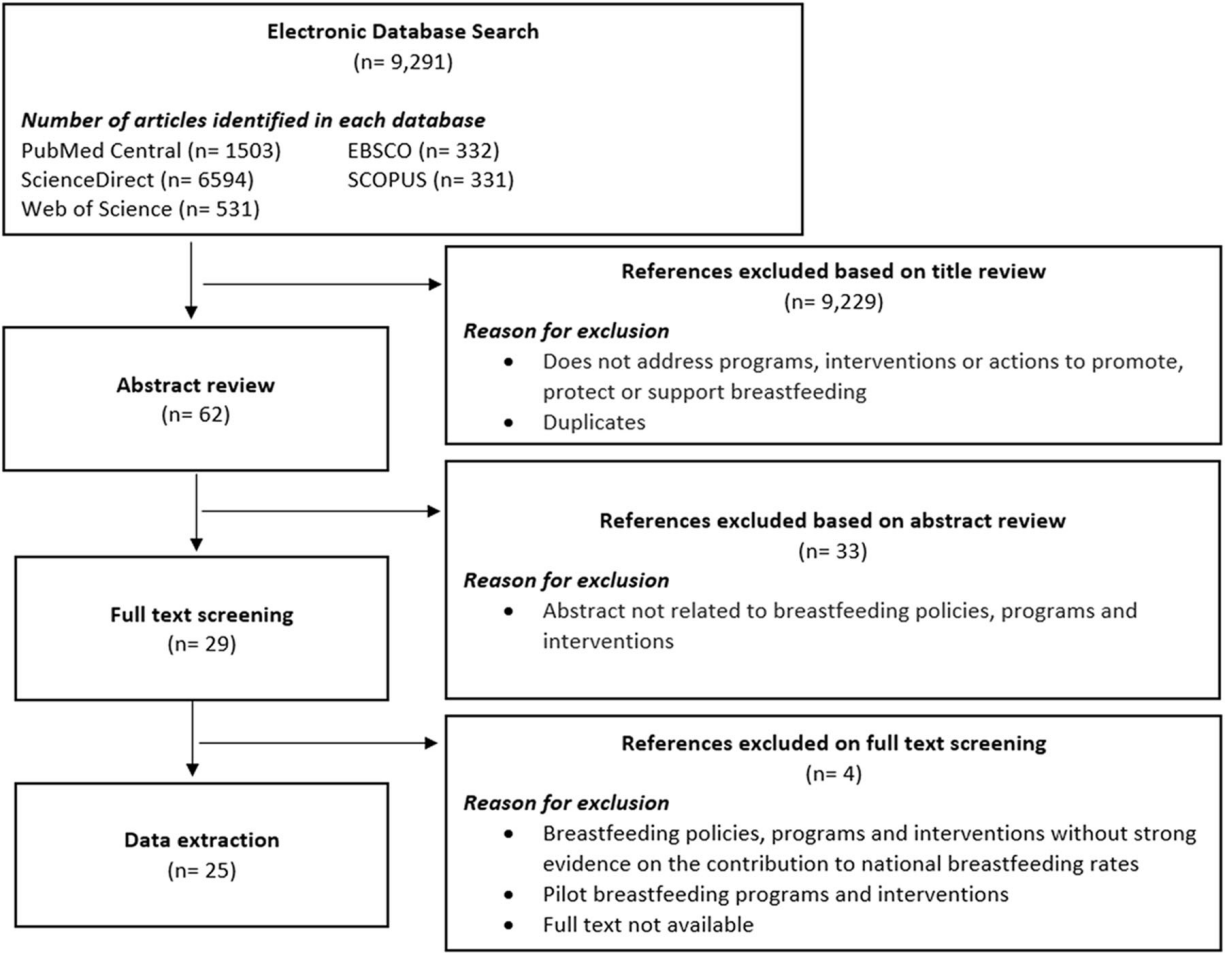
PRISMA diagram for literature review of policies, programmes, or interventions implemented in the countries selected. PRISMA, Preferred Reporting Items for Systematic Reviews and Meta‐Analyses.

The academic literature searches provided an initial sample of 62 unique articles published in peer‐reviewed journals. The titles and abstracts were screened for inclusion leading to 29 articles that were fully reviewed to determine eligibility, and 25 were included for data extraction (Figure 1). The main reasons for exclusion were lack of information on policies, programmes, or interventions to promote BF and unavailability of the full text. Regarding the grey literature, four documents and official web pages from international organizations were found. A total of 12 articles were not detected through electronic searches but were identified and shared by KIs. Therefore, we retained a total of 41 sources of information for final data extraction.

### 3.2 Key Informants interviews

In all countries, high‐level KIs were identified in the organizations or institutions selected a priori for this study. The detailed descriptive characteristics of the KIs' are included in Appendix S4. A total of 18 KIs were interviewed, with Government being the most represented sector (Table 2). The majority of the KIs were women (14 out of 18), with an average age of 48.6 years, and 4.7 years in their current employment. Interviews lasted 45 min, on average.

**Table 2:** Key informants by sector, four‐country breastfeeding case studies

Country	Interviews conducted	Academia	Civil society	Government	International organization
Burkina Faso	3	0	0	0	3
Mexico	5	2	1	1	1
Philippines	5	0	2	2	1
United States	5	0	2	3	0
Total	18	2	5	6	5

### 3.3 Four‐country case studies

The four‐country case studies are summarized below, highlighting similarities and differences between countries. The detailed findings of the study (Boxes 1, 2, 3, 4 and corresponding Supporting Information Materials) and illustrative quotes from the KIs (Table 3) are provided in the following sections.

Box 1 **Burkina Faso case study**

**Table:** 

Burkina Faso is a low‐income country in West Africa with a population of approximately 20,903,000 people (The World Bank, 2020a). According to The National Nutrition Survey, exclusive breastfeeding (EBF) increased by 26.1 percentage points: from 38.2% in 2012 to 64.3% in 2020 (Ministère de la santé, 2020). The Directorate of Nutrition (DN) has been central for the coordination of BF policies and programmes among the Ministry of Health itself and International and local nongovernmental organizations (NGOs). The PTF Nutrition (Technical and Financial Partners), created in 2012, has been a key channel for close coordination between the DN and PTF, which has allowed nutrition to become a priority in the country, helping to mobilize funding and define policy objectives.
In Burkina Faso, an initiative called 'Stronger with Breastmilk Only' (SBWO, 2021) and a program called GASPA (Groupes d'Apprentissage et de Suivi des Pratiques optimales d'Alimentation, for its acronym in French) has been key to foster an enabling environment for BF. SWBO is an initiative that includes an awareness campaign that promotes giving babies breast milk only, on‐demand and stopping the practice of giving water and other liquids and foods, from birth until the first 6 months of life. GASPA facilitates support groups for mothers and the target beneficiaries for this program are pregnant women, mothers with infants 0–6 months and mothers with infants 6–24 months. From the KIs' perspective, the above‐mentioned initiatives were probably successful as a result of a combination of several factors, such as the evidence collected earlier (formative research) and the identification of adaptations needed during implementation. The case of Burkina Faso highlights the importance of community mobilization across RE‐AIM (reach: number of people impacted, effectiveness: addresses the impact, adoption: prescription in the policy process, implementation: fidelity, adaptation and costs; and maintenance: addresses the extent to which it becomes part of the organization's standard practices) dimensions: reach, effectiveness and adoption, implementation and maintenance (Appendix S5).
'So based on this literature review, to identify the main determinant of not exclusively breastfeeding and these secondary determinant is that we support it, I mean, UNICEF and Alive and Thrive to work with the government to design a campaign called the Stronger Breast Milk Only and we are rolling out of this and for this campaign […] So we are adopting this socio‐ecological model, so we try to address everything at the country level, household level, community level and, of course, with the governmental policy level, we worked on that. And that's something very important to address the social norm around breastfeeding, exclusive breastfeeding, because, as you know, here in Burkina Faso breastfeeding is almost universal. But the main problem is exclusive breastfeeding' (International organization, Burkina Faso, [02B]).
Regarding legislation and policies, since 2004, pregnant women in Burkina Faso have been eligible for fully paid maternity leave of 14 weeks, which can be taken between 4 and 8 weeks before the expected date of childbirth. On the other hand, regarding the WHO Status report 2020 (World Health Organization [WHO], 2020a), Burkina Faso is moderately aligned with the International Code of Marketing of Breast‐milk Substitutes (the Code), which was recently updated in the country (March 2021) via the national decree no. 93‐279/PRES/SASF/MICM on the marketing and practices related to breast‐milk substitutes (BMS) products. The decree aligns with the World Health Assembly 69.9 resolution: 'Ending inappropriate promotion of foods for infants and young children' to regulate the marketing of BMS or any kind of milk specifically marketed for feeding infants and young children up to 3 years of age (WHO, 2016).
'…For the Code, we worked on that as well. We, there is a group of like 1993 there was a decree, here in Burkina Faso, to regulate how to, to, to, to market the marketing of the BMS. But unfortunately, it was not very effective because there were no sanctions and then it was out of date because there were few resolutions from WHO,… For space, and we worked with the technical secretary of nutrition and Food to [make] a better decree, we work on that, since 2016 and this year, in March, on March 17th, the Council of the Government adopted this newly updated decree. That is a big win for us…' (International organization, Burkina Faso, [02B]).
Over the last decade, infant and child feeding practices became a strong priority for the government (political will), which appears to be reflected in the support that the SWBO initiative has received from the previous and current Minister of Health who publicly expressed their commitment. Following the nutritional crisis in some countries of the Sahel region (Burkina Faso, Niger and Mali), many International NGOs came to provide support, and, as a result, nutrition activities were strengthened to prevent malnutrition. Prior to 2007, there was no specific nutrition directorate, so all the nutrition activities were under the Family Health Directorate. International Organizations' advocacy was essential for consolidating the creation of the DN, housed in the Ministry of Health, which currently coordinates all activities related to infant health and nutrition, including BF.
'…So, following that crisis, that nutrition crisis that occurred in 2005 and there was a shift, there were and a big change in the country because the nutrition activities were strengthened and the government had decided to put big importance in nutrition, international NGOs has come in the country and a lot of advocacy had started because at that moment there was not a direct specific directorate for nutrition… So, in 2007, the following, the advocacy and the fact that nutrition was being strengthened and consolidated in the country, there was a creation of the Nutrition Directorate…' (International Organization, Burkina Faso, [03B]).
The work of the DN appeared to be a catalyst for the implementation of different endeavors to support BF, such as the revision of maternity legislation, the development of mass communication strategies for behavioral change and the enactment of policies in favor of BF. The Burkina Faso government developed a National Infant and Young Child Feeding Scale Up Plan (2012–2025), with the partnership of Alive and Thrive (A&T), UNICEF, among other international agencies and NGOs (Scaling Up Nutrition, 2021). This is a multilevel strategic plan aimed at improving infant and young child feeding practices across sectors and at all levels, from the health system to the community, to standardize health and nutrition messages (Program delivery/Coordination). It includes training of traditional leaders, midwives and the creation of mother‐to‐mother support groups, through the GASPA described above.
'…The other point is that the country has tried to work at different levels, of community labor, health system, and to do mass advocacy. To have only one message at all levels of implementation. And this has been really a success, a success story, I can say, for example, the government or the Ministry of Health will work with journalists, with the midwives, with them, with the NGOs, with health workers they are implementing at all 13 regions. They are not working only at the central level, but in the 13 regions to make sure that there is the same information that is going on all over the country, including the traditional leaders. So this last year, the last five years, the country had really focused on traditional leaders because they have a very, how to say, a very a big commodity. I don't know how to say the word influence. I can say every influence of the population…' (International Organization, Burkina Faso [01B]).
Coordination also appears to be carried out effectively by different actors at several levels. In the case of the SWBO initiative, in 2020, a tripartite alliance composed of the DN, A&T and UNICEF, organized a national launch of the SWBO‐related campaign and subsequently supported subnational launches in the various regions. It helped to achieve combining different program components at both the community and health services levels to raise awareness of the importance of EBF (SBWO, 2021). Considering the SWBO campaign—a mass communication to foster behaviour change—the SWBO initiative also appears to involve the BFGM 'promotion' gear. It is worth mentioning that the support of international organizations has been key to sustainability through the financing of multiple actions.
'…local NGOs, most of them, they are doing promotion at the community level and that they are supporting the implementation of GASPA, this woman supports group and the work we funded community health workers. And again, that was the worry, the concern we have about the community works because it relates to much on external funding deals locally NGOs and its funding from donors…' (International Organization, Burkina Faso [02B]).
Burkina Faso has made substantial efforts to improve its EBF rate. However, some challenges or negative feedback loops have been identified. Although BF is a priority for the government, it currently lacks a specific government budget for BF, representing a risk to the sustainability of currently implemented actions to promote, protect and support BF. With respect to the Code, in spite of the current revision of the legislation, it has not yet fully incorporated all provisions related to it, and there is still contact between the BMS industry with health professionals for event sponsorship. Likewise, with regard to maternity leave, it is still not aligned with the International Labour Organization (ILO) Recommendation No. 191 (ILO, 2000), which encourages member countries to extend maternity leave to at least 18 weeks.
'There is the problem of financing, and this problem has an impact on the coverage of the community activities. But there is also another kind of problem to change the way of working. If every health district implements the community activities with the mother‐to‐mother support groups without waiting for a partner who is going to come and implement it with additional resources, this can help to scale up activities at the community without a lot of additional money…' (International Organization, Burkina Faso [03B]).
'… most of the health professionals are promoting breastfeeding. There is a small group that is still, how to say, that it's still corrupted. I can use this word? I don't know, by Nestlé, and that is still advising the utilization of breastmilk substitutes because Nestlé or and I don't know, another company, the most, the one that is working in the country is mainly Nestlé is, they are paying they're still paying some scholarships for pediatrician, or they are still paying some workshops organized by pediatricians. And so, they still have an influence on pediatricians, on the group of pediatricians. We are trying, UNICEF and the government […] to continue to advocate so that small groups continue to promote breastfeeding. I can say that, compared to the other countries where I had worked, in Burkina, is not the worst because most of them I can say 90 percent of pediatricians are promoting breastfeeding and the group that still had to change is not so big, but still the work has to continue' (International Organization, Burkina Faso [01B]).
'There is still a problem with the maternity leave, the maternity leave after the delivery, which is still at 14 weeks. So, there is still a place where there is still advocacy on that. OK, so the legislation part was, is trying to be more. The other factor of success in Burkina is that there are partners, very dedicated, for breastfeeding. Those partners are, for example, UNICEF Alive and Thrive, IBFAN' (International Organization, Burkina Faso [01B]).

Box 2 **Mexico case study**

**Table:** 

Mexico is an upper‐middle‐income country, in Latin America and the Caribbean region, with a population of 127,792,000 people in 2020 (OECD, 2021). Mexico has been doubling its exclusive breastfeeding (EBF) rate, from 14.5% in 2012 to 28.6% by 2018 (González‐Castell et al., 2020; World Health Organization (WHO), 2020b).
Between 2006 and 2018 the government invested in national policies and programmes to promote, protect and support BF; those identified through the literature review and mentioned by key informants were included in this analysis (Appendix S5). Although the current administration that came into power in 2018 has not continued with these efforts, Mexico was included because it has strong documentation of EBF improvements and implementation of large‐scale BF programmes during the timeframe of interest for our study. The Integrated Strategy for Attention to Nutrition (EsIAN, for its acronym in Spanish) was a pilot program within Prospera's conditional cash transfer program that aimed to strengthen the health and nutrition component by addressing the nutritional transition in Mexico and improving the health and nutrition of beneficiaries, with a strong focus on the first 1000 days (Hernández‐Licona et al., 2019). The three components of the EsIAN strategy were: (i) nutrition supplementation for pregnant and lactating women (tablets with micronutrients) and children aged 6–59 months (micronutrient powders, fortified porridge and milk), (ii) improved health care systems (specifically, equipment and quality of nutrition counseling) and (iii) behavior change communication and training, designed to promote infant feeding practices according to international recommendations. Moreover, the National Breastfeeding Strategy, implemented between 2014 and 2018, was a national policy that integrated different actions to promote, protect and support BF. Its specific aim was to increase the prevalence of girls and boys who are breastfed from birth and up to 2 years of age. It is noteworthy that the policy included strong intersectoral coordination including the health care system, the workplace and the community sectors. In addition, the strategy directly focused on the need to strengthen the dissemination and monitoring of compliance of the Code.
Regarding the legal status of the Code, Mexico is moderately aligned (WHO, 2020a), but has no defined and clear monitoring system nor sanctions. With regard to maternal employment, according to the International Labour Organization (ILO), female employees have been eligible for paid maternity leave of 12 weeks with full pay since 2009 (ILO, 2017). In the research area, the Becoming Breastfeeding Friendly (BBF) Toolbox, has been applied in Mexico three times since 2016 (Pérez‐Escamilla et al., 2022). Briefly, BBF is based on metrics to assess the strengths and weaknesses of the different components of the BFGM (Pérez‐Escamilla et al., 2012) to assess the country's readiness to scale up and making the corresponding evidence‐informed policy recommendations to enable the BF environment (González de Cosío et al., 2018). A result of the BBF multisectoral process since 2016 (Pérez‐Escamilla et al., 2012) is the strong advocacy for improving maternity leave benefits in Mexico for women employed in both the formal and informal economy (Vilar‐Compte et al., 2019).
Mexico's effort to improve infant feeding practices was initiated after the sharp decrease in BF practices shown by the National Health and Nutrition Survey in 2012 (Monitoring and evaluation) (Gutiérrez et al., 2012).
'…I believe that starting around the year two thousand fourteen, a process of visibility and awareness was initiated based on the data from the 2012 National Health and Nutrition Survey, which indicated a dramatic drop in breastfeeding practices in Mexico. I believe that the publication of this information and its appropriation by some scientific research institutes and civil society organizations as a fundamental element to publicly advocate in favor of breastfeeding detonated a series of movements that have, uh, advocated in favor of breastfeeding…' (Civil Society, Mexico [05M]).
'…One of the factors that I think that, uh, set off all the alarms was the Health and Nutrition Survey in 2012, where, well, it was reported that only 14.4% of the population was exclusively breastfeeding. So, I think that this evidence made everyone start to see, in one way or another, what activities could be done to promote, protect and support breastfeeding in different areas' (International Organization, Mexico [01M]).
This brought to the attention the urgent need to act in favor of improving BF practices in the country. International organizations, Civil Society, Academia and Government allies started to identify strategies and opportunities to improve BF practices, a process that was strongly advanced by the BBF initiative as it has allowed them to work in a coordinated manner.
'… It may have been the aggregate of small actions that academia and civil society have been pushing together with international organizations. But, well, they were like little ant steps…' (Academia, Mexico [04M]).
'… the work of research institutes and international organizations such as those I mentioned, have been key in progressively modifying breastfeeding practices in Mexico…' (Civil Society, Mexico, [05M]).
'… But also, at the level of civil society, academia and United Nations agencies. Especially among us, if we join efforts to promote this practice in different sectors, right…' (International Organization, Mexico [01M]).
In the context of the sharp decline in BF practices in the country and evidence‐based BF advocacy, BF made its way into the government's agenda. In 2014, the Secretary of Health in Mexico promoted the reform of the 'Ley General de Salud' (Health General Law), which stipulates the mandatory set of actions that contribute to increasing EBF and the duration of any type of BF including training of health professionals in the BF area. As a result of this amendment, the 2013–2018 National Development Plan included the need to promote BF through actions to increase the duration of BF, EBF through BF promotion programmes and BF training of health personnel. It is in this context that the National Breastfeeding Strategy (ENLM, for its acronym in Spanish) was launched by the Secretary of Health and was implemented from 2014 to 2018 as the national breastfeeding policy (Secretaría de Salud, 2013). The ENLM had important limitations, such as not having an assigned budget, the lack of clarity on the proposed indicators for its evaluation and the lack of an implementation and impact evaluation strategy based on a proper study design. Nevertheless, key informants considered that the Strategy did advance the cause of BF in Mexico because it included actions and guidelines for health services nationwide to engage in promoting, protecting and supporting BF.
'… Another important thing that happened during the last six years was the publication of the National Breastfeeding Strategy, which, although it was a strategy that did not have specific funding, was a strategy emanating from the National Breastfeeding Strategy. It was a strategy emanating from the Ministry of Health, with guidelines for all state health services, which established a line of actions to promote breastfeeding. I believe that this strategy, although it had some institutional weaknesses, did mark a milestone…' (Civil Society, Mexico [05M]).
'… The National Breastfeeding Strategy was implemented between 2014‐2018, and although it did not have a budget, well, we consider that it is an integrating axis of actions in favor of breastfeeding that was coordinated by the Ministry of Health in this period…' (Academia, Mexico [02M]).
'…There was a breastfeeding strategy, that is, a national promotion of breastfeeding, right? This one, which had some indicators, uh, and which, presented several people to talk about it… certainly put the topic into the agenda…' (Academia, Mexico [04M]).
In the same vein, various initiatives for amending laws and regulations emerged; for instance, the development of 'Norm project 050' ('Proyecto de norma 050') in 2018. Even though it has not yet been ratified, this norm is important because it sets out more specific criteria on actions that need to be taken for strengthening the promotion, protection and support of BF up to the age of 2 years, and with a strong focus on EBF during the first 6 months of life. This standard was proposed to be mandatory at the national level for health service personnel in the public, social and private sectors of the National Health System who are involved with maternal and child health care services, as well as for all persons, companies, or institutions that have contact with mothers of infants and young children. BF women and those involved in the care, feeding and development of children (Diario Oficial de la Federación [DOF], 2018).
'…General Health Council issued provisions to strengthen public policies related to breastfeeding and breastmilk substitutes and issued a decree reforming the General Health Law on human milk banks. There was another decree reforming some laws to protect, approve, promote, and support breastfeeding. There was a project of Norm 050…' (Academia, Mexico [02M]).
'…I believe that the government also began to analyze some regulations, which although they have not yet been published, such as NOM 050, there was finally a willingness, right?…' (International Organization, Mexico [01M]).
In Mexico, social mobilization, wellcoordinated work among key actors and evidence‐based advocacy represented a major step forward in the promotion, protection and support of BF. However, there are still major challenges to further improving BF practices in Mexico, especially due to the loss of political will as a result of the presidential transition (ie., the government that came to power in 2018 has abandoned many of the BF policies and programmes that likely contriuted to the doubling of EBF during the previous decade).
'…A breastfeeding strategy was renewed when the government changed and despite that, I know that entities such as Chihuahua specifically continued this one, implementing the breastfeeding strategy because that is what that entity decided, right? As they may also have their state strategies, but at the national level there is no longer any coordination, right? of a strategy that tries to unite the efforts of different areas at the federal level. There is also no longer, for example, the Interinstitutional Committee on Breastfeeding, which was led by the Gender Equity Commission. And although, I know that maybe they are going to take it up again, but once the government changed, all that effort was fragmented and it was not followed up adequately…' (Academia, Mexico [02M]).
'…I see a gigantic institutional fragility, right? That dragged from six years ago, right? As breastfeeding is left to the will of the administrator who arrives…' (Academia, Mexico [04M]).
'…Do you consider that in Mexico infant feeding practices, especially breastfeeding, are a priority in terms of developing policies, programs or interventions? No, absolutely not. I think absolutely not. In other words, I do not see any sector that is taking it as a priority in public policy…' (Academia, Mexico [04M]).
During the last decade, another important challenge identified was the lack of sufficient budget allocation to implement, monitor and evaluate strategies and initiatives to promote, protect and support BF, in accordance with the National Breastfeeding Strategy and other initiatives that were in place until recently.
'I think the main barrier is budgetary…This administration claims to be characterized by austerity, right? So administrative structures have been eliminated, programs have been eliminated and it is necessary to invest in breastfeeding, it is necessary to invest in training public servants, it is necessary to invest in expanding the baby‐friendly hospital program. It is necessary to invest in monitoring, it is necessary to invest in social communication actions favorable to breastfeeding. It is necessary to invest in supervising that companies do not violate the Code. It is necessary to invest in accompanying mothers who are pregnant and in the first months of breastfeeding to help them solve the thousands of doubts and problems they will have so that they do not abandon breastfeeding. And…when you talk about investment or budget, even though it is a highly profitable investment, all doors are closed, right? Because all the resources are destined to policy priorities that have nothing to do with breastfeeding…' (Civil Society, Mexico [05M]).
Finally, marketing of BMS is a common practice in Mexico, and very prominent via different communication channels including social media, in the context of a lack of enforcement of the WHO Code.
'An abominable presence of the industry, no? which starts before getting pregnant. I believe, right? To make us believe that it is part of this formula normalization process' (Academia, Mexico [04M]).
'…intra‐hospital practices that do not comply with the code and that also affect breastfeeding. Like still, there continues to be advertising or exposure of breastmilk substitutes in hospitals or immediate or once women leave the hospital. Doctors specifically are still one of the main actors, uh, recommending women to use infant formulas' (Academia, Mexico [01M]).
'Then there is also the exposure of advertising and marketing that now is also reaching us through, through digital means, through our phones, influencers, etcetera, that finally, well that, they make society believe that they don't have to know' (International Organization, Mexico [01M]).
'Another one has to do with all that is regulation,and changes to the regulatory framework to ensure compliance with the International Code of Marketing of Breastmilk Substitutes' (Academia, Mexico [02M]).

Box 3 **Philippines case study**

**Table:** 

The Philippines is a lower‐middle‐income country, located in the western Pacific Ocean, with a population of about 109,581,000 people in 2020, making it the twelfth most populated country in the world (The World Bank, 2020b). According to the 2019 Expanded National Nutrition Survey, exclusive breastfeeding (EBF) has an improvement from 52.3% in 2013 to 56.4% (Food and Nutrition Research Institute and Department of Science and Technology [FNRI‐DOST], 2019). Over the past decade, in the Philippines, BF protection, promotion and support have been included in many national multicomponent policies (e.g., a sign of political commitment) and national development strategies. Through the literature review and supplemented with the key informant interviews, we were able to identify the most representative policies and programmes that have enabled the BF environment in the country.
Based on formative research that included direct delivery observations in 2008, clinical practice guidelines for essential newborn care were developed through the GRADE approach. These guidelines were adopted in the Department of Health (DOH) Administrative Order 2009‐0025 with the objective of adopting new policies and the protocol 'Essential Intrapartum and Newborn Care' (EINC) (Sobel, Silvestre, et al., 2011). This protocol was key for institutionalizing a national package of cost‐effective time‐bound interventions, including immediate and thorough drying of the newborn; early and prolonged skin‐to‐skin contact (Moore et al., 2016); properly timed clamping and cutting of the cord after 1–3 min after birth, and nonseparation of the newborn from the mother for early BF initiation and rooming‐in. EINC became known as Early Essential Newborn Care (EENC) in the WHO Western Pacific Region in 2014 and is now commonly known as 'Unang Yakap' or The First Embrace in the Philippines. The implementation of this standard of care is ongoing in 8 additional countries (Cambodia, China, Lao PDR, Mongolia, Papua New Guinea, Philippines, Solomon Islands, Vanuatu, Viet Nam) in the region and is being regularly monitored.
'By 2010 and 2011, we had piloted the new practices, the sequence of drying skin to skin contact, delayed cord clamping and non‐separation, we call it “don't separate.” So, we call them the four core steps, the four core steps of immediate newborn care, number one: immediate and thorough drying, number two: skin to skin contact almost simultaneously, three: delayed cord clamping. And then four: non‐separation until completion of the first breastfeed. In a few years, we were able to scale up in the first pilot 11 hospitals…' (Civil Society, The Philippines [02P]).
'…The next law specifically states the rooming‐in policy regarding all mothers that would be giving birth should be, should have their babies room in with them within 24 hours. So, we also make sure that all children born areable to [receive] breast milk by their moms within 24 hours. We measure these children and for now, we can see also that their their performance is increasing and that itis very, very much beneficial for the government and the public and the community…' (Government, The Philippines [03P]).
The maintenance of the aforementioned policy has been the result of advocacy by different actors to achieve institutionalization generated through the political will and the resources needed for implementation and continuous monitoring. The DOH, with support from the WHO Country Office, commissioned Kalusugan ng Mag‐Ina, Inc. ('Health of Mother and Child'), a local nongovernmental organization (NGO) to conduct the evaluation of EENC every 2 years. Each country's results are compared with its own objectives or benchmarks and with the results of the other eight countries.
The Philippines' government enacted Executive Order 51 (EO51) or Philippine Milk Code in 1986 to regulate the marketing and distribution of breast‐milk substitutes (BMS) and established an Interagency Committee (composed of representatives from the DOH, Department of Trade and Industry, Department of Justice and the Department of Social Welfare and Development) to review advertising, promotional and marketing materials for products covered by the EO51. As a result, the country is substantially aligned with the Code (World Health Organization (WHO), 2020a, 2020b). The DOH is also implementing a monitoring system for reporting violations of the WHO Code, called Mother‐Baby Friendly Philippines. This monitoring was launched in 2017 and is conducted through an official database where families can report inappropriate marketing of BMS; however, actions against those who commit Code violations are still weak.
In the Philippines, advocacy and coordinated work and collaboration among different stakeholders have been key to strengthen the legislation. For instance, the 'Philippine Coalition of Advocates for Nutrition Security' (PHILCAN) helps to strengthen the Philippines' legislation. It is formed by 12 members from NGOs and its work is based on developing a clear and inclusive agenda that responds to the country's needs, based on evidence and supportive of national policies (Banerjee, 2017). They also invest in cultivating relationships with members, governments and all nutrition stakeholders to meet their objectives (Banerjee, 2017).
'…my NGO is a member of a coalition. It's called the Philippine Coalition of Advocates for Nutrition Security. And in the coalition, we have managed to really, really emphasize the compliance with the milk code […] So, basically what I'm saying is the role of international NGOs is also very, very aligned with the national channel trying to increase breastfeeding […] So, being working within a coalition with other NGOs, especially Save the Children, World Vision, World Food Program, having all the international NGOs really supporting and supporting breastfeeding is a really, really big help…' (Civil Society, The Philippines [02P]).
In the Philippines, there have been several revisions to legislation for protecting BF. The passage and enactment of the Republic Act No. 10028 (The Expanded Breastfeeding Promotion) in 2010, No. 11148 (The First 1000 Days Law) in 2018 and No. 11210 (105 Days Extended Maternity Leave Act) in 2019, was an important strengthening of the national legislation. Briefly, the Republic Act No. 10028, calls for BF breaks and designated facilities in the workplace. In addition, this act includes a provision that BF topics should be part of the curriculum of the education sector (training). On the other hand, Republic Act No. 11148 aims to scale up nutrition intervention programmes to improve the nutritional status and address the malnutrition of infants, adolescent females, pregnant and lactating women to ensure the growth and development of infants and young children. It is worth noting that this law aligns the definition of BMS (up to 36 months) with the recommended definition of World Health Assembly Resolution 69.9. The last one, Republic Act No. 11210 exhorts for extending paid maternity leave from 60–78 to 105 days with full pay, regardless of whether the delivery is vaginal or by cesarean section.
Finally, the increase in the DOH's budget for child health services, including BF, is another clear example that BF is a priority for the Philippines government resulting from the advocacy‐driven strong political will. This is also illustrated through the involvement in promotional activities, such as the creation of visual materials with the slogan 'Breastfeeding T. S. Ek' (Correct, Adequate and EBF).
'…Yes, yes, it's really a priority because I was telling you, it's part of the first 1000 days and even yeah, we know the benefit of it. So, it's really a priority when you talk about nutrition programs. Of course, this is one of the priorities that infant and young child nutrition, it is maternal infant and young child nutrition, if it's budgeted it […] But I know, it's really budgeted. It's really, it's really a priority. Definitely…' (International Organization, The Philippines [05P]).
'…So, from the very start, even when I was still with the government, the Department of Health has developed several materials on exclusive breastfeeding. And actually, we have this tagline or message or slogan that that was developed even, you know, years ago with which we are reviving and, you know, continuously disseminating and we call it breastfeeding check. The check is I mean, in Filipino, it's Tama because it's spelled, TSEK, which means Tama, Sapat at EKslusibo, which means Tama is right, breastfeeding is right, breastfeeding is sufficient and breastfeeding…should be exclusive…' (International Organization, The Philippines [05P]).
Despite the strong legislation and the current monitoring system in the Philippines, there is aggressive and pervasive marketing of the BMS industry. In addition, there is a major challenge in legislation regarding the involvement of the BMS industry in high‐level policy creating a conflict of interest; and the promotion of BMS on the internet and social media by influencers. One area of opportunity for the legislation is to strengthen advocacy in communities to counter aggressive BMS marketing. Similarly, an opportunity area of the official database for reporting violations of the Code could be to adopt the use of the platform so that it can be used by the entire population, regardless of their network access.
'…The politicians are using donations of food and milk, including milk to buy political leverage to buy, to buy popularity, etc. So, we're having to fight that. The expectation that the dole‐outs during the pandemic should include formula, should include cereals, Gerber Cerelac, these are not necessarily products, etc. […] So, yes, even politics, even the issue of nutrition is being used for political gain, especially here in the pandemic and social, it's all tied together, social, political […] There are more poor than rich in the Philippines. So, if they can buy off the poor by donating these products to families, then they'll be elected to public office next year. So, it's very, very difficult. Nutrition is becoming a political weapon…' (Civil society, The Philippines [02P]).
'Oh, well, I would like to say that there is really a weak monitoring, weak monitoring, and reporting of the violations and even the actions, you know, the actions on the report, that violation. I think that that is really still really weak. There is no in‐place monitoring platform. There is a platform that was developed by World Vision through the partnership with the Department of Health. But that platform for me, it's not it's not widely used because it's an app. You know, you have to download an app, blah, blah, blah. What if in the remotest barangay there is no Internet connection, so how they can report?' (International Organization, The Philippines [05P]).

Box 4 **United States case study**

**Table:** 

The United States is a country located in North America and it is among the world's largest countries by total area. It is made up of 50 states, one federal district, five large unincorporated territories, 326 Indian reservations and some smaller possessions, making it a country with a great variety of cultures and traditions (National Conference of State Legislatures [NCSL], 2018). BF rates in the United States have gradually increased in the past 20 years. Exclusive breastfeeding (EBF) has improved from 49.4% in 2011 to 58.3% in 2017 (Center for Disease (Center for Disease Control and Prevention [CDC], 2011 and Prevention National Immunization Survey [NIS], 2011; Center for Disease Control and Prevention (CDC), 2017). However, these estimates varied by race/ethnicity, mother's age and education, participation in WIC Programme (The Special Supplemental Nutrition Program for Women, Infants and Children), and family income. National estimates indicate substantial differences between non‐Hispanic Black and non‐Hispanic White infants across BF indicators in the United States. Three‐quarters (76%) of Black infants are ever breastfed, which is below the national average of 84.1% (2017) (CDC, 2020). Although the great majority of infants started BF, only 58.3% of infants were breastfed at 6 months (CDC, 2020). For this reason, the United States has been actively implementing programmes and policies aimed at the protection, promotion and support of BF.
'…I think like, you know, like women who immigrate from other countries, particularly among like Latin America, it's more, it's culturally ingrained to breastfeed. So, to them, it's carrying a practice where they have a stronger support system. Whereas in the US, just given the history of how, like black Americans ended up here, you know, there is just not the same support. There's not the same community perception regarding breastfeeding. So, I think that there's just a lot of social factors that essentially tie back to historical trauma in the history of how we've treated certain people, groups in the country that, you know, impact like that have resulted in the decrease of breastfeeding rates as well…' (Government, USA [04U]).
The CDC's Division of Nutrition, Physical Activity and Obesity has been strongly committed to increasing BF rates through promoting and supporting optimal BF practices over the past decades. The CDC plays an essential role in the research and monitoring of infant feeding practices in the United States. The 'Breastfeeding Report Card' provides data on BF practices and supports in the country every two years. Furthermore, BF data from the NIS continue to be released annually. CDC has also been monitoring maternity practices, which has helped identify areas of opportunity to strengthen BF‐related in‐hospital practices, staff skills and discharge support. This is done through a biannual national hospital survey called Maternity Practices in Infant Nutrition and Care. The CDC then provides specific feedback to hospitals so they can implement evidence‐based strategies to improve BF support. Additionally, CDC provides funding for interventions related to maternity care practices in the health sector to achieve the Baby‐Friendly designation and at the community level through REACH (Racial and Ethnic Approaches to Community Health) grants.
'[…] CDC does a lot of tracking with breastfeeding rates. We release annual breastfeeding rates. That's something that has been critical to tracking the progress over the last ten years and seeing where we have continued to make progress and where areas maybe weren't progressing as quickly as we would like them to. But we also look at maternity care practices and document how those maternity care practices are changing over time. And hospitals have quite honestly used that data to go back to their administrators and talk about what are some of the things that we can do in our hospitals to improve the practices that we have to help support breastfeeding women […]' (Government, USA [05U]).
The United States has institutionalized programmes that have contributed for several decades to improving nutritional status and tackling food insecurity among the most vulnerable populations. The gradual decline in BF rates in the United States beginning in the early 20th century prompted the United States Congress to enact legislation to address malnutrition among low‐income pregnant, lactating, or post‐partum women and their infants and children. In 1966 the Child Nutrition Act was enacted, which in Section 17 includes the WIC Legislative Requirements. The WIC was established as a permanent program in 1974 to safeguard low‐income pregnant, BF and non‐BF post‐partum women and children up to age five who are at nutritional risk (United States Department of Agriculture (USDA), 2013). It comprises four main components: nutrition education (promotion campaigns), BF support, healthy food packages and referrals to health and social services (USDA, 2013). The BF Peer Counselling Programme adds a critical dimension to WIC's efforts by providing a valuable service to their communities, addressing some of the most common BF barriers by offering BF education, support and role modeling (National WIC Association, 2019).
'… our breastfeeding peer counseling program is actually really good. It's been shown research shows that it's helped increase breastfeeding initiation rates within the program, particularly among black women who in the US historically have lower breastfeeding rates, they're less likely to breastfeed. So, I think that and due to the work of the National WIC Association, we've been able to get fully funded for the Breastfeeding Care Counseling Program. But I think ways in which we can expand that program within WIC will only further support breastfeeding mothers in the program. One of the best parts about that program is that breastfeeding peer counselors are women who usually have participated in WIC, they come from the same communities as the WIC participants, so they understand the culture, the language and so I think the great thing about that program, in particular, is that you are, you know, sometimes it can be intimidating when you're getting like just health care advice from a professional because it's like you may perceive them as being like above you. But with the breastfeeding peer counseling program, it's like you're talking to a peer, someone that's relatable, that understands you and knows how to support you, based on, like, your background…' (Government, USA [04U]).
Sustainability of programmes and initiatives as well as its monitoring are very strong in the United States, as evidenced by the Baby‐Friendly Hospital Initiative (BFHI). Baby‐Friendly USA, the national authority overseeing this programme, facilitates the health personnel training needed and provides technical assistance to hospitals in their pursuit of initial accreditation and subsequent reaccreditations. To achieve accreditation, hospitals must complete their '4D pathway' approach (Discovery, Development, Dissemination and Designation), which includes the staff training plan to integrate the 'Ten Steps to Successful Breastfeeding' into their maternity practices. Baby‐Friendly designation is conferred for a period of 5 years; the 3 years post designation is the Annual Quality Improvement Phase to assist facilities maintain compliance (sustainability), and the final 2 years is for Redesignation Phase.
'… Oh, yeah, let me just, I had ah, OK, let me just the pathway here, maybe. So, this is our process of accreditation, it's on our website. If you if you need, which I can share with you. But a new hospital will go through this 4‐D pathway. And typically, there is approximately a year in each of the phases. So, each of the phases in the 4‐D pathway usually take about a year. So, it takes about four years and then on‐site assessment and designation. So, the facilities are required to submit materials to us throughout the 4‐D pathway, and then prior to that designation, they submit a tremendous number of items to demonstrate their compliance with our guidelines and evaluation criteria. And so, when they become designated, which is typically four years, sometimes a little bit longer, then we redesignate every five years. And during that five‐year period after initial designation to redesignation, there are three years for annual quality improvement and two years in the redesignation process. So, during all five years, there is accountability. They do they are required to submit quality improvement and monitoring data every year that they are in that redesignation phase. And then they, you know, they are up for reassessment every five years…' (Civil society, USA [01U]).
BF progress in the United States has been in part the result of well‐coordinated collaborative work that includes key stakeholders in BF promotion and support. In May 2011, the Federal Interagency Breastfeeding workgroup was created with members who represent 16 individual agencies (Anstey et al., 2016). It includes the Department of Labor, Office of Women's Health, CDC, USDA, and National Institute of Health (NIH), among others. This workgroup provides a forum for information exchange across federal agencies and encourages collaborative approaches to address recommendations based on sound research conducted by the CDC and academia. The creation of this group helped to increase the capacity of the United States Breastfeeding Committee (USBC) and affiliated state coalitions. The USBC is an independent, nonprofit coalition of more than 100 influential professional, educational and governmental organizations, whose goal is to advance collaborative efforts to promote policies and practices that create an encouraging environment for BF across the country (advocacy). Unlike other countries, International Organizations are not visible in the United States, with the exception of the WHO with its participation through the BFHI.
'[…] we work across the agencies. So, there's even a federal interagency breastfeeding workgroup that has representatives across the board, everybody from the Department of Labor, Office of Women's Health, CDC, USDA, NIH. There's a whole range of groups that are involved and helping coordinate and collaborate so, we can talk within the federal government about the things that we're doing. Civil organizations like USBC or an organization like Thousand days tend to be doing more of the advocacy work. […] I haven't talked as much about academia, but they are a player in this as well and I think one of the things that they do very well is research […] We see less in the US with interactions with international organizations. That's not to say that they're not important. I think the World Health Organization is a huge, huge piece here and especially with the baby‐friendly hospital initiative. So, they are a part of it. They just aren't as much of a part as maybe other countries. So, we haven't seen their role or maybe I haven't seen their role as much as some of the other organizations' (Government, United States [05]).
Although the United States has a strong program delivery and research and evaluation gear, some challenges were identified to improve the BF environment. Between 2016 and 2020 there were budget allocation for interventions that promote, protect and support BF due to major changes in government. Furthermore, the new political administration elected in 2020 is now giving evenmore priority and attention to public health issues, including BF.
The political support (political will) for BF in the country was recently crystalized in the 2020 Dietary Guidelines for Americans, the policy document upon which all federal food and nutrition policy is based, for the first time included recommendations for children under two that strongly endorsed BF. By contrast, BF protection in the United States is weak because for instance none of the WHO Code provisions has been ever adopted in the United States; therefore, the unchecked advertising of BMS is widespread and increasing in the country. Another major challenge is the lack of national laws for BF protection. While all 50 states, the District of Columbia, Puerto Rico and the Virgin Islands have laws that specifically allow women to breastfeed in any public or private locations, only 30 states, the District of Columbia and Puerto Rico have laws related to BF in the workplace. The Family and Medical Leave Act provides 12 weeks of unpaid leave to workers in companies with less than or equal to 50 employees. Currently, eight states (California, New Jersey, Rhode Island, New York, Washington state, Massachusetts, Connecticut and Oregon) and Washington DC have enacted laws offering paid family leave; however, it is partially paid for between 6 and 12 weeks, which is way less than the International Labour Organization (ILO) recommendation (ILO, 2000; NCSL, 2020).
'[…] there are two major ones (challenges). The first one is the marketing of breastmilk substitutes by formula companies. They are very aggressive and somewhat insidious in their efforts to undermine breastfeeding. The international code of marketing of breastmilk substitutes was never signed by the United States and was never agreed to by the United States. And in the United States, there is a big issue with marketing by formula companies, and then I would say the second is really the lack of paid family leave. Many women return to work early after giving birth out of necessity or certainly fear of losing their jobs. So, the institution of paid family leave, a national paid family leave program would be, I think, would enhance breastfeeding in the United States. […]' (Civil Society, USA [01U]).
'…So overall, yes, I think the US government and the United States are putting breastfeeding as a priority. At the federal level, one of the things that I think is really exciting is the most recent US dietary guidelines. The 2020 to 2025 included children birth to twenty‐four months of age for the first time. And as part of that, they talked about exclusive breastfeeding for about the first six months. This is huge. This is the first time that we have had a comprehensive dietary guidance for children from birth to twenty‐four months. It's also the first time that we had federal guidance on exclusive breastfeeding. All our other recommendations have come from the American Academy of Pediatrics. So, for this to come from a federal document that now all federal programmes need to adhere to is a huge step forward…' (Government, USA [05U]).
In regard to the United States, it is worth highlighting that the collaborative work through the Federal Interagency group has indeed impacted all RE‐AIM dimensions.

**Table 3:** Representative quotes from in‐depth interviews based on the Breatsfeeding Gear Model (BFGM)^a^

Node (BFGM gear)	Quotations
Advocacy	‘… that nutrition crisis that occurred in 2005 and there was a shift, there were and a big change in the country because the nutrition activities were strengthened and the government had decided to put a big importance in nutrition, international NGOs has come in the country and a lot of advocacy had started because at that moment there was not a direct specific directorate for nutrition at the Ministry of Health. Nutrition was handled by the Directorate of Family Health. So, in 2007, the following, the advocacy and the fact that nutrition was being strengthened and consolidated in the country, there was a creation of the Nutrition Directorate, which was there for nutrition activities. And this had a big shift also for to increase nutrition activities…’ (International organization, Burkina Faso, [03B]).
	‘…And then I think in your question, you had also asked about international organizations. We see less in the US with interactions with international organizations. That's not to say that they're not important. I think the World Health Organization is a huge, huge piece here and especially with the baby friendly hospital initiative. So, they are a part of it. They just aren't as much of a part as maybe other countries. So, they have a, we haven't seen their role or maybe I haven't seen their role as much as some of the other organizations…’ (Government, USA, [05U]).
	‘… International organizations are always advocating together with public agencies, such as UNICEF and PAHO, who have always been looking for the implementation of initiatives and legal changes for the protection of breastfeeding together, for example, with the National Institute of Public Health, which we have also been working with them hand in hand on that. The Universidad Iberoamericana. In this new government we have identified, for example, SIPINA, which is the “System for the Integral Protection of Children and Adolescents” that within the National Strategy for Early Childhood had incorporated the part of promoting breastfeeding and protection of the right to breastfeed…’ (Academy, Mexico, [02M]).
Political will	‘…So, overall, yes, I think the US government and in the United States are putting breastfeeding as a priority. At the federal level, one of the things that I think is really exciting is the most recent US dietary guidelines. The 2020 to 2025 included children birth to twenty‐four months of age for the first time. And as part of that they talked about exclusive breastfeeding for about the first six months. This is huge. This is the first time that we have had comprehensive dietary guidance for children birth to twenty‐four months. It's also the first time that we had federal guidance on exclusive breastfeeding. All our other recommendations have come from the American Academy of Pediatrics. So, for this to come from a federal document that now all federal programs need to adhere to is a huge step forward. And I think we're going to continue to see the ramifications of that particular document for years to come…’ (Government, USA, [05U]).
	‘… but it is also true that in terms of regulation, there is a specific interest on the part of Cofepris (Committee for Protection from Sanitary Risks) to make some modifications to the regulations, to the advertising guidelines, right? As I mentioned, right? The Code, the compliance with the Code, the monitoring of the Code, if there is, if they have expressed it, we have it in writing. However, maybe the events, the progress is a little bit slower than I would like to see, but maybe because that is the way it is and we have to be patient…’ (International Organization, Mexico, [01M]).
Legislation and policies	‘…I told you early on that the Republic Act 11148, which is the First One Thousand Days Law, that's one. We have the very good Milk Code and the revised implementing rules and regulations. We also have the infant and young child feeding Strategic Plan which is being updated and we have now the updated version and we called it the IYCF 2020‐2030 Strategic Plan. What else do we have? We also have passed, expanded, expanded maternity leave, our maternity base leave. Yeah. So that is one, I think that, you know, as one of the drivers on why at least we have increased, you know, the exclusive breastfeeding rates in the Philippines based on the latest survey…’ (International Organization, The Philippines, [05P]).
	‘… Until 2018, the National Strategy for Breastfeeding of Breastfeeding of the Ministry of Health was in force, which had a perspective […] let's say mandatory for the health sector, but also of reference for other instances of government and for the social and private sector. I think this allowed the reinforcement of various training and dissemination activities. Now these strategies are part of the Specific Action Program on Sexual and Reproductive Health, 2020‐2024, where there is a priority objective that address perinatal health. And within this objective there are several actions related to the issue of breastfeeding in regard to health personnel, right?…’ (Academy, Mexico, [03M]).
Funding and resources	‘…I think the main barrier is budgetary…This administration claims to be characterized by austerity, right? So administrative structures have been eliminated, programs have been eliminated and it is necessary to invest in breastfeeding, it is necessary to invest in training public servants, it is necessary to invest in expanding the baby‐friendly hospital program. It is necessary to invest in monitoring, let us say, private hospitals so that they do not fail to comply with the provisions of the Code; it is necessary to invest in social communication actions favorable to breastfeeding. It is necessary to invest in supervising that companies do not violate the marketing code. It is necessary to invest in accompanying mothers who are pregnant and in the first months of breastfeeding to help them solve the thousands of doubts and problems they will have so that they do not abandon breastfeeding. And there is one, that is, when you talk about investment or budget, even though it is a highly profitable investment, all doors are closed, right? Because all the resources are destined to policy priorities that have nothing to do with breastfeeding…’ (Civil Society, Mexico, [05M]).
	‘…So, there has been a consistent budget line for CDC to help promote and support breastfeeding in hospitals, and that has been an important piece for CDC to be able to continue to support the work for hospital level programs for state and community level programs…’ (Government, USA, [05U]).
Training and program delivery	'…Yes, actually, we have the RA 7600 and RA 10028, which is an expanded breastfeeding act, we have already included this provision that it should be part of the curriculum of our education sector. So breastfeeding and the other important things about breastfeeding are part of the curriculum from the grade school or the younger kids to the high school level to the college level. So, it's already been integrated. We have already partnered with the Ministry of Education, also the Ministry for Higher Education, and also it has been integrated in the curriculum of those in the special education like technical and education skills, which is not a four‐year course, but only 2 year courses. It has also been included in those curricula…‘ (Government, The Philippines, [03P]).
	'… and the other thing is that there are NGOs that are implementing the strategic plan of action on infant and young child feeding, mainly at community level. They are working in the area of community with the mother to mother support groups. And the specificity of Burkina Faso is that there are three groups of mothers, mother's support groups. There is a group of pregnant women, a group of mothers that have children under six years, six months, six excuse me, and another group with the mother, lactating mothers with the children between six to 23 months. Ok? So what is good on this? Is that the content of the message is adapted to the specific group…’ (International organization, Burkina Faso, [01B]).
Promotion	‘…So. Last year, we had, we started a campaign, a national campaign or initiative on stronger with breast milk only. It is a campaign, and we make an alliance with Alive and Thrive and UNICEF to support the government and later on, we had the support, a big support from the World Bank, a project, a bilateral project supporting the Ministry of Health. They put a lot of funding. We start these UNICEF and Alive and thrive and finally, when the support the government to develop a strategy and a plan of action budget and finally, what World bank came in and had funded that that big campaign. So, this is the big campaign that started last year that helped us to have actually to reach to have a good achievement…’ (International organization, Burkina Faso, [01B]).
Research and evaluation	‘…CDC does a lot of tracking with breastfeeding rates. We release annual breastfeeding rates. That's something that has been critical to tracking the progress over the last ten years and seeing where we have continued to make progress and where areas maybe weren't progressing as quickly as we would like them to. But we also look at maternity care practices and document how those maternity care practices are changing over time. And hospitals have quite honestly used that data to go back to their administrators and talk about what are some of the things that we can do in our hospitals to improve the practices that we have to help support breastfeeding women…’ (Government, USA, [05U]).
Coordination, goals and monitoring	‘…I feel that's another impetus, another factor that has helped our breastfeeding rates go up. Our Milk Code monitoring. We are trying our best to improve the monitoring of Milk Code violations. In fact, there was a project for a portal where mothers, anybody can report Milk Code violations. The portal worked fairly well. There were many, many reports…’ (Civil Society, The Philippines, [02P]).
	‘…The other point is that the country has tried to work at different levels, a community labor, health system, and to do mass advocacy. To, to have only one message at all levels of implementation. And this has been really a success, a success story, I can say, for example, the government or the Ministry of Health will work with journalists, with the midwives, with them, with the NGOs, with the work health workers they are implementing at all 13 regions. They are not working only at the central level, but in the 13 regions to make sure that there is the same information that is going on all over the country, including the traditional leaders. So, this last year, the last five years, their country had really focused on traditional leaders because they have a very, how to say, a very big commodity. I don't know how to say the word influence. I can say every influence of the population…’ (International organization, Burkina Faso, [01B]).
	‘…When we did our baseline study among the 481 deliveries in 51 hospitals, the skin to skin contact rate in that research‐based study was only 8%. It was like maybe only one or two mothers who got skin to skin. But by the time we inserted skin to skin contact as a population survey indicator, it was up. I think till maybe 64% just by just by teaching health workers to, we like to say we rechoreographed what the health workers do after delivery instead of routine suction and immediate and immediate cord clamping…’ (Civil Society, The Philippines, [02P]).

Abbreviation: BFGM, Breastfeeding Gear Model.

^a^ Quotes from Mexico were translated from Spanish to English as expressed by the participants.

#### 3.3.1 Case study #1: Burkina Faso

Burkina Faso is a low‐income country in West Africa with a population of approximately 20,903,000 people (The World Bank, 2020a). According to The National Nutrition Survey, EBF increased by 26.1 percentage points: from 38.2% in 2012 to 64.3% in 2020 (Ministère de la santé, 2020). The Directorate of Nutrition (DN) has been central for the coordination of BF policies and programmes among the Ministry of Health itself and International and local nongovernmental organizations (NGOs). The PTF Nutrition (Technical and Financial Partners), created in 2012, has been a key channel for close coordination between the DN and PTF, which has allowed nutrition to become a priority in the country, helping to mobilize funding and define policy objectives (Box 1).

In Burkina Faso, an initiative called ‘Stronger with Breast milk Only’ (SWBO) (SBWO, 2021) and a programme called GASPA (Groupes d'Apprentissage et de Suivi des Pratiques optimales d'Alimentation, for its acronym in French) have been key to foster an enabling environment for BF. SWBO is an initiative that includes an awareness campaign that promotes giving babies breast milk only, on‐demand and stopping the practice of giving water and other liquids and foods, from birth until the first 6 months of life. GASPA facilitates support groups for mothers and the target beneficiaries for this program are pregnant women, mothers with infants 0–6 months and mothers with infants 6–24 months. From the KIs' perspective, the above‐mentioned initiatives were successful as a result of a combination of several factors, such as the evidence collected earlier (formative research) and the identification of adaptations needed during implementation. The case of Burkina Faso highlights the importance of community mobilization across RE‐AIM dimensions: reach, effectiveness and adoption, implementation and maintenance (Appendix S5). Regarding legislation and policies, since 2004, pregnant women in Burkina Faso have been eligible for fully paid maternity leave for 14 weeks, which can be taken between 4 and 8 weeks before the expected date of childbirth. On the other hand, according to WHO (WHO, 2020a), Burkina Faso is moderately aligned with the International Code of Marketing of Breast‐milk Substitutes (the Code), which was recently updated in the country (March 2021) via the national decree no. 93‐279/PRES/SASF/MICM on the marketing and practices related to breast‐milk substitutes (BMS) products. The decree aligns with the World Health Assembly 69.9 resolution: ‘Ending inappropriate promotion of foods for infants and young children’ to regulate the marketing of BMS or any kind of milk specifically marketed for feeding infants and young children up to 3 years of age (WHO, 2016). Over the last decade, infant and child feeding practices became a strong priority for the government (political will), which appears to be reflected in the support that the SWBO initiative has received from the previous and current Minister of Health who publicly expressed their commitment. Following the nutritional crisis in some countries of the Sahel region (Burkina Faso, Niger and Mali), many International NGOs came to provide support and, as a result, nutrition activities were strengthened to prevent malnutrition. Before 2007, there was no specific nutrition directorate, so all the nutrition activities were under the Family Health Directorate. International Organizations' advocacy was essential for consolidating the creation of the DN, housed in the Ministry of Health, which currently coordinates all activities related to infant health and nutrition, including BF.

The work of the DN appeared to be a catalyst for the implementation of different endeavours to support BF, such as the revision of maternity legislation, the development of mass communication strategies for behavioral change and the enactment of policies in favor of BF. The Burkina Faso government developed a National Infant and Young Child Feeding Scale Up Plan (2012–2025), in partnership with Alive and Thrive (A&T), UNICEF, among other international agencies and NGOs (Scaling Up Nutrition, 2021). This is a multilevel strategic plan aimed at improving infant and young child feeding practices across sectors and at all levels, from the health system to the community, to standardize health and nutrition messages (Programme delivery/Coordination). It includes training of traditional leaders and midwives and the creation of mother‐to‐mother support groups, through the GASPA described above.

Coordination also appears to have been carried out effectively by different actors at several levels. In the case of the SWBO initiative, in 2020, a tripartite alliance composed of the DN, A&T and UNICEF, organized a national launch of the SWBO‐related campaign and subsequently supported subnational launches in the various regions. It helped to achieve combining different program components at both the community and health services levels to raise awareness of the importance of EBF (SBWO, 2021). Considering the SWBO campaign—a mass campaign to foster behaviour change—the SWBO initiative fits well within the BFGM ‘promotion’ gear. It is worth mentioning that the support of international organizations has been key to sustainability through the financing of multiple actions.

Burkina Faso has been making substantial efforts to improve its EBF rate. However, some challenges or negative feedback loops were identified. Although BF is a priority for the government, it currently lacks a specific government budget for BF, representing a risk to the sustainability of currently implemented actions to protect, promote and support BF. With respect to the Code, in spite of the current revision of the legislation, it has not yet fully incorporated all provisions related to it, and there is still contact between the BMS industry with health professionals for event sponsorship. Likewise, with regard to maternity leave, it is still not aligned with the International Labour Organization (ILO) Recommendation No. 191 (ILO, 2000), which encourages member countries to extend maternity leave to at least 18 weeks (Box 1).

#### 3.3.2 Case study #2: Mexico

Mexico is an upper‐middle‐income country, in Latin America and the Caribbean region, with a population of 127,792,000 people in 2020 (OECD, 2021). Over the past decade, Mexico has doubled its EBF rate, from 14.5% in 2012 to 28.6% by 2018 (González‐Castell, 2020; WHO, 2020b).

Between 2006 and 2018 the government invested in national policies and programmes to promote, protect and support BF; those identified through the literature review and mentioned by KIs were included in this analysis (Appendix S5). Although the current administration that came into power in 2018 has not continued with these efforts, Mexico was included because it has strong documentation of EBF improvements and implementation of large‐scale BF programmes during the timeframe of interest for our study. The Integrated Strategy for Attention to Nutrition (EsIAN, for its acronym in Spanish) was a pilot program within Prospera's conditional cash transfer program that aimed to strengthen health and nutrition by addressing the nutritional transition in Mexico and improving the health and nutrition of beneficiaries, with a strong focus on the first 1000 days (Hernández‐Licona et al., 2019). The three components of the EsIAN strategy were: (i) nutrition supplementation for pregnant and lactating women (tablets with micronutrients) and children aged 6–59 months (micronutrient powders, fortified porridge and milk), (ii) improved health care systems (specifically, equipment and quality of nutrition counseling) and (iii) behavior change communication and training, designed to promote infant feeding practices according to international recommendations. Moreover, the National Breastfeeding Strategy, implemented between 2014 and 2018, was a national policy that integrated different actions to promote, protect and support BF. Its specific aim was to increase the prevalence of girls and boys who are breastfed from birth through 2 years of age. It is noteworthy that the policy included strong intersectoral coordination including the health care system, the labor sector and communities. In addition, the strategy directly focused on the need to strengthen the dissemination and monitoring of compliance of the Code.

Regarding the legal status of the Code, Mexico is moderately aligned (WHO, 2020a), but does not have a well defined and clear monitoring system nor sanctions. With regard to maternal employment, according to the ILO, female employees have been eligible for paid maternity leave of 12 weeks with full pay since 2009 (ILO, 2017). In the implementation research area, the Becoming Breastfeeding Friendly (BBF) Toolbox has been applied in Mexico three times since 2016 (Pérez‐Escamilla et al., 2022). Briefly, BBF is based on metrics to assess the strengths and weaknesses of the different components of the BFGM (Pérez‐Escamilla et al., 2012) to assess the country's readiness to scale up and make the corresponding evidence‐informed policy recommendations to enable the BF environment (González de Cosío et al., 2018). A result of the BBF multisectoral process since 2016 (Pérez‐Escamilla et al., 2012) is the strong advocacy for improving maternity leave benefits in Mexico for women employed in both the formal and informal economy (Vilar‐Compte et al., 2019).

Mexico's effort to improve infant feeding practices was initiated after the sharp decrease in BF practices shown by the National Health and Nutrition Survey in 2012 (Monitoring and evaluation) (Gutiérrez et al., 2012). This brought to the attention of key actors including civil society organizations the urgent need to act in favor of improving BF practices in the country. International organizations, Civil Society, Academia and Government allies started to identify strategies and opportunities to improve BF practices, a process that was strongly advanced by the BBF initiative as it has allowed them to work in a coordinated manner.

In the context of the sharp decline in BF practices in the country and evidence‐based BF advocacy, BF made its way into the government's agenda. In 2014, the Secretary of Health in Mexico promoted the reform of the ‘Ley General de Salud’ (Health General Law), which stipulates the mandatory set of actions that are needed to increasing EBF and the duration of any type of BF, including training of health professionals. As a result of this amendment, the 2013–2018 National Development Plan included the need to promote BF through actions to increase the duration of BF and EBF through BF promotion and support programmes and BF training of health personnel. It is in this context that the National Breastfeeding Strategy (ENLM, for its acronym in Spanish) was launched by the Secretary of Health and was implemented from 2014 to 2018 as the national breastfeeding policy (Secretaría de Salud, 2013). The ENLM had important limitations, such as not having an assigned budget, the lack of clarity on the proposed indicators for its evaluation and the lack of an implementation and impact evaluation strategy based on a proper study design. Nevertheless, KIs considered that the Strategy did advance the cause of BF in Mexico because it included actions and guidelines for health services nationwide to engage in promoting, protecting and supporting BF. In the same vein, various initiatives for amending laws and regulations emerged; for instance, the development of ‘Norm project 050’ ('Proyecto de norma 050') in 2018. Even though it has not yet been ratified, this norm is important because it sets out more specific criteria on actions that need to be taken for strengthening the protection, promotion and support of BF through 2 years of age, with a strong focus on supporting EBF during the first 6 months of life. This standard was proposed to be mandatory at the national level for health service personnel in the public, social and private sectors of the National Health System who were involved with maternal and child health care services, as well as for all persons, companies or institutions that had contact with mothers of infants and young children. BF women and those involved in the care, feeding and development of children (Diario Oficial de la Federación (DOF), 2018).

In Mexico, social mobilization, well coordinated work among various sectors and actors and evidence‐based advocacy represented a major step forward in the promotion, protection and support of BF. However, there are still major challenges to further improving BF practices in Mexico, especially due to the loss of political will as a result of the presidential transition (e.g., the government that came to power in 2018 has abandoned many of the BF policies and programmes that probably explain the doubling of EBF during the previous decade).

During the last decade, another important challenge identified was the lack of sufficient budget allocation to implement, monitor and evaluate strategies and initiatives to protect, promote and support BF, in accordance with the National Breastfeeding Strategy and other initiatives that were in place until recently. Finally, marketing of BMS is a common practice in Mexico, and very prominent via different communication channels including social media, in the context of a lack of enforcement of the WHO Code (Box 2).

#### 3.3.3 Case study #3: Philippines

The Philippines is a lower‐middle‐income country, located in the western Pacific Ocean, with a population of about 109,581,000 people in 2020, making it the twelfth most populated country in the world (The World Bank, 2020b). According to the 2019 Expanded National Nutrition Survey, EBF improved from 52.3% in 2013 to 56.4% by 2019 (FNRI‐DOST, 2019). Over the past decade, in the Philippines, BF protection, promotion and support have been included in many national multicomponent policies reflecting political commitment, the inclusion of BF as part of development strategies. Through the literature review and supplemented with the KI interviews, we were able to identify the most representative policies and programmes that have enabled the BF environment in the country (Appendix S5).

Based on formative research that included service observations in 2008, clinical practice guidelines for essential newborn care were developed following the GRADE approach. These guidelines were adopted by the Department of Health (DOH) Administrative Order 2009‐0025 with the objective of adopting new policies and the protocol ‘Essential Intrapartum and Newborn Care’ (EINC) (Sobel, Silvestre, et al., 2011). This protocol was key for institutionalizing a national package of cost‐effective time‐bound interventions, including immediate and thorough drying of the newborn; early and prolonged skin‐to‐skin contact (Moore et al., 2016); properly timed clamping and cutting of the cord 1 to 3 min after birth, and not separating the newborn from the mother for early BF initiation and rooming‐in. EINC became known as Early Essential Newborn Care (EENC) in the WHO Western Pacific Region in 2014 and is now popularly known as ‘Unang Yakap’ or ‘The First Embrace’ in the Philippines. The implementation of this standard of care is ongoing in eight additional countries in the region ‐ Cambodia, China, Lao PDR, Mongolia, Papua New Guinea, Solomon Islands, Vanuatu, Viet Nam ‐ and is being regularly monitored.

The maintenance of the aforementioned policy has been the result of advocacy by different actors to generate the political will and the resources needed for implementation and continuous monitoring of BF programmes on a large scale. The DOH, with support from the WHO Country Office, commissioned *Kalusugan ng Mag‐Ina, Inc*. ('Health of Mother and Child'), a local NGO to conduct the evaluation of EENC every 2 years. Each country's results will be compared with its own objectives or benchmarks and with the results of the other eight countries.

The Philippines' government enacted Executive Order 51 (EO51) or Philippine Milk Code in 1986 to regulate the marketing and distribution of BMS and established an Interagency Committee (composed of representatives from the DOH, Department of Trade and Industry, Department of Justice and the Department of Social Welfare and Development) to review advertising, promotional and marketing materials for products covered by the EO51. As a result, the country is substantially aligned with the Code (WHO, 2020b). The DOH is also implementing a monitoring system for reporting violations of the WHO Code, called Mother‐Baby Friendly Philippines. This monitoring was launched in 2017 and is implemented through an official database where families can report inappropriate marketing of BMS; however, sanctions against those who commit Code violations are still weak.

In the Philippines, advocacy and coordinated work, and collaboration among different stakeholders have made it possible to strengthen the legislation. For instance, the ‘Philippine Coalition of Advocates for Nutrition Security’ (PHILCAN) has strengthened the Philippines' legislation. It is formed by 12 members from NGOs and its work is based on developing a clear and inclusive agenda that responds to the country's needs, based on evidence and supportive of national policies (Banerjee, 2017). It also invests in cultivating relationships with the key members, local governments and all nutrition stakeholders to meet their objectives (Banerjee, 2017).

In the Philippines, there several revisions have been made legislation for protecting, promoting and supporting BF. The passage and enactment of the Republic Act No. 10028 (The Expanded Breastfeeding Promotion) in 2010, No. 11148 (The First 1000 Days Law) in 2018 and No. 11210 (105 Days Extended Maternity Leave Act) in 2019 was an important step toward strengthening BF through national legislation. Briefly, the Republic Act No. 10028 calls for BF breaks and designated facilities in the workplace. In addition, this act includes a provision that BF topics should be part of the curriculum of the education sector to support BF workforce development. On the other hand, Republic Act No. 11148 aims to scale up nutrition programmes to detect early and prevent or properly manage malnutrition among infants, young children, adolescent females, and pregnant and lactating women to foster the healthy growth and development of infants and young children. It is worth noting that this law aligns with the inclusion in the definition of BMS commerical milk including products children up to 36 months, as defined in the World Health Assembly Resolution 69.9. Laslty, Republic Act No. 11210, exhorts for extending paid maternity leave from 60–78 to 105 days with full pay, regardless of whether women have a vaginal or a cesarean section delivery.

Finally, the increase in the DOH's budget for child health services, including BF, is another clear example that BF is a priority for the Philippines government resulting from the advocacy‐driven strong political will. This is also illustrated through the inclusion of BF promotional activities, such as the creation of visual materials with the slogan ‘Breastfeeding T. S. Ek’ ('Correct, Adequate and Exclusive Breastfeeding'). Despite the strong legislation and the current Code monitoring system in the Philippines, there still and pervasive marketing of the BMS industry. In addition, there is a major challenge in the legislative process due to the involvement of the BMS industry in high‐level policy creating a conflict of interest; and the promotion of BMS on the internet including through social media by influencers. One area of opportunity for the legislation is to strengthen advocacy in communities to counter aggressive BMS marketing. Similarly, an opportunity area is for the official database for reporting violations of the Code is to ensure that it can be used by the entire population, regardless of their network access (Box 3).

#### 3.3.4 Case study #4: United States

The United States is a country located in North America that is among the world's largest countries by total area. It is made up of 50 states, one federal district, five large unincorporated territories, 326 Indian reservations and some smaller possessions, making it a country with a great variety of cultures and traditions (National Conference of State Legislatures [NCSL], 2018). BF rates in the United States have gradually increased in the past 20 years. EBF has improved from 49.4% in 2011 to 58.3% in 2017 (Centers for Disease Control and Prevention [CDC], 2011; CDC National Immunization Survey [NIS] 2018‐2019 for Center for Disease Control and Prevention (CDC), 2017). However, these estimates varied by race/ethnicity, mother's age and education, participation in the WIC Programme (The Special Supplemental Nutrition Program for Women, Infants and Children) and family income. National estimates indicate substantial differences between non‐Hispanic Black and non‐Hispanic White infants across BF indicators in the United States. Three quarters (76%) of Black infants are ever breastfed, which is below the national average of 84.1% (2017) (CDC, 2020). Although the great majority of infants started BF, only 58.3% of infants were breastfed at 6 months (CDC, 2020). For this reason, the United States has been actively implementing programmes and policies aimed at protecting, promoting and supporting BF (Appendix S5).

The CDC's Division of Nutrition, Physical Activity and Obesity has been strongly committed to increasing BF rates through promoting and supporting optimal BF practices over the past decades. The CDC plays an essential role in the research and monitoring of infant feeding practices in the United States. The ‘Breastfeeding Report Card’ provides data on BF practices and supports in the country every two years. Furthermore, BF data from the NIS continue to be released annually. CDC has also been monitoring maternity practices, which has helped identify areas of opportunity to strengthen BF‐related in‐hospital maternity care practices, staff skills, and discharge support. This is done through a biannual national hospital survey called Maternity Practices in Infant Nutrition and Care (mPINC). The CDC then provides specific feedback to hospitals so they can implement evidence‐based strategies to improve BF support. Additionally, CDC provides funding for interventions related to maternity care practices in the health sector to achieve the Baby‐Friendly designation and at the community level through REACH (Racial and Ethnic Approaches to Community Health) grants.

The United States has institutionalized programmes that have contributed for several decades to improving nutritional status and tackling food insecurity among the most vulnerable populations. The gradual decline in BF rates in the United States beginning in the early 20th century prompted the United States Congress to enact legislation to address malnutrition among low‐income pregnant, lactating, or post‐partum women and their infants and children. In 1966 the Child Nutrition Act was enacted, which in its Section 17 includes WIC Legislative Requirements. WIC was established as a permanent program in 1974 to the well being of income pregnant, BF and non‐BF post‐partum women and children up to age 5 years who are at nutritional risk (United States Department of Agriculture [USDA], 2013). It includes four main components: nutrition education (promotion campaigns), BF support, healthy food packages and referrals to health and social services (USDA, 2013). The BF Peer Counselling Programme adds a critical dimension to WIC's efforts by providing a valuable service to communities, by addressing some of the most common BF barriers through BF education, support and role modeling (National WIC Association, 2019).

Sustainability BF and initiatives as well as their monitoring are very strong in the United States as evidenced by the Baby‐Friendly Hospital Initiative (BFHI). Baby‐Friendly USA, the national authority overseeing this program, facilitates the health personnel training needed and provides technical assistance to hospitals in their pursuit of initial accreditation and subsequent reaccreditations. To achieve accreditation, hospitals must complete their ‘4D pathway’ approach (Discovery, Development, Dissemination and Designation), which includes the staff training plan to integrate the ‘Ten Steps to Successful Breastfeeding’ into their maternity practices. Baby‐Friendly designation is conferred for a period of 5 years; the 3 years post designation is the Annual Quality Improvement Phase to assist facilities to maintain compliance (sustainability), and the final 2 years are for the Redesignation Phase.

BF progress in the United States has been in part the result of well‐coordinated collaborative work that includes key stakeholders in BF promotion and support. In May 2011, the Federal Interagency Breastfeeding workgroup was created with members who represent 16 individual agencies (Anstey et al., 2016). It includes the Department of Labor, Office of Women's Health, CDC, USDA and National Institute of Health (NIH), among others. This workgroup provides a forum for information exchange across federal agencies and encourages collaborative approaches to address recommendations based on sound research conducted by the CDC and academia. The creation of this group helped to increase the capacity of the United States Breastfeeding Committee (USBC) and affiliated state coalitions. The USBC is an independent, nonprofit coalition of more than 100 influential professional, educational and governmental organizations, whose goal is to advance collaborative efforts to promote policies and practices that create an enabling environment for BF across the country (advocacy). Unlike other countries, International Organizations are not visible in the United States, except for the WHO with its participation through the BFHI.

Although the United States has a strong programme delivery and research and evaluation gears, some challenges to improve the BF environment were identified. Between 2016 and 2020 there were concerns about reductions in budget allocation for interventions that promote, protect and support BF due to major changes in government. However, the new political administration elected in 2020 is giving more priority and attention to maternal‐child public health issues, including BF.

The political will for strengthening support for BF in the country was recently crystalized in the 2020 Dietary Guidelines for Americans, the policy document upon which all federal food and nutrition policy is based, as for the first time it included feeding recommendations for children under two including a strong endorsment for BF. A key challenge in the United States is the lack of national laws for BF protection. While all 50 states, the District of Columbia, Puerto Rico and the Virgin Islands have laws that specifically allow women to breastfeed in any public or private locations, only 30 states, the District of Columbia and Puerto Rico have laws related to BF in the workplace. The Family and Medical Leave Act provides 12 weeks of unpaid leave to workers in companies with less than or equal to 50 employees. Currently, eight states (California, New Jersey, Rhode Island, New York, Washington, Massachusetts, Connecticut and Oregon) and Washington DC have enacted laws offering paid family leave; however, it is partially paid for between 6 and 12 weeks, which is way less than the ILO recommendation (ILO, 2000; NCSL, 2020).

In regard to the United States, it is worth highlighting that the collaborative work through the Federal Interagency group has indeed impacted all RE‐AIM dimensions; by including people of all ethnicities and income levels at the hospital and community levels (reach), the adoption and institutionalization of effective BF programmes including BFI and BF counseling (effectiveness and adoption) and sustainability in part because of stable budget allocations that have been successfully advocated for over time (maintenance) (Box 4).

### 3.4 Similarities and differences among countries

To reach the objective of the study, Table 4 summarizes the lessons learned from the four‐country case studies in different world regions. Based on the BFGM gears, key enabling factors and challenges to achieve the scaling up of policies, programmes and interventions to protect, promote and support BF were identified. In all four countries, evidence‐based advocacy, multisectoral political will, financing, research and evaluation, and coordination were key to fostering an enabling environment for BF. Furthermore, in all countries, major challenges were the lack of adequate maternity protection and the aggressive marketing of the breast‐milk substitutes.

**Table 4:** Main enabling factors and challenges for scaling up breastfeeding promotion, protection and support in four countries

	BFGM gear	Burkina Faso	Mexico	The Philippines	United States of America
Enabling factors	Advocacy	Coordinated advocacy (NGOs, International Organizations) (Stronger with Breast Milk Only Initiative).	Evidence‐based advocacy from International Organizations, Academia and Civil Society.	Coordinated work and collaborations with National and International Organizations and Government. Coordinated advocacy to strengthen and protect legislation through 'Philippine Coalition of Advocates for Nutrition Security' (PHILCAN).	Coordinated advocacy through the Federal Interagency Breastfeeding work group. Strong advocacy work by civil organizations, especially the United States Breastfeeding Committee.
	Political will	Present	Present	Present	Present
	Legislation and policies	Paid maternity leave (100% paid for 14 weeks). Moderately aligned with the Code and recent revisions to the decree no. 93‐279/PRES/SASF/MICM. National policy: National Infant and Young Child Feeding Scale Up Plan.	Paid maternity leave (100% paid for 12 weeks). Moderately aligned with the Code. National Breastfeeding policy: National Breastfeeding Strategy (2014–2018). The Law for the Protection of the Rights of Children and Adolescents promotes breastfeeding to ensure children's health. Specific Action Program on Sexual and Reproductive Health (2020–2024) in which perinatal health is a priority.	Paid maternity leave (100% paid for 15 weeks) and Family‐ Friendly workplace policies (including breastfeeding) (Republic Act Nos. 11148, 11210 and 10028). Substantially aligned with the Code. Executive Order 51 (Philippine Milk Code) regulates the marketing and distribution of BMS. Official database for reinforcement of the Code. National Policy (includes breastfeeding promotion, protection and support): Early Essential Newborn Care (EENC).	Child and Nutrition Act (1966) including WIC legislative requirements. Affordable Care Act, the comprehensive health care reform law enacted in March 2010 (sometimes known as ACA, PPACA, or 'Obamacare'). The PUMP for Nursing Mothers Act (S. 1658/H.R. 3110) would strengthen the Break Time for Nursing Mothers law by expanding workplace protections for lactating workers.
	Funding and resources	Outside funding mainly (International Organizations and NGOs) for health and nutrition activities.	Resources from International Organizations (UNICEF) for interventions and programmes related to breastfeeding.	The government grants a budget for different health and nutrition activities.	Through the Affordable Care Act, money was allocated to the CDC for the support and promotion of breastfeeding. Allocation of funds from government institutions for research on maternity care practices (i.e., Baby Friendly Hospital Initiative designation).
	Training and programme delivery	Group of midwives who contribute to breastfeeding training for health professionals. Courses organized by WHO, called 'Infant Young Child Feeding Counseling' to provide health professionals with the necessary skills to carry out effective breastfeeding counselling. Inclusion of traditional community leaders in training sessions. A cascade training for healthcare providers to promote the National Infant and Young Child Feeding (IYCF) scale up plan. Programmes of breastfeeding promotion at different levels (Health System and Community level).	The Integrated Strategy for Attention to Nutrition (ESiAN, for its acronym in Spanish) aimed to address the double burden of malnutrition and strengthen the quality of Primary Health Care including a training strategy, targeted to promote infant feeding practices according to current recommendations. The volunteers of 'La Leche League' receive important training on breastfeeding issues, breastfeeding techniques and breastfeeding problem solving to accompany women in their process. Training of health personnel as part of the Specific Action Programme on Sexual and Reproductive Health (2020–2024). The Government and PAHO organize courses for breastfeeding instructors to carry out cascade training in the entities.	Topics related to breastfeeding were incorporated into the curriculum from the basic level of studies up to the university level. Training for health professionals based on the ENNC protocol to achieve standardized intrahospital practices.	WIC program targeting low‐income families: Breastfeeding promotion and protection Baby Friendly Hospital Initiative (BFHI). WIC breastfeeding peer counsellor programme. USBC Constellation Work Groups by USBC with the goal of expanding training and mentoring opportunities for lactation care providers, particularly to increase the number of racial/ethnic minority care providers.
	Breastfeeding promotion	A national campaign promoting exclusive breastfeeding (*Strong with Breast milk only*).	Dissemination, communication and orientation about breastfeeding topics for different population groups at the community level as part of the Specific Action Programme on Sexual and Reproductive Health (2020–2024).	Mass activities: simultaneous mother and baby latch on. Breastfeeding support groups, such as the Facebook support group 'Breastfeeding Pinays'. Visual materials with the slogan 'Breastfeeding T. S. Ek' (Breastfeeding Correct, Adequate and Exclusive) by the Department of Health.	'Ban the Bags' campaign regarding not providing infant formula to nursing mothers at the time of hospital discharge.
	Research and evaluation	Research by international organizations (UNICEF and IBFAN) to identify the barriers and social norms that affect breastfeeding.	Group of different stakeholders (expertise on different areas of breastfeeding) evaluating breastfeeding environment, providing recommendations to decision‐makers. Publication of a book with recommendations to prevent all forms of malnutrition in children and adolescents, with a specific section on breastfeeding (National Institute of Public Health, FAO, PAHO and UNICEF).	Civil Organizations work with the Department of Health to create evidence‐based guidelines for Early Essential Newborn Care: 'Unang Yakap' (First Embrace).	National Organizations create a strong evidence base to protect and support breastfeeding.
	Coordination, goals and monitoring	The Ministry of Health carries out an annual national survey on nutrition, including infant and young child feeding indicators since 2012. Strong coordination by the Direction of Nutrition (Nutrition Directorate) in the Ministry of Health. Standardized nutrition and health messaging to ensure that the whole country receives the same information.	Monitoring of breastfeeding practices through different national surveys. Coordinated work between International Organizations and Academia to support breastfeeding policies in the workplace.	Monitoring of breastfeeding practices as well as policies and initiatives to promote, protect and support breastfeeding. Inclusion of skin‐to‐skin contact as an indicator in the National Demographic and Health Survey (NDHS). Monitoring system for reporting violations of the Code, called Mother‐Baby Friendly Philippines. The Department of Health coordinates all activities and initiatives for breastfeeding promotion, protection and support.	Monitoring of breastfeeding practices (Breastfeeding report cards) and Maternity care practices (Maternity Practices in Infant Nutrition Care). CDC Breastfeeding Report Card with state‐by‐state data to help public health practitioners work together to support breastfeeding. National Breastfeeding Committee coordinating initiatives and breastfeeding programmes, including different sectors (USDA, NIH and CDC).
Challenges		Lack of local funding and specific resources for breastfeeding promotion, protection and support. Presence of the BMS industry through promotions to the general population and among health professionals, as well as interference in the legal process. Maternity leaves are not aligned with ILO recommendation No. 191.	Limited budget allocation to implement, monitoring and evaluation of policies and programmes to protect, promote and support breastfeeding. BMS inappropriate marketing is common with no clear monitoring system nor effective and significant sanctions. Sustainability of policies and initiatives to promote, protect and support breastfeeding after a change of government.	Aggressive BMS marketing persists. Gap in regulations to prevent politicians from using breast‐milk substitutes products as a donation to buy popularity. Limited scope of the system for reporting violations of the Code, as the internet is required for its use.	Budget allocation for interventions to promote, protect and support breastfeeding affected by government change. None of the Code provisions has ever been adopted by national legislation. No national paid maternity protection, including breastfeeding protection for working women.

Abbreviations: BFGM, Breastfeeding Gear Model; BMS, breast‐milk substitutes; CDC, Centers for Diseases Control and Prevention; ILO, International Labour Organization; NGOs, nongovernment Organizations; NIH, National Institutes of Health; The Code, International Code of Marketing Breast milk Substitutes; USDA, United States Department of Agriculture; WIC, The Special Supplemental Nutrition Program for Women, Infants and Children.

## 4 DISCUSSION

The four‐country comparative case study, successfully identified how countries from different regions were able to successfully scale up national BF policies and programmes that were likely to have contributed to improvements in BF practices in the past decade. These findings are especially relevant because they happened in the context ​​of powerful commercial, economic and social barriers that the majority of women in the world still face in exercising their right to breastfeed their for long as they want to. The implementation pathways that each country has followed to protect, promote and support BF were unique as predicted by the Complex Adaptive Systems Framework (Paina & Peters, 2012). Nevertheless, key enablers were highly consistent with the BFGM including evidence‐based advocacy, political will, financing, workforce development, evidence‐based facility and community‐based program implementation, behavior change communications campaigns, research and evaluation and multisectoral coordination.

In Burkina Faso, their successful approach rests upon strong collaborative work among different stakeholders, including Government, International Organizations and NGOs, working as an alliance that goes beyond a tenuous partnership. This was idnetified as an innovative strength compared with other countries. Another distinctive feature of the process in Burkina Faso was the engagement of a government entity responsible, the Directorate of Nutrition, for improving the health and nutrition of the population, in this case focusing on infant feeding practices. Another key feature was the implementation of an evidence‐based BF policy advocacy strategy, which included an innovative and attractive social and behavior change communications campaign promoting EBF at the national level.

In the case of Mexico, several actions and initiatives were strongly driven or supported by the multisectoral BBF initiative (González de Cosío et al., 2018; Hromi‐Fiedler et al., 2019; Safon et al., 2018) whose launch coincided with the start of rapid EBF increases in the country (Unar‐Munguía et al., 2021). In the BBF process key stakeholders participated in a coordinated manner to promote, protect and support BF, such as evidence‐based advocacy, the first‐ever published position from the Mexican National Academy of Medicine calling for a national strategy based on the BFGM (González de Cosío‐Martínez & Hernández‐Cordero, 2016), government‐driven amendment of legislation for the protection of BF and the strengthening and implementation of the National Breastfeeding Strategy (Pérez‐Escamila et al., 2012).

In the Philippines, what distinguishes its process was the strong government commitment, supported by the implementation of a comprehensive national policy to promote health and nutrition during the first 1000 days of life, which includes the promotion, protection and support of BF, as a central part of this policy. Two very important achievements were the amendment of the Maternity Protection Act, to increase the duration of paid maternity leave in line with ILO's minimum standard and the strengthening of the enforcemnt of the national BMS Code.

In the United States, since there is no national health care system, the for BF from public health perspective, has largely fallen on CDC. The CDC has played a central role in working closely with Baby‐Friendly USA, building the BF workforce and providing technical assistance to states has worked closely with the US BF Committee facilitating BF advocacy efforts that have been essential for generating political will and necessary funding. The United States has been unique in the level of allocation of financial resources for research (e.g., Infant Feeding Practices Study (CDC, 2021a), monitoring (e.g., mPINC survey; BF rates monitoring with National Immunization survey [CDC, 2021b] and the BF report card [CDC, 2020] of BF practices) and the implementation of strategies to improve evidence‐based programmes such as BFHI and WIC program BF peer counseling. A unique feature in the United States is the Code has not been adopted and in general BF protection measures are still exceedingly weak.

In terms of findings that were common to all four countries, first, as the BFGM postulates, there is a need for more evidence‐based advocacy to generate the political will necessary to develop and pass legislation that releases fiscal resources for adequate protection, promotion and support of BF. In all four countries, the involvement of different stakeholders, at all levels, Government, Civil Society, Academia, and International Organizations, played an important role in the development and approval of policies and programmes to empower more women to breastfeed. Social mobilization and involvement of different stakeholders has been identified by others as a key factor in the process of scaling‐up action to improve nutrition (Gillespie et al., 2015). In Mexico, Burkina Faso and the Philippines, the collaborative and coordinated work of different stakeholders has placed BF on political, resulting in the strengthening of and development of policies and implementation programmes to promote, protect and support BF.

Even though there has been an important increase in EBF in the studied countries, all of them have important challenges that still need to be overcome. The aggressive and unethical marketing of BMS is one of the greatest challenges in all four countries. Unregulated BMS marketing is indeed a major public health concern because it has been proven to encourage commercial milk formula consumption at the expense of BF. Globally, it has been documented that mothers are heavily exposed to BMS promotion (Champeny et al., 2019; Hadihardjono et al., 2019; Hernández‐Cordero et al., 2019) and that this exposure influences their infant decision (Champeny et al., 2019; Parry et al., 2013; Piwoz & Huffman, 2015; Sobel, Iellamo, et al., 2011). In addition, BMS marketing has become increasingly multifaceted and involves high‐level political lobbying among other tacticts, thus requiring counteracting multi component and multilevel safeguards (Baker et al., 2021).

Improved legislation related to maternity leave is an area of opportunity for all four countries as currently the length of paid maternity leave is not in line with current ILO Recommendation No. 191, and in the case of Mexico and the United States, it is not aligned with either the ILO Convention No. 183 nor Recommendation No. 191. Where legislation is in place and improved, equitable coverage of this maternity protection entitlement should be ensured. Special attention needs to be paid to maternity protection for women working in the informal economy. This is key because more than half of women in Latin America, South Asia and sub‐Saharan Africa are employed in this sector. In Burkina Faso, Mexico, the Philippines and the United States, 65.7%, 52%, 52% and 14.8% of economically active women work in the informal sector, respectively (ILO, 2018; Ulep et al., 2021; Vilar‐Compte et al., 2019). Some countries have already assessed annual of maternity cash transfers for women with informal employment, ranging from US$87 million to US$280 million in Mexico from million to US$309 million in the Philippines (Ulep et al., 2021; Vilar‐Compte et al., 2019). In the case of the United States, there is no paid maternity leave policy at the federal level.

The low or lack of financial resources was another common barrier reported in the studied countries. As with other maternal‐child nutrition interventions, there is a need for adequate financial resources for scaling up protection, promotion and support of BF and implementing sound monitoring and evaluation systems to address both coverage and quality of implementation. The latter is crucial for improving decentralized decision‐making and the overall governance of national programmes. From the RE‐AIM framework perspective, financing used this way is key for maintaining or sustaining national BF policies and programmes. Finally, another important challenge, mentioned at least in two of the studied countries (Mexico and the United States), was the sustainability of policies and initiatives to protect, promote and support BF national programmes during government transitions. This is particularly important for policies that require long‐term processes for impact, including those aimed to contribute to infant feeding practices (Escobar‐Alegria et al., 2020).

Our study has some limitations. For Burkina Faso, it was not possible to reach two important stakeholders, government representatives and academia, in spite of repeated attempts to do so. However, with the support and information provided by other stakeholders and with available literature we were able to comprehensibly efforts done in the country, although compared with the other three countries we may have missed some level of detail. The use of information technology communication platforms for interviews with stakeholders might have previously been considered a limitation of the study; however, in the context of the global COVID‐19 pandemic, there is now ample evidence that sound qualitative research can be conducted at a distance using online information technology.

An innovative contribution and the main strength of this study was that we were able to systematically document key enabling factors for scaling up interventions, policies, or programmes to promote, protect and support BF using two complementary implementation sciences frameworks (Jilcott et al., 2007; Pérez‐Escamilla et al., 2012). The RE‐AIM framework (Jilcott et al., 2007) offered a structured and systematic approach to assessing the implementation of the most important programmes and policies in each country, while the BFGM (Pérez‐Escamilla et al., 2012) guided, following a dynamic systems approach, the identification of key facilitators and barriers for BF policies and programmes to work on a large scale and the stakeholders or institutions involved.

In through this comparative case studies analysis to better understand the distinct and common approaches that the four countries examined put into place to protect, promote and support BF. It is likely that such strategies explain at least in part the EBF improvements in each country over the last decade. In all four countries, a major challenge remaining is the need to strongly regulate the marketing of BMS that continues to prevent or slow down progress in BF outcomes globally (Baker et al., 2021; Hastings et al., 2020).

## AUTHOR CONTRIBUTIONS

The authors' responsibilities were as follows: Rafael Pérez‐Escamilla and Sonia Hernández‐Cordero designed the study, developed the protocol and the analytic approach. Rafael Pérez‐Escamilla, Sonia Hernández‐Cordero, Vania Lara‐Mejía and Bianca Franco‐Lares had full access to all the data in the study and took responsibility for the integrity of the data and accuracy of the data analysis. Sonia Hernández‐Cordero, Vania Lara‐Mejía, Bianca Franco‐Lares and Rafael Pérez‐Escamilla oversaw the primary data collection research, analyzed the data and wrote the first draft of the manuscript and had primary responsibility for the final content. Paul Zambrano and Isabelle Michaud‐Létourneau gave key insights during the protocol development and manuscript drafts. All authors read and approved the final version of the manuscript.

## CONFLICTS OF INTEREST

The authors declare no conflicts of interest.

## Supporting information

Supporting information.

Supporting information.

Supporting information.

Supporting information.

Supporting information.

## Data Availability

The data that support the findings of this study are available from the corresponding author upon reasonable request after receiving collaborative approval from the coauthors.

## References

[mcn13358-bib-0001] Anstey, E. H. , MacGowan, C. A. , & Allen, J. A. (2016). Five‐year progress update on the surgeon general's call to action to support breastfeeding, 2011. Journal of Women's Health, 25(8), 768–776. 10.1089/jwh.2016.5990 PMC5176340 27463691

[mcn13358-bib-0002] Baker, P. , Russ, K. , Kang, M. , Santos, T. M. , Neves, P. , Smith, J. , Kingston, G. , Mialon, M. , Lawrence, M. , Wood, B. , Moodie, R. , Clark, D. , Sievert, K. , Boatwright, M. , & McCoy, D. (2021). Globalization, first‐foods systems transformations and corporate power: A synthesis of literature and data on the market and political practices of the transnational baby food industry. Globalization and Health, 17(1), 58. 10.1186/s12992-021-00708-1 34020657 PMC8139375

[mcn13358-bib-0003] Banerjee, C. (2017). A new SUN civil society network: Advice from nutrition champions on set‐up. Nutrition Exchange, 7, 19. www.ennonline.net/nex/7/suncivilsocietynetwork

[mcn13358-bib-0004] Centers for Disease Control and Prevention (CDC) . (2011). National Immunization Survey (NIS). Retrieved March, 9th, 2022.

[mcn13358-bib-0005] Centers for Disease Control and Prevention (CDC) . (2017). National Immunization Survey (NIS). Retrieved March, 9th, 2022,

[mcn13358-bib-0006] Centers for Disease Control and Prevention (CDC) . (2020). *Breastfeeding report card*. Retrieved October 4, 2021, from https://www.cdc.gov/breastfeeding/data/reportcard.htm

[mcn13358-bib-0007] Centers for Disease Control and Prevention (CDC) . (2021a). *Studies of breastfeeding and infant feeding practices*. Retrieved October 11, 2021, from https://www.cdc.gov/breastfeeding/data/ifps/index.htm

[mcn13358-bib-0008] Centers for Disease Control and Prevention (CDC) . (2021b). *Breastfeeding rates*. National Immunization Survey (NIS). Retrieved October 11, 2021, from https://www.cdc.gov/breastfeeding/data/nis_data/index.htm

[mcn13358-bib-0009] Central Statistical Office and UNICEF . (2011). *Swaziland Multiple Indicator Cluster Survey 2010, final report*.

[mcn13358-bib-0010] Central Statistical Office and UNICEF . (2016). *Swaziland Multiple Indicator Cluster Survey 2014, final report*.

[mcn13358-bib-0011] Champeny, M. , Pries, A. M. , Hou, K. , Adhikary, I. , Zehner, E. , & Huffman, S. L. (2019). Predictors of breast milk substitute feeding among newborns in delivery facilities in urban Cambodia and Nepal. Maternal & Child Nutrition, 15(Suppl 4), e12754. 10.1111/mcn.12754 31225714 PMC6617748

[mcn13358-bib-0012] Diario Oficial de la Federación . (2018). *PROYECTO de Norma Oficial Mexicana PROY‐NOM‐050‐SSA2‐2018, Para el fomento, protección y apoyo a la lactancia materna*. Retrieved September 21, 2021, from https://www.dof.gob.mx/nota_detalle.php?codigo=5521251%26fecha=02/05/2018

[mcn13358-bib-0013] Escobar‐Alegria, J. L. , Frongillo, E. A. , & Blake, C. E. (2020). Terminal logic behavior and strategic defection of governmental officials during presidential transitions in Guatemala: Implications for the sustainability of food and nutrition security policy. Current Developments in Nutrition, 4(11), 161. 10.1093/cdn/nzaa161 PMC7792567 33447696

[mcn13358-bib-0014] Food and Nutrition Research Institute and Department of Science and Technology (FNRI‐DOST) . (2013). *Expanded National Nutrition Survey: 2013 results*.

[mcn13358-bib-0015] Food and Nutrition Research Institute and Department of Science and Technology (FNRI‐DOST) . (2019). *Expanded National Nutrition Survey: 2019 results*.

[mcn13358-bib-0016] Gillespie, S. , Menon, P. , & Kennedy, A. L. (2015). Scaling up impact on nutrition: What will it take? Advances in Nutrition, 6(4), 440–451. 10.3945/an.115.008276 26178028 PMC4496740

[mcn13358-bib-0017] González de Cosío, T. , Ferré, I. , Mazariegos, M. , & Pérez‐Escamilla, R. , BBF Mexico Committee . (2018). Scaling up breastfeeding programs in Mexico: Lessons learned from the becoming breastfeeding friendly initiative. Current Developments in Nutrition, 2(6), nzy018. 10.1093/cdn/nzy018 29955730 PMC6007513

[mcn13358-bib-0018] González de Cosío‐Martínez, T. , & Hernández‐Cordero, S. (2016). Lactancia materna en México. Recomendaciones para el diseño e implementación de una política nacional multisectorial de promoción, protección y apoyo de la lactancia materna en México (pp. 1–14). Academia Nacional de Medicina de México (ANMM). 10.21149/8102 28423117

[mcn13358-bib-0019] González‐Castell, L. D. , Unar‐Munguía, M. , Quezada‐Sánchez, A. D. , Bonvecchio‐Arenas, A. , & Rivera‐Dommarco, J. (2020). Situación de las prácticas de lactancia materna y alimentación complementaria en México: Resultados de la Ensanut 2018‐19. Salud Publica de Mexico, 62(6), 704–713. 10.21149/11567 33620967

[mcn13358-bib-0020] Gutiérrez, J. P. , Rivera‐Dommarco, J. , Shamah‐Levy, T. , Villalpando‐Hernández, S. , Franco, A. , Cuevas‐Nasu, L. , Romero‐Martínez, M. , & Hernández‐Ávila, M. (2012). *Encuesta Nacional de Salud y Nutrición 2012. Resultados Nacionales*. Instituto Nacional de Salud Pública.

[mcn13358-bib-0021] Hadihardjono, D. N. , Green, M. , Stormer, A. , Agustino, D. I. , & Champeny, M. (2019). Promotions of breastmilk substitutes, commercial complementary foods and commercial snack products commonly fed to young children are frequently found in points‐of‐sale in Bandung City, Indonesia. Maternal & Child Nutrition, 15(Suppl 4), e12808. 10.1111/mcn.12808 31225709 PMC6617717

[mcn13358-bib-0022] Hastings, G. , Angus, K. , Eadie, D. , & Hunt, K. (2020). Selling second best: How infant formula marketing works. Globalization and Health, 16(1), 77. 10.1186/s12992-020-00597-w 32859218 PMC7455895

[mcn13358-bib-0023] Hernández‐Cordero, S. , Lozada‐Tequeanes, A. L. , Shamah‐Levy, T. , Lutter, C. , González de Cosío, T. , Saturno‐Hernández, P. , Rivera Dommarco, J. , & Grummer‐Strawn, L. (2019). Violations of the international code of marketing of breast‐milk substitutes in Mexico. Maternal & Child Nutrition, 15(1), e12682. 10.1111/mcn.12682 30168899 PMC7199041

[mcn13358-bib-0024] Hernández‐Licona, G. , De la Garza, T. , Zamudio, J. , & Yaschine, I. (2019). El Progresa‐Oportunidades‐Prospera, a 20 años de su creación. CONEVAL. https://www.coneval.org.mx/Evaluacion/IEPSM/Documents/Libro_POP_20.pdf

[mcn13358-bib-0025] Hromi‐Fiedler, A. J. , Dos Santos Buccini, G. , Gubert, M. B. , Doucet, K. , & Pérez‐Escamilla, R. (2019). Development and pretesting of “Becoming Breastfeeding Friendly”: Empowering governments for global scaling up of breastfeeding programmes. Maternal & Child Nutrition, 15(1), e12659. 10.1111/mcn.12659 30211973 PMC7198937

[mcn13358-bib-0026] Instituto Nacional de Estadística e Informática . (2018). *Encuesta Demográfica y de Salud Familiar‐ENDES*.

[mcn13358-bib-0027] Instituto Nacional de Estadística e Informática . (2019). *Encuesta Demográfica y de Salud Familiar‐ENDES*.

[mcn13358-bib-0028] International Labour Organization (ILO) . (2000). *Maternity protection recommendation*. Retrieved September 20, 2021, from https://www.ilo.org/dyn/normlex/en/f?p=NORMLEXPUB:12100:0::NO::P12100_INSTRUMENT_ID:312529

[mcn13358-bib-0029] International Labour Organization (ILO) . (2017). *Conditions of work and employment programme*. Retrieved October 11, 2021, from https://www.ilo.org/dyn/travail/travmain.byCountry2

[mcn13358-bib-0030] International Labour Organization (ILO) . (2018). *Women and men in the informal economy: A statistical picture*.

[mcn13358-bib-0031] Jilcott, S. , Ammerman, A. , Sommers, J. , & Glasgow, R. E. (2007). Applying the RE‐AIM framework to assess the public health impact of policy change. Annals of Behavioral Medicine, 34(2), 105–114. 10.1007/BF02872666 17927550

[mcn13358-bib-0032] Ministère de la santé . (2012). Enquête Nutritionnelle Nationale 2012.

[mcn13358-bib-0033] Ministère de la santé . (2020). Enquête Nutritionnelle Nationale 2020.

[mcn13358-bib-0034] Moore, E. R. , Bergman, N. , Anderson, G. C. , & Medley, N. (2016). Early skin‐to‐skin contact for mothers and their healthy newborn infants. The Cochrane Database of Systematic Reviews, 11(11), CD003519. 10.1002/14651858.CD003519.pub4 27885658 PMC6464366

[mcn13358-bib-0035] National Conference of State Legislature (NCSL) . (2020). *Paid family leave resources*. https://www.ncsl.org/research/labor-and-employment/paid-family-leave-resources.aspx

[mcn13358-bib-0036] National Conference of State Legislatures . (2018). *The territories: They are us*. Retrieved October 2, 2021, from https://www.ncsl.org/Portals/1/Documents/magazine/articles/2018/SL_0118-Stats.pdf

[mcn13358-bib-0037] National Institute of Population Research and Training (NIPORT) & ICF International . (2019). *Bangladesh Demographic and Health Survey 2017–18: Key indicators*.

[mcn13358-bib-0038] National Institute of Population Research and Training (NIPORT), Mitra and Associates, & ICF International . (2016). *Bangladesh Demographic and Health Survey 2014*.

[mcn13358-bib-0039] National Statistical Office of Thailand and United Nations Children's Fund . (2013). *Thailand Multiple Indicator Cluster Survey*.

[mcn13358-bib-0040] National Statistical Office of Thailand and United Nations Children's Fund . (2016). *Thailand Multiple Indicator Cluster Survey*.

[mcn13358-bib-0041] National WIC Association . (2019). *WIC's promotion and support of breastfeeding—Making breastfeeding accessible and equitable for the WIC population*. https://s3.amazonaws.com/aws.upl/nwica.org/wics-promotion-and-support-of-breastfeeding.pdf

[mcn13358-bib-0042] Neves, P. , Vaz, J. S. , Maia, F. S. , Baker, P. , Gatica‐Domínguez, G. , Piwoz, E. , Rollins, N. , & Victora, C. G. (2021). Rates and time trends in the consumption of breastmilk, formula, and animal milk by children younger than 2 years from 2000 to 2019: Analysis of 113 countries. The Lancet Child & Adolescent Health, 5(9), 619–630. 10.1016/S2352-4642(21)00163-2 34245677 PMC8376656

[mcn13358-bib-0043] OECD . (2021). *Population (indicator)*. Retrieved September 24, 2021. 10.1787/5f958f71-en

[mcn13358-bib-0044] Paina, L. , & Peters, D. H. (2012). Understanding pathways for scaling up health services through the lens of complex adaptive systems. Health Policy and Planning, 27(5), 365–373. 10.1093/heapol/czr054 21821667

[mcn13358-bib-0045] Palestinian Central Bureau of Statistics . (2013). *Final report of the Palestinian Family Survey 2010*.

[mcn13358-bib-0046] Palestinian Central Bureau of Statistics (2015). *Palestinian Multiple Indicator Cluster Survey 2014, final report*.

[mcn13358-bib-0047] Parry, K. , Taylor, E. , Hall‐Dardess, P. , Walker, M. , & Labbok, M. (2013). Understanding women's interpretations of infant formula advertising. Birth, 40(2), 115–124. 10.1111/birt.12044 24635466

[mcn13358-bib-0048] Pérez‐Escamilla, R. , Curry, L. , Minhas, D. , Taylor, L. , & Bradley, E. (2012). Scaling up of breastfeeding promotion programs in low‐ and middle‐income countries: The “breastfeeding gear” model. Advances in Nutrition (Bethesda, Md.), 3(6), 790–800. 10.3945/an.112.002873 PMC3648703 23153733

[mcn13358-bib-0049] Pérez‐Escamilla, R. , & Hall Moran, V. (2016). Scaling up breastfeeding programmes in a complex adaptive world. Maternal & Child Nutrition, 12(3), 375–380. 10.1111/mcn.12335 27161881 PMC6860150

[mcn13358-bib-0050] Pérez‐Escamilla, R. , Vilar‐Compte, M. , Rhodes, E. , Sarmiento, O. L. , Corvalan, C. , Sturke, R. , & Vorkoper, S. (2021). Implementation of childhood obesity prevention and control policies in the United States and Latin America: Lessons for cross‐border research and practice. Obesity Reviews: An Official Journal of the International Association for the Study of Obesity, 22(Suppl 3), e13247. 10.1111/obr.13247 33951275 PMC8365637

[mcn13358-bib-0085] Pérez‐Escamilla, R. , Dykes, F. C. , & Kendall, S. (2022). Gearing to success with national breastfeeding programmes: The Becoming Breastfeeding Friendly (BBF) initiative experience. Matern Child Nutr, e13339. 10.1111/mcn.13339 35254735 PMC9835584

[mcn13358-bib-0051] Piwoz, E. G. , & Huffman, S. L. (2015). The impact of marketing of breast‐milk substitutes on WHO‐recommended breastfeeding practices. Food and Nutrition Bulletin, 36(4), 373–386. 10.1177/0379572115602174 26314734

[mcn13358-bib-0052] Richter, L. M. , Daelmans, B. , Lombardi, J. , Heymann, J. , Boo, F. L. , Behrman, J. R. , Lu, C. , Lucas, J. E. , Perez‐Escamilla, R. , Dua, T. , Bhutta, Z. A. , Stenberg, K. , Gertler, P. , & Darmstadt, G. L. , Paper 3 Working Group and the Lancet Early Childhood Development Series Steering Committee . (2017). Investing in the foundation of sustainable development: Pathways to scale up for early childhood development. Lancet (London, England), 389(10064), 103–118. 10.1016/S0140-6736(16)31698-1 PMC5880532 27717610

[mcn13358-bib-0053] Rollins, N. , Bhandari, N. , Hajeebhoy, N. , Horton, S. , Lutter, C. , Martines, J. , Piwoz, E. , Ritcher, L. , & Victora, C. , The Lancet Breastfeeding Series Group . (2016). Why invest, and what it will take to improve breastfeeding practices? Lancet, 387, 491–504. 10.1016/S0140-6736(15)01044-2 26869576

[mcn13358-bib-0054] Safon, C. , Buccini, G. , Ferré, I. , de Cosío, T. G. , & Pérez‐Escamilla, R. (2018). Can “Becoming Breastfeeding Friendly” impact breastfeeding protection, promotion, and support in Mexico? A qualitative study. Food and Nutrition Bulletin, 39(3), 393–405. 10.1177/0379572118789772 30111165

[mcn13358-bib-0055] Sankar, M. J. , Sinha, B. , Chowdhury, R. , Bhandari, N. , Taneja, S. , Martines, J. , & Bahl, R. (2015). Optimal breastfeeding practices and infant and child mortality: A systematic review and meta‐analysis. Acta Paediatrica, 104, 3–13. 10.1111/apa.13147 26249674

[mcn13358-bib-0056] Scaling Up Nutrition . (2021). *Burkina Faso*. Retrieved September 11, 2021, from https://scalingupnutrition.org/sun-countries/burkina-faso/

[mcn13358-bib-0057] Secretaría de Salud . (2013). *Estrategia Nacional de Lactancia Materna 2014–2018*. http://cnegsr.salud.gob.mx/contenidos/descargas/SMP/ENLM_2014-2018.pdf

[mcn13358-bib-0058] Shamah‐Levy, T. , Vielma‐Orozco, E. , Heredia‐Hernández, O. , Romero‐Martínez, M. , Mojica‐Cuevas, J. , Cuevas‐Nasu, L. , Santaella‐Castell, J. A. , & Rivera‐Dommarco, J. (2020). Encuesta Nacional de Salud y Nutrición 2018–19: Resultados Nacionales. Instituto Nacional de Salud Pública.

[mcn13358-bib-0059] Sobel, H. L. , Iellamo, A. , Raya, R. R. , Padilla, A. A. , Olivé, J. M. , & Nyunt‐U, S. (2011b). Is unimpeded marketing for breast milk substitutes responsible for the decline in breastfeeding in the Philippines? An exploratory survey and focus group analysis. Social Science & Medicine, 73(10), 1445–1448. 10.1016/j.socscimed.2011.08.029 21978633

[mcn13358-bib-0060] Sobel, H. L. , Silvestre, M. A. , Mantaring, J.,B., III , Oliveros, Y. E. , & Nyunt‐U, S. (2011a). Immediate newborn care practices delay thermoregulation and breastfeeding initiation. Acta Pædiatrica, 100, 1127–1133. 10.1111/j.1651-2227.2011.02215.x PMC3206216 21375583

[mcn13358-bib-0061] Statistical Institute of Belize & UNICEF Belize . (2012). *Belize Multiple Indicator Cluster Survey, 2011*.

[mcn13358-bib-0062] Statistical Institute of Belize & UNICEF Belize . (2017). *Belize Multiple Indicator Cluster Survey, 2015–2016*.

[mcn13358-bib-0063] Statistics Sierra Leone (SSL) and ICF International . (2014). Sierra Leone Demographic and Health Survey 2013. SSL and ICF International.

[mcn13358-bib-0064] Statistics Sierra Leone (Stats SL) and ICF . (2020). Sierra Leone Demographic and Health Survey 2019. Stats SL and ICF.

[mcn13358-bib-0065] Stronger With Breastmilk Only (SBWO) . (2021). *Stronger with breastmilk only*. Retrieved September 11, 2021, from https://www.breastmilkonly.com/

[mcn13358-bib-0066] The World Bank . (2020a). *World Bank Country and Lending Groups*. Retrieved October 2, 2021, from https://datahelpdesk.worldbank.org/knowledgebase/articles/906519-world-bank-country-and-lending-groups

[mcn13358-bib-0067] The World Bank . (2020b). *Population, total—Philippines*. Retrieved September 11, 2021, from https://data.worldbank.org/indicator/SP.POP.TOTL?locations=PH

[mcn13358-bib-0068] Ulep, V. G. , Zambrano, P. , Datu‐Sanguyo, J. , Vilar‐Compte, M. , Belismelis, G. , Pérez‐Escamilla, R. , Carroll, G. J. , & Mathisen, R. (2021). The financing need for expanding paid maternity leave to support breastfeeding in the informal sector in the Philippines. Maternal & Child Nutrition, 17(2), e13098. 10.1111/mcn.13098 33146460 PMC7988876

[mcn13358-bib-0069] Unar‐Munguía, M. , Lozada‐Tequeanes, A. L. , González‐Castell, D. , Cervantes‐Armenta, M. A. , & Bonvecchio, A. (2021). Breastfeeding practices in Mexico: Results from the National Demographic Dynamic Survey 2006–2018. Maternal & Child Nutrition, 17(2), e13119. 10.1111/mcn.13119 33325133 PMC7988861

[mcn13358-bib-0070] United Nations Children's Fund (UNICEF) . (2019). *The state of the world's children 2019 children, food and nutrition: Growing well in a changing world*. https://www.unicef.org/media/106506/file/The%20State%20of%20the%20World%E2%80%99s%20Children%202019.pdf

[mcn13358-bib-0072] United Nations Children's Fund (UNICEF) & World Health Organization (WHO) . (2017). *Nurturing the health and wealth of nations: The investment case for breastfeeding*. https://apps.who.int/nutrition/publications/infantfeeding/global-bf-collective-investmentcase.pdf?ua=1

[mcn13358-bib-0073] United States Department of Agriculture (USDA) . (2013). *About WIC: WIC's mission*. https://www.fns.usda.gov/wic/about-wic-wics-mission

[mcn13358-bib-0074] Victora, C. G. , Bahl, R. , Barros, A. J. , França, G. V. , Horton, S. , Krasevec, J. , Murch, S. , Sankar, M. J. , Walker, N. , & Rollins, N. C. , Lancet Breastfeeding Series Group . (2016). Breastfeeding in the 21st century: Epidemiology, mechanisms, and lifelong effect. Lancet (London, England), 387(10017), 475–490. 10.1016/S0140-6736(15)01024-7 26869575

[mcn13358-bib-0075] Victora, C. G. , Horta, B. L. , de Mola, C. L. , Quevedo, L. , Pinheiro, R. T. , Gigante, D. P. , & Barros, F. C. (2015). Association between breastfeeding and intelligence, educational attainment, and income at 30 years of age: A prospective birth cohort study from Brazil. The Lancet Global Health, 3(4), 199–205. 10.1016/S2214-109X(15)70002-1 PMC4365917 25794674

[mcn13358-bib-0076] Vilar‐Compte, M. , Teruel, G. , Flores, D. , Carroll, G. J. , Buccini, G. S. , & Pérez‐Escamilla, R. (2019). Costing a maternity leave cash transfer to support breastfeeding among informally employed Mexican women. Food and Nutrition Bulletin, 40(2), 171–181. 10.1177/0379572119836582 31035773

[mcn13358-bib-0077] Walters, D. D. , Phan, T. H. , & Mathisen, R. (2019). The cost of not breastfeeding: Global results from a new tool. Health Policy and Planning, 34, 407–417. 10.1093/heapol/czz050 31236559 PMC6735804

[mcn13358-bib-0078] World Health Organization (WHO) . (2012). Resolution WHA65.6. Comprehensive implementation plan on maternal, infant and young child nutrition. In *Sixty‐fifth World Health Assembly*. WHO Document Production Services. https://apps.who.int/iris/handle/10665/113048

[mcn13358-bib-0079] World Health Organization (WHO) . (2016). Maternal, infant and young child nutrition: Guidance on ending the inappropriate promotion of foods for infants and young children.

[mcn13358-bib-0080] World Health Organization (WHO) . (2020a). Marketing of breast‐milk substitutes: National implementation of the international code, status report 2020.

[mcn13358-bib-0081] World Health Organization (WHO) . (2020b). Global Nutrition Report 2020. Country Nutrition Profiles.

