# Supplementary material for: Countries' experiences scaling up national breastfeeding, protection, promotion and support programmes: Comparative case studies analysis
Source: Matern Child Nutr. 2022 Apr 19;18(Suppl 3):e13358. doi: 10.1111/mcn.13358 (PMC9113475; doi:10.1111/mcn.13358)
Supplement: Supplementary file 1 — Supporting information. [file MCN-18-e13358-s001.docx]

## **Supplementary Appendix 1:** Open-ended interview guide

**IN-DEPTH INTERVIEW GUIDE**

**(VIA ZOOM)**

**PROJECT INFORMATION**

**Directed to:** Key informants with expertise on policies, programs and actions implemented to improve infant feeding practices in 4 countries in 4 regions. Key informants will represent diverse sectors such as civil organizations, academia, government authorities and international organizations working in the protection, promotion and support of breastfeeding in each of the countries.

**Project title:** Breastfeeding practices improvement in the last decade in 4 countries: How did it happen?

**Objective of the instrument:** To learn the perspective of different actors on the factors that have favored the increase in breastfeeding practices in the country. As well as, from their experience, what are the challenges and barriers they still face to achieve the Breastfeeding recommendations of the World Health Organization.

**PRESENTATION**

Good morning/afternoon, ***interviewee's name***. Thank you for accepting the invitation to participate in this study. My name is ***researcher’s name***, currently I am a research assistant at the Health Department of the Universidad Iberoamericana; and as part of the project "Breastfeeding practices improvement in the last decade in 4 countries: How did it happen?" We are interested in knowing from your experience and the sector in which you work, the perspective on the main enabling factors that have favored breastfeeding practices on ***country’s name***. We would also like to know, from your experience, what are the challenges and barriers the country still faces in complying with the recommendations of the World Health Organization.

The information you provide will be kept confidential and will only be used for project purposes only. The interview will last approximately 50-60 minutes. It is my obligation to inform you that you are completely free not to answer any questions or to end the interview if you choose so; however, it is important that you provide me with as much information as possible to obtain a more complete record. As mentioned in the letter of consent, we request your authorization to record this interview. May I start the recording?

**IDENTIFICATION CARD**

**ID: |__|__||__|**

**Date of the survey: |__|__|__|__|__|__|**

**Day/Month/Year**

**City and Country:**

**Email:**

**Institution/Organization name:**

**Current position:**

| **Time in current position: \|__\|__\| years**    **Start time: \|__\|\|__\| : \|__\|\|__\|**    **Type of actor:** | **Age: \|__\|__\| Sex: \|__\|**    **End time: \|__\|\|__\| : \|__\|\|__\|** |
| --- | --- |

**CONTEXT**

**1. According to national/international survey data, in *country’s name*, there has been an increase in breastfeeding practices (especially exclusive breastfeeding). What factors do you consider to have contributed to this increase?**

● Who/what has contributed to the existence of these factors?

*In case the informant mentions an intervention/program/action ask*: In what year was it implemented? Who had the initiative? Who implements it? What is it about (What was about? *in case it is no longer implemented*)? Who is the target population? What are the characteristics of the population? Does it cover the target population?

● Is it still ongoing? *(is/was it at the local, state, national level)?*

o *If it is continuing and is it at the local or state level:* Are there plans to scale it up to the national level?

o *If no longer implemented: Why was it no longer implemented?*

● *In case the informant mentions an intervention/program/action ask*: Are there studies, publications or documents where such action/program/policy is described in detail, as well as the results and impact of the policy/program/intervention?

o *If yes*: Is the information available to the general public?

● *If the informant mentions something different than interventions, programs or policies (e.g., is there political will to improve breastfeeding practices) ask:* Whose initiative was it? Who was involved? What facilitators existed at that time to make it happen?

**2. In *country’s name*, which actors (government, civil organizations, academia and international organizations) have been or are involved in the protection, promotion and support of breastfeeding?**

● *Ask each stakeholder mentioned:* what is their participation?

● Are any of the actions/initiatives involving more than one of the actors?

o *If yes: What action/initiative is it? What is it about?*

o *If not: From your perspective, why has it not been possible to carry out/continue with any coordinated action among various actors?*

● *Identify among the different actors, which one was not mentioned (e.g., no mention of government, civil organizations, academia, international organizations), and ask:* From your perspective, why do you consider that ***actor’s name*** has not been involved in the actions/initiatives that have occurred?

**3. Although breastfeeding practices (especially exclusive breastfeeding) have improved in recent years, they are still below the current recommendation. What factors/events/conditions (political, social, economic) do you consider that represent a challenge to achieve actions, policies or programs that favor the protection, promotion and support of breastfeeding in *country’s name*?**

● *For each factor mentioned:* Why do you consider it as a challenge?

**4. Do you consider that in *country’s name* infant feeding practices, especially breastfeeding, are a priority in terms of policy development, programs or interventions and budget allocation?**

● *If yes:* In your opinion, which actions/factors support your perception that it is a priority?

● *If no:* In your opinion, which actions/factors support your perception that it is not a priority?

**CLOSING**

**5. We have finished with the questions*;* would you like to add anything else to the interview?**

**If we need any additional information, could we contact you again?**

**FAREWELL**

**Thank you very much for your time and participation! I will stop the recording.**
