# Supplementary material for: Countries' experiences scaling up national breastfeeding, protection, promotion and support programmes: Comparative case studies analysis
Source: Matern Child Nutr. 2022 Apr 19;18(Suppl 3):e13358. doi: 10.1111/mcn.13358 (PMC9113475; doi:10.1111/mcn.13358)
Supplement: Supplementary file 2 — Supporting information. [file MCN-18-e13358-s004.docx]

## **Supplementary Appendix 2:** Informed consent for the in-depth interview

**Informed Consent**

**In-depth Interview**

**Directed to:** Key informants with experience on policies, programs and actions implemented to improve infant feeding practices in the 4 selected countries.

**Title of the project:** Breastfeeding practices improvement in the last decade in 4 countries: How did it happen?

**Date of approval by Ethics Committee:** May 17^th^, 2021

**Introduction/Objective:**

Dear expert, we are inviting you to participate in a research project led by researchers from the Universidad Iberoamericana (IBERO) in Mexico and Yale University in the United States of America (USA). This project has the objective of documenting policies, programs and actions to promote, protect and support breastfeeding in 4 countries located in different regions of the world (Asia, Latin America, North America, and Sub-Saharan Africa).

We asked for your participation in the study because of your experience and interest in improving child feeding practices through policies, programs and actions implemented in some of the selected countries.

**Process:**

Your participation will consist of:

● Answer an in-depth interview with an approximate duration of 50-60 minutes. It will cover several questions related to knowledge, beliefs and attitudes about policies, programs and actions to improve infant feeding practices in the country. Also, about suggestions or recommendations that you consider relevant on the subject.

● The interview will be conducted through the Zoom platform on the day and time most convenient for you.

● If you agree and to facilitate the analysis, the audio will be recorded.

**Benefits:**

You will not receive any direct benefit for your participation in the study, however, if you accept, you will be collaborating with IBERO and Yale University to generate new knowledge about the implementation and strengthening of policies, programs and actions that support breastfeeding practices.

**Potential Risks/Compensation:**

Your participation in this study does not imply any risk to you, however, if any of the questions make you feel uncomfortable, you have the right not to respond. It is important to mention that you will not receive any payment for participating in the study, and it will not imply any cost to you.

**Confidentiality:**

All information you provide throughout this study will be kept strictly confidential and will only be known to the researchers of this project. Furthermore, it will not be made available for any other purpose. Researchers from Yale University in the United States of America are participating in this project; however, the information collected will NOT be transferred abroad. All the information will be processed, analyzed and summarized in Mexico, the only thing that will be shared is the summary of the information, for the development of reports and publications. You will be identified with a folio, so that your name will be disassociated from the information provided. The results of this study will be published for scientific purposes but will be presented in such a way that you cannot be identified.

During the interview transcriptions, names and any information that allows the identification of a person will be omitted, a general reference will be used (e.g., “Person A”, “Person B”, etc.). All the information collected will be stored in a computer with a security code, to which only the research team will have access. The informed consent will be protected separately. The recordings and identifying information will be destroyed once the research project ends.

**Voluntary participation:**

Your participation in this study is completely voluntary, therefore, you are free to refuse to answer any of the questions or revoke your consent at any time. Your decision to participate or not in the study will not imply any consequence.

**Simplified Privacy Notice:**

Your personal data will be processed, for the purposes described above, in accordance with the provisions of the personal data protection laws in force in Mexico, so you can exercise your ARCO rights: access, rectification, cancellation or opposition. In any of these cases, we ask you to contact the researcher responsible for the project at the following email address: sonia.hernandez@ibero.mx.

**Doubts:**

If you have any questions or comments regarding the study, you can contact Dr. Sonia Hernández Cordero, researcher at the IBERO Health Department in Mexico City, at 55 5950 4000 Ext. 4649 (weekdays at 9:00-17:00 hrs.), or if you prefer, by email sonia.hernandez@ibero.mx. Also, any disagreement with the project may be submitted to the President of the IBERO Research Ethics Committee: Dr. Mariana Dobernig Gago at 55 5950 4000 Ext. 4651 or by email mariana.dobernig@ibero.mx and/or claudia.reyes@ibero.mx

If you agree to participate in the study, we will provide you with a copy of this document, which we kindly ask you to sign.

| Declaration of the person giving consent:  ● I have read this consent form.  ● The research study has been explained to me including the purpose, possible risks and benefits, and other aspects of my participation in the study.  ● I have been able to ask questions related to my participation in the study, and my doubts have been answered satisfactorily.    If you agree to participate in this study, in whole or in part, and you also agree to allow your information to be used as described above, we ask you to indicate your consent to participate in this study.    **PARTICIPANT:**    **Name:** ______________________________________________________________    **Signed:** _________________________    **Date/Hour:** __________________ |
| --- |

| **NAME AND SIGNATURE OF RESEARCHERS OR PERSONS OBTAINING CONSENT:**    **WITNESS 1:**    **Name:** ______________________________________________________________    **Signed:**  _________________________    **Date/Hour**: __________________    **WITNESS 2:**    **Name:** ______________________________________________________________    **Signed:**  _________________________    **Date/Hour**: __________________ |
| --- |
