# Supplementary material for: Countries' experiences scaling up national breastfeeding, protection, promotion and support programmes: Comparative case studies analysis
Source: Matern Child Nutr. 2022 Apr 19;18(Suppl 3):e13358. doi: 10.1111/mcn.13358 (PMC9113475; doi:10.1111/mcn.13358)
Supplement: Supplementary file 3 — Supporting information. [file MCN-18-e13358-s003.docx]

**Supplementary Appendix 3:** Coding book based on the “Breastfeeding Gear Model” gears used for the qualitative analysis of in-depth remote semi-structured interviews

| ***Node*** | ***Subnode*** | ***Description*** |
| --- | --- | --- |
| ***Advocacy*** | *Civil organizations supporting advocacy* | Civil organizations that help advocacy for the implementation of policies or programs that support BF. |
|  | *Academia supporting advocacy* | Academic institutions that help advocacy for the implementation of policies or programs that support BF. |
|  | *International organizations supporting advocacy* | International institutions that help advocacy for the implementation of policies or programs that support BF. |
| ***Political will*** | *Government priority* | Government priority for the development of programs or/and interventions. |
|  | *Enabling political factors* | Political factors that contribute to achieve actions, policies or programs that favor the protection, promotion, and support of BF. |
|  | *Political barriers* | Political factors that represent a challenge to achieve actions, policies or programs that favor the protection, promotion, and support of BF. |
| ***Legislation & policies*** | *Breastfeeding laws* | Laws for the promotion, support, and protection of BF. |
|  | *Workplace breastfeeding laws* | Laws for the promotion, support, and protection of BF in the workplace. |
|  | *Breastfeeding policies* | Policies for the promotion, support, and protection of BF. |
|  | *Breastfeeding programs* | Programs for the promotion, support, and protection of BF. |
|  | *Marketing of BMS* | Laws and policies that influence the regulation of the promotion of BMS, and the activities of BMS companies. |
|  | *Scale up or sustainability* | Scale up or sustainability of programs for the promotion, support, and protection of BF. |
|  | *Policies or programs barriers* | Barriers or challenges related with the policies or programs for the promotion, support, and protection of BF. |
|  | *Positive and negative feedback loops* | Testimonials on how the barriers, challenges or bottlenecks were overcome. |
| ***Funding & resources*** | *Government budget allocation* | Funding and the availability of economic resources to transform legislation into programs with concrete actions by the government. |
|  | *International organization budget allocation* | Resources or funding for interventions/actions/programs provided by international organizations. |
|  | *Economical factors* | Economic factors that contributed to achieve actions, policies or programs that favor the protection, promotion, and support of BF. |
|  | *Funding barriers* | Economic factors that represent a challenge to achieve actions, policies or programs that favor the protection, promotion, and support of BF. |
| ***Training & program delivery*** | *Health professional training* | Training actions for health professionals for the promotion, support, and protection of BF. |
|  | *Midwives and peer counselors training* | Training actions for midwives and peer counselors for the promotion, support, and protection of BF. |
|  | *Counseling for parents* | Counseling for parents for the promotion, support, and protection of BF. |
|  | *Training or counseling challenges* | Challenges or barriers related with health professional training or counseling for parents. |
| ***Promotion*** | *Promotional campaigns* | Campaigns for the promotion, support, and protection of BF. |
| ***Research & evaluation*** | *Scientific research for program/intervention development* | Scientific research for the development of programs or interventions for the promotion, support, and protection of BF. |
|  | *Program/intervention/action evaluation* | Program/intervention/action evaluation process. |
|  | *Research and evaluation barriers* | Research and evaluation barriers. |
| ***Coordination, goals & monitoring*** | *Program/intervention/action goals* | Goals and successes achieved toward stated goals of the program/intervention/action. |
|  | *Program/intervention/action monitoring* | Program/intervention/action monitoring process. |
|  | *Coordinated program/intervention/action* | Actions/initiatives/programs involving more than one of the actors (government, civil organizations, academia, and international organizations). |
|  | *Coordination and monitoring barriers* | Program/intervention/action coordination and monitoring barriers or challenges. |

BF: Breastfeeding

BMS: Breast Milk Substitutes
