# Supplementary material for: Countries' experiences scaling up national breastfeeding, protection, promotion and support programmes: Comparative case studies analysis
Source: Matern Child Nutr. 2022 Apr 19;18(Suppl 3):e13358. doi: 10.1111/mcn.13358 (PMC9113475; doi:10.1111/mcn.13358)
Supplement: Supplementary file 4 — Supporting information. [file MCN-18-e13358-s005.docx]

## **Supplementary Appendix 4:** Complete characteristics of the key informant sample

| ***Country*** | ***Sex*** | ***Age*** | ***Sector*** | ***Organization*** | ***Time in current position (years)*** |
| --- | --- | --- | --- | --- | --- |
| ***Burkina Faso*** | Female | 58 | International Organization | UNICEF | 3 |
|  | Male | 49 | International Organization | Alive and Thrive Burkina Faso | 4 |
|  | Male | 53 | International Organization | IBFAN Afrique | 9 |
| ***Mexico*** | Male | 45 | International Organization | UNICEF | 7 |
|  | Female | 40 | Academy | Instituto Nacional de Salud Pública | 3 |
|  | Female | 48 | Academy | Universidad Iberoamericana | 6 |
|  | Female | 33 | Government | Ministry of Health | 1 |
|  | Female | 39 | Civil Society | Pacto por la Primera Infancia | 5 |
| ***Philippines*** | Male | 45 | Government | Food and Nutrition Research Institute | 0.11 |
|  | Female | 62 | Civil Society | Kalusugan ng Mag-ina, Inc. | 11 |
|  | Female | 51 | Civil Society | Nutrition Center of the Philippines | 7 |
|  | Female | 52 | Government | Ministry of Health | 1 |
|  | Female | 59 | International Organization | UNICEF | 10 |
| ***United States of America*** | Female | 57 | Civil Society | Baby-Friendly USA | 0.6 |
|  | Female | 59 | Government | U.S. Department of Agriculture | 6 |
|  | Female | 47 | Civil Society | United States Breastfeeding Committee | 1 |
|  | Female | 33 | Government | National WIC Association | 5 |
|  | Female | 44 | Government | CDC | 6 |
