# Supplementary material for: Countries' experiences scaling up national breastfeeding, protection, promotion and support programmes: Comparative case studies analysis
Source: Matern Child Nutr. 2022 Apr 19;18(Suppl 3):e13358. doi: 10.1111/mcn.13358 (PMC9113475; doi:10.1111/mcn.13358)
Supplement: Supplementary file 5 — Supporting information. [file MCN-18-e13358-s002.docx]

**Supplementary Appendix 5:** RE-AIM analysis of policies, programs, and interventions to promote breastfeeding in the 4 selected countries

1. ***Burkina Faso***

**Supplementary table 1.** Key elements linked Exclusive Breastfeeding Promotion by Peer Counsellors (PROMISE-EBF) in Burkina Faso from a perspective of the RE-AIM

| ***RE-AIM Element*** | ***Exclusive Breastfeeding Promotion by Peer Counsellors (PROMISE-EBF)*** |
| --- | --- |
| ***REACH*** | |
| Description of target population (geographic coverage, numbers targeted, demographic characteristics) | Pregnant women and mother-infant pairs |
| Setting characteristics (urban vs. rural, etc.) | Rural and urban setting in Burkina Faso, Uganda, and South Africa. |
| What percent of potentially eligible participants a) were excluded, b) took part and c) how representative were they? | - |
| Characteristics of participants reached compared to non-participants or to target population | - |
| Other factors that affect the reach of the program, including policy context, program budget constraints, conflict, and cost. | - |
| ***EFFECTIVENESS*** | |
| What were the goals of the intervention? | Increase the prevalence of EBF and decrease the prevalence of diarrhea to improve child growth through counselling by peer-counsellors. |
| What were the successes achieved toward stated goals of the program? | The intervention itself was a major contributor to  changes in early infant feeding practices. The intervention was associated with less prelacteal  feeding.  The one-week training course equipped the peer counsellors with basic information on promoting and supporting EBF, thus increasing EBF prevalence substantially in Burkina Faso and Uganda. However, PROMISE EBF trials found no effect on diarrhea morbidity (1) |
| What impact did the intervention have on all participants who began the program? | Overall, peer counselling was an effective strategy to increase the practice of EBF in Burkina Faso, Uganda, and South Africa. This effect was particularly important in Burkina Faso and Uganda.  At the infant age of 12 weeks, about a third of mothers breastfeed exclusively in the control clusters in these two countries compared with about 80% in the intervention clusters. (1) |
| What was the effect on process intermediate, and primary outcomes? | The results of PROMISE EBF show that EBF promotion by peer counsellors more than doubled the proportion of mothers who reported to have exclusively breastfed their infants. (1) |
| Possible negative or unintended consequences of the intervention on quality of life and related factors | - |
| Economic impact | - |
| ***ADOPTION*** | |
| What level was the program/intervention targeting (e.g., individual, community, policy)? How representative were the participating settings? | Community level |
| Description of regulations and legislation and how these affected adoption and continued use | The South African Department of Health’s routine child health services have a history of promoting commercial infant formula as part of the protein energy malnutrition scheme. ​​Additionally, the national programme for prevention of mother-to-child transmission of HIV-1 provided free formula to HIV-infected mothers. |
| Other factors important to adoption | The course material was based on the WHO courses: Breastfeeding counselling: a training course, and HIV and infant feeding counselling: a training course. The courses were integrated and adapted at sites for local circumstances.  Mothers with any breastfeeding difficulties were referred to a health worker with training in lactation management (Burkina Faso and Uganda) or to a public health provider who was aware of the HIV status of the participant (South Africa). |
| ***IMPLEMENTATION*** | |
| Description of implementation strategy including underlying theory, if any, and how it may be integrated with any other interventions | Pregnant women received counselling by peers supporting and promoting exclusive breastfeeding from late pregnancy throughout the first half year of their babies’ lives. They receive at least five visits, starting with a visit in the third trimester. The peer counsellors supporting and promoting EBF for six months were from the same communities as the mothers. They were trained in a one-week course by the national research teams with a curriculum from the WHO courses ‘Breastfeeding Counselling: A Training Course’ and ‘HIV & Infant Feeding Counselling: a Training Course’ adapted for the sites. |
| Cost of intervention (time or money) from the implementer perspective | - |
| Consistency of implementation across staff/time/settings/subgroups (not about differential outcomes, but process) | - |
| Adaptations made to intervention during program/project roll out (i.e., was the intervention delivered as intended?) | - |
| Other factors important to implementation, including policy and regulatory environment. | - |
| ***MAINTENANCE*** | |
| To what extent were different intervention components continued or institutionalized? How was the original program modified? | - |
| Availability/accessibility of intervention over time, and importance of these factors to adoption and sustained use | - |
| Is the intervention still ongoing at ≥ 12 months? | - |
| Description of any long-term subsidies/incentives and plans for continuity or phase-out, and their effects on adoption/sustained use | - |
| References:  1. Tylleskär T, Jackson D, Meda N, Engebretsen IMS, Chopra M, Diallo AH, et al. Exclusive breastfeeding promotion by peer counsellors in sub-Saharan Africa (PROMISE-EBF): A cluster-randomised trial. Lancet [Internet]. 2011;378(9789):420–7. Available from: http://dx.doi.org/10.1016/S0140-6736(11)60738-1  2. Engebretsen IMS, Nankabirwa V, Doherty T, Diallo AH, Nankunda J, Fadnes LT, et al. Early infant feeding practices in three African countries: The PROMISE-EBF trial promoting exclusive breastfeeding by peer counsellors. Int Breastfeed J. 2014;9(1).  3. Engebretsen et al.: Growth effects of exclusive breastfeeding promotion by peer counsellors in sub-Saharan Africa: the cluster-randomised PROMISE EBF trial. BMC Public Health 2014 14:633. | |

**Supplementary table 2.** Key elements linked to Training primary healthcare providers in Burkina Faso from a perspective of the RE-AIM

| ***RE-AIM Element*** | ***Training primary healthcare providers to deliver patient-centered, facility-based nutrition counseling*** |
| --- | --- |
| ***REACH*** | |
| Description of target population (geographic coverage, numbers targeted, demographic characteristics) | Health care providers |
| Setting characteristics (urban vs. rural, etc.) | National level (urban and rural) |
| What percent of potentially eligible participants a) were excluded, b) took part and c) how representative were they? | It was at the national level, so it applies to all health professionals. |
| Characteristics of participants reached compared to non-participants or to target population | - |
| Other factors that affect the reach of the program, including policy context, program budget constraints, conflict, and cost. | There is not sufficient money or funding to scale up activities. * |
| ***EFFECTIVENESS*** | |
| What were the goals of the program? | To improve communication between care providers and women at any contact for prenatal visits and children's services; and to enhance the nutrition component of the existing maternal and child national program. |
| What were the successes achieved toward stated goals of the program? | - |
| What impact did the intervention have on all participants who began the program? | Significantly more children in the intervention arm were exclusively breastfed for the first 6 months (54.3% vs 42.3; DP 12.8%; 95% CI: 2.1, 23.6; P = 0.020).  Complementary feeding practices were significantly better in the intervention arm, with the exception of timely introduction of solid, semi-solid, or soft foods. |
| What was the effect on process intermediate, and primary outcomes? | Early breastfeeding practices were better in the intervention arm. |
| Possible negative or unintended consequences of the intervention on quality of life and related factors | - |
| Economic impact | - |
| ***ADOPTION*** | |
| What level was the program/intervention targeting (e.g., individual, community, policy)? How representative were the participating settings? | Community level |
| Description of regulations and legislation and how these affected adoption and continued use | - |
| Other factors important to adoption |  |
| ***IMPLEMENTATION*** | |
| Description of implementation strategy including underlying theory, if any, and how it may be integrated with any other interventions | The nutrition counseling intervention was implemented in the intervention centers within the usual care environment. The intervention aimed to: i) improve communication between care providers and women at any contact for prenatal visits and children's services; and ii) enhance the nutrition component of the existing maternal and child national program, which includes prenatal care, immunization, and healthy and sick child consultations.  Before the study, all healthcare providers in the intervention arm were trained in communication and nutrition at the district hospital in April 2009. |
| Cost of intervention (time or money) from the implementer perspective | - |
| Consistency of implementation across staff/time/settings/subgroups (not about differential outcomes, but process) | - |
| Adaptations made to intervention during program/project roll out (i.e., was the intervention delivered as intended?) | - |
| Other factors important to implementation, including policy and regulatory environment. | - |
| ***MAINTENANCE*** | |
| To what extent were different intervention components continued or institutionalized? How was the original program modified? | - |
| Availability/accessibility of intervention over time, and importance of these factors to adoption and sustained use | - |
| Is the intervention still ongoing at ≥ 12 months? | Ongoing |
| Description of any long-term subsidies/incentives and plans for continuity or phase-out, and their effects on adoption/sustained use | The intervention has resources by NGO´s. |
| Reference:  1. Nikièma L, Huybregts L, Martin-Prevel Y, Donnen P, Lanou H, Grosemans J, et al. Effectiveness of facility-based personalized maternal nutrition counseling in improving child growth and morbidity up to 18 months: A cluster-randomized controlled trial in rural Burkina Faso. PLoS One. 2017;12(5):1–26.  *KI 03B | |

**Supplementary table 3.** Key elements linked Alive & Thrive initiative on exclusive breastfeeding in Burkina Faso from a perspective of the RE-AIM

| ***RE-AIM Element*** | ***Alive & Thrive initiative on exclusive breastfeeding*** |
| --- | --- |
| ***REACH*** | |
| Description of target population (geographic coverage, numbers targeted, demographic characteristics) | Mother-infant pairs |
| Setting characteristics (urban vs. rural, etc.) | Rural setting |
| What percent of potentially eligible participants a) were excluded, b) took part and c) how representative were they? | - |
| Characteristics of participants reached compared to non-participants or to target population | - |
| Other factors that affect the reach of the program, including policy context, program budget constraints, conflict, and cost. | Harmful norms surrounding infant feeding, such as feeding water and infusions to very young infants, persist in Burkina Faso. |
| ***EFFECTIVENESS*** | |
| What were the goals of the program? | To increase exclusive breastfeeding prevalence in areas reached by the initiative to at least 50% of infants younger than 6 months |
| What were the successes achieved toward stated goals of the program? | A difference of 38·9% (95% CI 32·2–45·6, p<0·001) in the reported prevalence of exclusive breastfeeding between the control and intervention groups at endline. |
| What impact did the intervention have on all participants who began the program? | The locally-delivered components of the Alive & Thrive initiative improved the knowledge, attitudes, and mothers’ reporting of exclusive breastfeeding practices by the time of the endline survey. |
| What was the effect on process intermediate, and primary outcomes? | Prevalence of exclusive breastfeeding: |
| Possible negative or unintended consequences of the intervention on quality of life and related factors | - |
| Economic impact | - |
| ***ADOPTION*** | |
| What level was the program/intervention targeting (e.g., individual, community, policy)? How representative were the participating settings? | Community level |
| Description of regulations and legislation and how these affected adoption and continued use | The intervention is part of the National Scale Up Plan for Infant and Young Child Feeding (2013- 2025). |
| Other factors important to adoption | Regulation and legislation such as the Code, policies, the standards, affect the breastfeeding adoption at scale supported by A&T initiative. That's why conducted active advocacy for breastfeeding regulation and policy adoption in collaboration with allied partners.* |
| ***IMPLEMENTATION*** | |
| Description of implementation strategy including underlying theory, if any, and how it may be integrated with any other interventions | By June, 2017, Alive & Thrive had trained a total of 1226 community health volunteers and 381 government health workers, in 93 local health centres in Boucle du Mouhoun. Additionally, Alive & Thrive implemented a system of supportive supervision to improve quality of interpersonal communication and equipped all local health centers in the intervention group with communication tools, including counselling cards, posters, mini-posters, and leaflets with short messages on breastfeeding for mothers to take home.  Home visits are part of the routine activities of community health volunteers in Burkina Faso, to support health promotion in the community.  Community health volunteers were instructed to prioritize mothers who had been identified by government health workers as needing additional support. |
| Cost of intervention (time or money) from the implementer perspective | - |
| Consistency of implementation across staff/time/settings/subgroups (not about differential outcomes, but process) | - |
| Adaptations made to intervention during program/project roll out (i.e., was the intervention delivered as intended?) | Adaptations were made to increase human resources, increase supervision, and modify training schedules, and monitoring tools were implemented to address these concerns. |
| Other factors important to implementation, including policy and regulatory environment. | Advocacy for breastfeeding regulation and policy adoption by A&T Burkina and allied partners.* |
| ***MAINTENANCE*** | |
| To what extent were different intervention components continued or institutionalized? How was the original program modified? | A&T Burkina paved the way for institutionalization of breastfeeding interventions through different activities. The intervention has continued over the last years.* |
| Availability/accessibility of intervention over time, and importance of these factors to adoption and sustained use | - |
| Is the intervention still ongoing at ≥ 12 months? | Ongoing |
| Description of any long-term subsidies/incentives and plans for continuity or phase-out, and their effects on adoption/sustained use | Alive & Thrive ´s work in Burkina Faso began as a partnership with the Ministry of Health (MoH) to implement the National Scale Up Plan for Infant and Young Child Feeding (2013- 2025). |
| Reference:  1. Cresswell JA, Ganaba R, Sarrassat S, Somé H, Diallo AH, Cousens S, et al. The effect of the Alive & Thrive initiative on exclusive breastfeeding in rural Burkina Faso: a repeated cross-sectional cluster randomised controlled trial. Lancet Glob Heal. 2019;7(3):e357–65.  *KI 02B | |

**Supplementary table 4.** Key elements linked to the initiative Stronger with Breast-milk Only in Burkina Faso from a perspective of the RE-AIM

| ***RE-AIM Element*** | ***Stronger with Breast milk Only*** |
| --- | --- |
| ***REACH*** | |
| Description of target population (geographic coverage, numbers targeted, demographic characteristics) | National |
| Setting characteristics (urban vs. rural, etc.) | National level (urban and rural setting) |
| What percent of potentially eligible participants a) were excluded, b) took part and c) how representative were they? | It is a national intervention |
| Characteristics of participants reached compared to non-participants or to target population | Does not apply |
| Other factors that affect reach of program including policy context, program budget constraints, conflict, and cost. | - |
| **EFFECTIVENESS** | |
| What were the goals of the program? | To promote giving babies breast milk only, on demand, and stopping the practice of giving water (and other liquids and foods), from the moment of their birth through their first six months of life. |
| What were the successes achieved toward stated goals of the program? | The increase in breastfeeding rates, 59 to 64% from 2019 to 2020, is related to the initiative.  However, the initiative is still being evaluated.* |
| What impact did the intervention have on all participants who began the program? | - |
| What was the effect on process intermediate, and primary outcomes? | - |
| Possible negative or unintended consequences of the intervention on quality of life and related factors | - |
| Economic impact | Due to the fact that the intervention is recent (2020) the economic impact has not yet been evaluated.* |
| ***ADOPTION*** | |
| What level was the program/intervention targeting (e.g., individual, community, policy)? How representative were the participating settings? | Community level |
| Description of regulations and legislation and how these affected adoption and continued use | The strategy is part of a joint effort between the Government of Burkina Faso with support from UNICEF, the World Bank, the PRSS (Health Services Reinforcement Project) of the Government of Burkina Faso, and Alive & Thrive. |
| Other factors important to adoption | The resources and funding provided by different NGO´s.(2) |
| ***IMPLEMENTATION*** | |
| Description of implementation strategy including underlying theory, if any, and how it may be integrated with any other interventions | The strategy launches in the country, in several steps and with a range of stakeholders. It is a social and behavioral change communication strategy. A toolkit was developed to guide the design and implementation of the initiative at national level. Tools and materials are available in different formats. The strategy includes a series of counselling cards that are used at the health facility and community level (Health care workers, community volunteers, community leaders, mobilizers and facilitators working in household, community or health care settings) to create dialogue and specifically address the issues of giving water and addressing issues of not enough breastmilk.  An advocacy brief has been developed to improve access to skilled breastfeeding counselling from health workers. (2) |
| Cost of intervention (time or money) from the implementer perspective | - |
| Consistency of implementation across staff/time/settings/subgroups (not about differential outcomes, but process) | - |
| Adaptations made to intervention during program/project roll out (i.e., was the intervention delivered as intended?) | Tools and materials are available in different formats, including "inDesign" files to facilitate adaptation to each context. (2) |
| Other factors important to implementation, including policy and regulatory environment. | The government has a key role in mobilizing financial resources for the implementation of the initiative. It is then up to each country to identify the potential donors present and to mobilize partners to align programs already funded in the same direction. (2) |
| ***MAINTENANCE*** | |
| To what extent were different intervention components continued or institutionalized? How was the original program modified? | Implementation has continued over the last 2 years. |
| Availability/accessibility of intervention over time, and importance of these factors to adoption and sustained use | - |
| Is the intervention still ongoing at ≥ 12 months? | Ongoing |
| Description of any long-term subsidies/incentives and plans for continuity or phase-out, and their effects on adoption/sustained use | The intervention is an alliance with the Alive&thrive and UNICEF to support the government and is funded by the three parts and have a big support from World Bank. |
| References:  1. SBWO. Stronger With Breastmilk Only. 2021;(May). Available from: https://www.breastmilkonly.com/  2. SWBO. “Stronger With Breastmilk Only” Initiative in Practice: What Does It Take? 2020.  *KI 01B | |

**Supplementary table 5.** Key elements linked Groups for training and monitoring of Infant and Young Child Feeding practices (GASPA, french) in Burkina Faso from a perspective of the RE-AIM

| **RE-AIM Element** | **Groups for training and monitoring of IYCF practices (GASPA, french)** |
| --- | --- |
| ***REACH*** | |
| Description of target population (geographic coverage, numbers targeted, demographic characteristics) | It is a national strategy |
| Setting characteristics (urban vs. rural, etc.) | National (urban and rural setting) |
| What percent of potentially eligible participants a) were excluded, b) took part and c) how representative were they? | - |
| Characteristics of participants reached compared to non-participants or to target population | Pregnant and lactating women |
| Other factors that affect the reach of the program, including policy context, program budget constraints, conflict, and cost. | Funding insufficient to reach the whole target population |
| ***EFFECTIVENESS*** | |
| What were the goals of the program? | To contribute to address both immediate and root  causes of malnutrition.(2) |
| What were the successes achieved toward stated goals of the program? | - |
| What impact did the intervention have on all participants who began the program? | In October 2020, a total of 2,699 new mother-to-mother support groups were set up. The 27,463 new pregnant and lactating women (PLW) participated in IYCF counselling sessions. This results on brings up to 376,708 the cumulative number of PLWs registered since January 2020.(1) |
| What was the effect on process intermediate, and primary outcomes? | Meeting once a month, these groups have led their members learn better nutrition practices and to a fall in the numbers of malnourished children.(3) |
| Possible negative or unintended consequences of the intervention on quality of life and related factors | - |
| Economic impact | - |
| ***ADOPTION*** | |
| What level was the program/intervention targeting (e.g., individual, community, policy)? How representative were the participating settings? | Community level |
| Description of regulations and legislation and how these affected adoption and continued use | The strategy is part of the National infant and young child feeding scale up plan.* |
| Other factors important to adoption | The coordination and support of different NGO´s* |
| ***IMPLEMENTATION*** | |
| Description of implementation strategy including underlying theory, if any, and how it may be integrated with any other interventions | The strategy uses interpersonal communication strategies to help change behavior: women are brought together in small groups according to their physiological status (pregnant women, nursing mothers, and mothers of children aged under 2 years). There, they learn good nutrition and health and hygiene practices and are offered cooking demonstrations. (3) |
| Cost of intervention (time or money) from the implementer perspective | - |
| Consistency of implementation across staff/time/settings/subgroups (not about differential outcomes, but process) | - |
| Adaptations made to intervention during program/project roll out (i.e., was the intervention delivered as intended?) | - |
| Other factors important to implementation, including policy and regulatory environment. | - |
| ***MAINTENANCE*** | |
| To what extent were different intervention components continued or institutionalized? How was the original program modified? | Implementation has continued over the last 5 years. |
| Availability/accessibility of intervention over time, and importance of these factors to adoption and sustained use | - |
| Is the intervention still ongoing at ≥ 12 months? | Ongoing |
| Description of any long-term subsidies/incentives and plans for continuity or phase-out, and their effects on adoption/sustained use | The strategy is supported by NGO´s like UNICEF, United Nations, World bank and others, in alliance with the government. |
| Reference:  1. UNICEF. Humanitarian Situation Report No.9 Burkina Faso [Internet]. 2020. Available from: http://www.coraf.org/burkina-faso/?lang=fr  2. World Food Programme. WFP Burkina Faso Country Brief [Internet]. 2019. Available from: http://www.wfp.org/countries/burkina-faso  3.African Development Food Bank. Burkina Faso: African Development Bank committed to fighting malnutrition and poverty in the Sahel [Internet]. 2019 [cited 2021 Aug 12]. Available from: https://www.afdb.org/en/news-and-events/burkina-faso-african-development-bank-committed-to-fighting-malnutrition-and-poverty-in-the-sahel-19012 | |

1. ***Mexico***

**Supplementary table 6. Key elements linked to Integrated Strategy for Attention to Nutrition (ESiAN) (package of interventions to infants and mothers) in Mexico from a perspective of the RE-AIM.**

| ***RE-AIM Element*** | ***Integrated Strategy for Attention to Nutrition (ESiAN)*** |
| --- | --- |
| ***REACH*** | |
| Description of target population (geographic coverage, numbers targeted, demographic characteristics) | Pregnant and postpartum women and children less than 5 years and healthcare providers. |
| Setting characteristics (urban vs. rural, etc.) | Urban and rural setting |
| What percent of potentially eligible participants a) were excluded, b) took part and c) how representative were they? | - |
| Characteristics of participants reached compared to non-participants or to target population | The participants (mother and children) reached are beneficiaries from the program PROSPERA (Total number of beneficiaries: 6.4 millions families (26.6 millions people) |
| Other factors that affect the reach of the program, including policy context, program budget constraints, conflict, and cost. | - |
| ***EFFECTIVENESS*** | |
| What were the goals of the program? | The EsIAN aimed to address the double burden of malnutrition and strengthen the quality of Primary Health Care, with the ultimate goal of reducing all forms of malnutrition. Actions focused on the first 1000 days of life, which correspond to the window of opportunity for child survival and optimal growth, and on children aged 2–5 years, due to the weight increase and persistent anemia in this age group. |
| What were the successes achieved toward stated goals of the program? | -*Training healthcare providers:* knowledge about breastfeeding improved significantly after training (between 18 and 24%). The score related to the knowledge of the benefits of breastfeeding improved post-training (58%) more for doctors, 53% more for nurses and 47% more for technical nurses.  *-Lactating mothers:* The proportion of women who predominantly breastfed was significantly higher at the end than baseline (64.7% compared to 94.2%) (P <0.05). |
| What impact did the intervention have on all participants who began the program? | - |
| What was the effect on process intermediate, and primary outcomes? | - |
| Possible negative or unintended consequences of the intervention on quality of life and related factors | - |
| Economic impact |  |
| ***ADOPTION*** | |
| What level was the program/intervention targeting (e.g., individual, community, policy)? How representative were the participating settings? | At community level. But, the target really are the mothers and children, the community. |
| Description of regulations and legislation and how these affected adoption and continued use | Although it was not working well, it does affect the fact that the program has been discontinued.* |
| Other factors important to adoption | - |
| ***IMPLEMENTATION*** | |
| Description of implementation strategy including underlying theory, if any, and how it may be integrated with any other interventions | The strategy included 3 components. The first one aimed to procure and maintain functioning equipment at each primary health care unit, to facilitate height and weight measurements and anemia diagnosis, as well as audiovisual equipment (TV and video) for communication purposes with mothers/caregivers. The second component comprised continued distribution of nutritional supplements free of cost to program beneficiaries with a modified scheme. For children in urban areas, the fortified complementary food (Nutrisano) was replaced with micronutrient powders, and distribution was extended to cover all beneficiary children aged 6 months to 5 years. In rural areas, children similarly received MNP, in addition to Nutrisano (6–11 months), followed by a fortified milk (12–23 months). The latter 2 were added considering that food insecurity may still contribute to child undernutrition in rural areas. The third component was a behavior change communication and training strategy, delivered through primary health care providers. |
| Cost of intervention (time or money) from the implementer perspective | - |
| Consistency of implementation across staff/time/settings/subgroups (not about differential outcomes, but process) | - |
| Adaptations made to intervention during program/project roll out (i.e., was the intervention delivered as intended?) | No important changes took place during implementation |
| Other factors important to implementation, including policy and regulatory environment. | - |
| ***MAINTENANCE*** | |
| To what extent were different intervention components continued or institutionalized? How was the original program modified? | The strategy did not continue, because of political reasons. New government began on 2018, and it was decided to discontinue the Oportunidades programs, thus ESiAN as well . |
| Availability/accessibility of intervention over time, and importance of these factors to adoption and sustained use | There is limited data about the impact of the strategy. |
| Is the intervention still ongoing at ≥ 12 months? | No, the strategy ended with the last government. * |
| Description of any long-term subsidies/incentives and plans for continuity or phase-out, and their effects on adoption/sustained use | None |
| Reference:  -Bonvecchio Arenas A, González W, Théodore FL, Lozada-Tequeanes AL, García-Guerra A, Alvarado R, et al. Translating Evidence-Based Program Recommendations into Action: The Design, Testing, and Scaling Up of the Behavior Change Strategy EsIAN in Mexico. The Journal of Nutrition. 2019;149(Suppl. 1):2310S-2322S.  *KI, 04M | |

**Supplementary table 7. Key elements linked to the National Breastfeeding Strategy in Mexico from a perspective of the RE-AIM.**

| **RE-AIM Element** | **National Breastfeeding Strategy** |
| --- | --- |
| ***REACH*** | |
| Description of target population (geographic coverage, numbers targeted, demographic characteristics) | It´s a national strategy |
| Setting characteristics (urban vs. rural, etc.) | National (urban and rural setting) |
| What percent of potentially eligible participants a) were excluded, b) took part and c) how representative were they? | No applicable |
| Characteristics of participants reached compared to non-participants or to target population | Mothers and children at national level. |
| Other factors that affect the reach of the program, including policy context, program budget constraints, conflict, and cost. | The implementation of the strategy faced resistance to change on the part of hospital authorities and health facility staff. |
| ***EFFECTIVENESS*** | |
| What were the goals of the program? | To increase the number of children who are breastfed from birth to 2-year-old. |
| What were the successes achieved toward stated goals of the program? | - |
| What impact did the intervention have on all participants who began the program? | - |
| What was the effect on process intermediate, and primary outcomes? | 1.As part of the global network of human milk banks, a day was instituted as "World Human Milk Donation Day" to encourage and promote the importance of human milk donation.  2.Public and private institutions in the federative entities installed equipped, dignified and hygienic spaces for women to breastfeed or express their milk.  3.Training through 34 courses for the training of external evaluators of the Baby-Friendly Hospital Initiative, 196 hospitals were nominated and 31 were re-nominated. |
| Possible negative or unintended consequences of the intervention on quality of life and related factors | - |
| Economic impact | - |
| ***ADOPTION*** | |
| What level was the program/intervention targeting (e.g., individual, community, policy)? How representative were the participating settings? | Community level |
| Description of regulations and legislation and how these affected adoption and continued use | From the reform to the General Health Law promoted by the Ministry of Health in 2014,  where the mandatory nature of actions that contribute to increasing the duration and adherence to breastfeeding, the National Breastfeeding Strategy emerged.  The practice of breastfeeding is considered in the Sectorial Health program 2013-2018, in the Specific Action Program for Maternal and Perinatal Health, from which the Strategy is derived. |
| Other factors important to adoption | The National Strategy did not have the formality, for example, of a specific action program or a sectoral program. |
| ***IMPLEMENTATION*** | |
| Description of implementation strategy including underlying theory, if any, and how it may be integrated with any other interventions | The strategy consist of 6 axes:  • Position the issue on the agenda of decision makers.  • Strengthen skills for the promotion, protection and support for breastfeeding  maternal in institutions.  • Promote the participation of the Private Initiative in promoting breastfeeding  maternal.  • Monitor compliance with the International Code of Marketing of  Breast Milk Substitutes.  • Promote the practice of natural breastfeeding in the municipalities of the National Crusade Against Hunger.  • Cross-cutting axis: Training for health personnel, university students in medical areas and related, as well as the general population in promoting breastfeeding. |
| Cost of intervention (time or money) from the implementer perspective | - |
| Consistency of implementation across staff/time/settings/subgroups (not about differential outcomes, but process) | - |
| Adaptations made to intervention during program/project roll out (i.e., was the intervention delivered as intended?) | No important changes took place during implementation. |
| Other factors important to implementation, including policy and regulatory environment. | The strategy did not have a solid programmatic anchor and did not have a specific budget. * |
| ***MAINTENANCE*** | |
| To what extent were different intervention components continued or institutionalized? How was the original program modified? | The strategy did not continue.  It was not a transexenal instrument. * |
| Availability/accessibility of intervention over time, and importance of these factors to adoption and sustained use | There is limited data about the impact of the strategy. * |
| Is the intervention still ongoing at ≥ 12 months? | No, the strategy ended with the last government. * |
| Description of any long-term subsidies/incentives and plans for continuity or phase-out, and their effects on adoption/sustained use | None |
| Reference:  1. González De Cosío Martínez T, Hernández Cordero S. Lactancia Materna En México. Primera ed. México; 2016.  2.Secretaría de Salud. Estrategia Nacional de Lactancia Materna 2014-2018.; 2014. <http://cnegsr.salud.gob.mx/contenidos/descargas/SMP/ENLM_2014-2018.pdf>.  3. Secretaría de Salud. Avances, Logros y Retos de los Programas de Prevención y Promoción de la Salud 2013-2018. Ciudad de México. 2020.  *KI, 05M | |

**Supplementary table 8. Key elements linked to the Baby-friendly hospital in Mexico from a perspective of the RE-AIM.**

| ***RE-AIM Element*** | ***Baby-friendly Hospital Initiave*** |
| --- | --- |
| ***REACH*** | |
| Description of target population (geographic coverage, numbers targeted, demographic characteristics) | Hospital population |
| Setting characteristics (urban vs. rural, etc.) | Urban or rural, where there is a hospital with baby-friendly nomination |
| What percent of potentially eligible participants a) were excluded, b) took part and c) how representative were they? | - |
| Characteristics of participants reached compared to non-participants or to target population | Postpartum mothers and children |
| Other factors that affect the reach of the program, including policy context, program budget constraints, conflict, and cost. | Lack of allocated and labeled resources. |
| ***EFFECTIVENESS*** | |
| What were the goals of the program? | To allow parents the free choice of modality to feed their children through information on breastfeeding, the formation of breastfeeding support networks, early contact and the restriction of supplies of breast milk substitutes in maternity or nursing obstetric care rooms. |
| What were the successes achieved toward stated goals of the program? | - |
| What impact did the intervention have on all participants who began the program? | - |
| What was the effect on process intermediate, and primary outcomes? | By 2014 the Initiative Hospital Friend of the Boy and the Girl (IHAN) was only found in 5% of the 1,097 hospitals that provide obstetric care in clinics of the Ministry of Health, the IMSS (Instituto Mexicano del Seguro Social), ISSSTE (Institute of Social Security and Services for Workers of the State) and IMSS Oportunidades (now the Social Inclusion Program, Prospera) |
| Possible negative or unintended consequences of the intervention on quality of life and related factors |  |
| Economic impact | - |
| ***ADOPTION*** | |
| What level was the program/intervention targeting (e.g., individual, community, policy)? How representative were the participating settings? | Community level |
| Description of regulations and legislation and how these affected adoption and continued use | The Baby-friendly Hospital Initiative (BFHI) is a program of the World Health Organization (WHO) and UNICEF.  The inclusion of the country in international meetings of UNICEF and WHO, such as the one in which the Innocenti Declaration (1990 and 2005) was carried out, were decisive. This made possible the exchange of experiences, review and elaboration of joint innovative action strategies, such as the Baby-friendly Hospital Initiative.  From 2014-2018 the National Breastfeeding Strategy had BFHI as one of its goals. |
| Other factors important to adoption | Lack of training of health professionals and lack of human resources. |
| ***IMPLEMENTATION*** | |
| Description of implementation strategy including underlying theory, if any, and how it may be integrated with any other interventions | The Nomination "Baby-friendly hospital initiative" is the result of evaluation processes, which determine that public and private institutions that provide health services aimed at maternal and child care, meet the "Ten steps for successful breastfeeding”.  BFHI coordination awards the WHO/UNICEF Global BFHI Nomination. |
| Cost of intervention (time or money) from the implementer perspective | - |
| Consistency of implementation across staff/time/settings/subgroups (not about differential outcomes, but process) | WHO and UNICEF stipulate a series of steps that must be followed to be nominated as a baby-friendly hospital. |
| Adaptations made to intervention during program/project roll out (i.e., was the intervention delivered as intended?) | The intervention is implemented as stipulated, following the 10 steps. |
| Other factors important to implementation, including policy and regulatory environment. | - |
| ***MAINTENANCE*** | |
| To what extent were different intervention components continued or institutionalized? How was the original program modified? | The strategy has continued the last years, but there are few resources, and in addition, very few hospitals are nominated. |
| Availability/accessibility of intervention over time, and importance of these factors to adoption and sustained use | - |
| Is the intervention still ongoing at ≥ 12 months? | Ongoing |
| Description of any long-term subsidies/incentives and plans for continuity or phase-out, and their effects on adoption/sustained use | - |
| Reference:  1. González De Cosío Martínez T, Hernández Cordero S. Lactancia Materna En México. Primera ed. México; 2016.  2. Organización Panamericana de la Salud, Organización Mundial de la Salud. La Iniciativa Hospital Amigo Del Niño En América Latina y El Caribe: Estado Actual, Retos y Oportunidades.; 2016. | |

**Supplementary table 9. Key elements linked to the Breastfeeding friendly initiative in Mexico from a perspective of the RE-AIM.**

1. ***Philippines***

**Supplementary table 9. Key elements linked to Early Essential Newborn Care (EENC) policy (package of interventions included in the Action Plan for Healthy Newborn Infants in the Western Pacific Region) in the Philippines from a perspective of the RE-AIM.**

| ***RE-AIM Element*** | ***Early Essential Newborn Care (EENC)*** |
| --- | --- |
| ***REACH*** | |
| Description of target population (geographic coverage, numbers targeted, demographic characteristics) | Pregnant and postpartum women and newborns |
| Setting characteristics (urban vs. rural, etc.) | Urban and rural setting |
| What percent of potentially eligible participants a) were excluded, b) took part and c) how representative were they? | Covers the whole population where the EENC program is in (Cambodia, China, Lao People’s Democratic Republic, Mongolia, Papua New Guinea, Philippines, Solomon Islands and Viet Nam).  EENC coaching has been conducted for 336 of 1007 doctors in the Philippines (33%), and 1388 of 3006 nurses and midwives (46%). Among the 28 hospitals, 21 (75%) had EENC teams supported by senior management. |
| Characteristics of participants reached compared to non-participants or to target population | No Aplicable |
| Other factors that affect the reach of the program, including policy context, program budget constraints, conflict, and cost. | At the local level, the lack of staff. The numbers are inadequate sometimes and they all are performing everything. * |
| ***EFFECTIVENESS*** | |
| What were the goals of the program? | To promote early and exclusive breastfeeding through improved birth practices; prolonged skin-to-skin contact of at least 90 min between mothers and newborns; and timely breastfeeding when newborns exhibit feeding cues (typically 15–90 min after birth) and elimination of both unnecessary early separation from the mother and promotion of infant formula. |
| What were the successes achieved toward stated goals of the program? | Strong dose–response effect between duration of skin-to-skin care (SSC) and the first breastfeed compared with no SSC at birth.  The EBF rate for all newborns combined was 83.5%, with rates ranging from 67.8% for babies not placed in SSC to as high as 92.7% for babies receiving 60–90 min of uninterrupted SSC. |
| What impact did the intervention have on all participants who began the program? | The package of practices improved exclusive breastfeeding rates at discharge, also mitigates the negative effect of caesarean section on EBF. |
| What was the effect on process intermediate, and primary outcomes? | Associations between exclusive breastfeeding and birth practices (vaginal delivery, companion of choice, non-supine position and eating and drinking during first and second stages of labour) and duration of SSC after birth (30–59 min, 60–89 min and longer than 90 min). |
| Possible negative or unintended consequences of the intervention on quality of life and related factors | Inappropriate maternal care practices persisted at tertiary level hospitals.  Four significant gaps between intrapartum practice and recommended evidence-based guidelines: a lack of foetal heart rate monitoring, improper use of oxytocin during labour, excessive use of episiotomy for primiparae, and application of fundal pressure.(3) |
| Economic impact | Implementation of the EINC protocol may vary depending on what cost items get included in the list of hospitalization expenses for laboratory/ancillary services, medical supplies, and medicines.  Data from three hospitals show that there is cost reduction resulting from EINC implementation for normal spontaneous deliveries (NSD). The average cost savings of implementation realized for every NSD birth is PhP2,032 (39.9 USD) and PhP742 (14.5 USD), for each of the two tertiary hospitals. Cost reduction in the two tertiary hospitals could be seen in terms of savings per delivery. For one tertiary hospital, savings per NSD is PhP757 (14.8 USD) and PhP2,338 (45.9 USD) for cesarean section deliveries (CSD). (2) |
| ***ADOPTION*** | |
| What level was the program/intervention targeting (e.g., individual, community, policy)? How representative were the participating settings? | Policy at community level. The targets are health facilities and health care providers. |
| Description of regulations and legislation and how these affected adoption and continued use | The Department of Health (DOH) issued Administrative Order 2009-0025, “Adopting New Policies and Protocol on Essential Newborn Care” in its bid to ensure more rapid reduction of infant mortality, particularly neonatal deaths. The Administrative Order provides policies and principles for health care providers regarding systematic implementation of a package newborn care interventions both time bound and no-time bound that address health risks and prevent neonatal deaths. (2) |
| Other factors important to adoption | Relatively high rate of EBF is likely to be due to EENC implementation in sampled hospitals that began incorporating EBF into routine practice. |
| ***IMPLEMENTATION*** | |
| Description of implementation strategy including underlying theory, if any, and how it may be integrated with any other interventions | The approach included introduction of a standard package of intrapartum and newborn care interventions, practice-based, clinical coaching and use of hospital quality improvement (QI) teams, routine self-assessments and action planning to reinforce optimal clinical practices, policies and work environments. |
| Cost of intervention (time or money) from the implementer perspective | The average cost savings of implementation realized for every NSD birth is PhP2,032 (39.9 USD) and PhP742 (14.5 USD), for each of the two tertiary hospitals. Cost reduction in the two tertiary hospitals could be seen in terms of savings per delivery. For one tertiary hospital, savings per NSD is PhP757 (14.8 USD) and PhP2,338 (45.9 USD) for cesarean section deliveries (CSD). (2) |
| Consistency of implementation across staff/time/settings/subgroups (not about differential outcomes, but process) | Routine antenatal practices need to be consistently applied. |
| Adaptations made to intervention during program/project roll out (i.e., was the intervention delivered as intended?) | No important changes took place during implementation. |
| Other factors important to implementation, including policy and regulatory environment. | Quality of hospital services across time that may change with case loads, case type, availability of staff or staff skills. COVID-19 resulted in a lot of backsliding of non-separation practices.* |
| ***MAINTENANCE*** | |
| To what extent were different intervention components continued or institutionalized? How was the original program modified? | Scale-up is being supported and coordinated by the DOH through a dedicated EENC focal person in the department and a Technical Working Group for Maternal and Neonatal Health. |
| Availability/accessibility of intervention over time, and importance of these factors to adoption and sustained use | Introduction of EENC policy, practice and environmental interventions during childbirth and in the early newborn period should be a key priority to help improve breastfeeding outcomes.  To address the high newborn mortality, the Essential Intrapartum and Newborn Care (EINC) Protocol was developed and adopted in the Philippines in 2010. Implementation of EINC was first initiated in 11 hospitals, following which it has been gradually rolled out to other facilities in the country. |
| Is the intervention still ongoing at ≥ 12 months? | EENC has been widely introduced in the Philippines since the adoption of the EINC as standard care in 2009 and is still ongoing. |
| Description of any long-term subsidies/incentives and plans for continuity or phase-out, and their effects on adoption/sustained use | EENC has been rolled out and largely financed by Ministries of Health in the country.  In addition, external EENC facility assessments were done biennially with direct support and supervision from ministries of health. |
| Reference  1.Li Z, Mannava P, Murray JCS, Sobel HL, Jatobatu A, Calibo A, et al. Association between early essential newborn care and breastfeeding outcomes in eight countries in Asia and the Pacific: A cross-sectional observational -study. BMJ Glob Heal. 2020;5(8):1–11.  2. Papey RD, Dincog EJ, Llorin R, Yap MS. Economic Evaluation of Essential Intrapartum and Newborn Care (EINC) Protocol in a Facility Setting in the Philippines. Philippines; 2014.  3. Masuda C, Ferolin SK, Masuda K, Smith C, Matsui M. Evidence-based intrapartum practice and its associated factors at a tertiary teaching hospital in the Philippines, a descriptive mixed-methods study. BMC Pregnancy Childbirth. 2020;20(1):1–10.  *KI 05P | |

**Supplementary table 10. Key elements linked to Official database of reported violations of Breast-milk substitute in Philippines from a perspective of the RE-AIM.**

| ***RE-AIM Element*** | ***Official database of reported violations of Breast-milk substitute*** |
| --- | --- |
| ***REACH*** | |
| Description of target population (geographic coverage, numbers targeted, demographic characteristics) | The Code is a law, however there is no policy about the platform. |
| Setting characteristics (urban vs. rural, etc.) | National, applies to the entire country. |
| What percent of potentially eligible participants a) were excluded, b) took part and c) how representative were they? | Not Applicable |
| Characteristics of participants reached compared to non-participants or to target population | No Applicable |
| Other factors that affect the reach of the program, including policy context, program budget constraints, conflict, and cost. | The village, the furthest village that has no internet connection, so they cannot report.* |
| ***EFFECTIVENESS*** | |
| What were the goals of the program? | To monitor violations in the marketing of breast milk substitutes |
| What were the successes achieved toward stated goals of the program? | Families are using the portal, they report violations of breast milk substitutes. * |
| What impact did the intervention have on all participants who began the program? | Mothers and families are identifying and reporting violations using the portal. |
| What was the effect on process intermediate, and primary outcomes? | Labelling of formula products is in general compliance with national measures. |
| Possible negative or unintended consequences of the intervention | . |
| Economic impact |  |
| ***ADOPTION*** | |
| What level was the program/intervention targeting (e.g., individual, community, policy)? How representative were the participating settings? | National-level |
| Description of regulations and legislation and how these affected adoption and continued use | In 1986 the government enacted Executive Order 51 (EO51), known as the Philippine Milk Code, and in 2006 the government and with the support of other agencies, revised its implementing rules and regulations to clarify mechanism for EO51 implementation. Through EO51, Inter-Agency Committe was established which serves as a body that regulates advertising and marketing of BMS. The committee is composed of representatives from the Department of Health, Department of Trade and Industry, Department of Justice and the Department of Social Welfare and Development. The Department of Justice is involved as they are the ones to prosecute those who will be found violative of the EO51. * |
| Other factors important to adoption | The milk industries were and are constantly lobbying. * |
| ***IMPLEMENTATION*** | |
| Description of implementation strategy including underlying theory, if any, and how it may be integrated with any other interventions | The reports in the database were obtained through a crowd-sourced reporting system that utilizes mobile and web-based platforms with standardized form fields. The data included information about violators, product types, type of violations, and channels. |
| Cost of intervention (time or money) from the implementer perspective | - |
| Consistency of implementation across staff/time/settings/subgroups (not about differential outcomes, but process) | No Applicable |
| Adaptations made to intervention during program/project roll out (i.e., was the intervention delivered as intended?) | No Applicable |
| Other factors important to implementation, including policy and regulatory environment. | The action against the violators is still weak. Sanctions need to be implemented. * |
| ***MAINTENANCE*** | |
| To what extent were different intervention components continued or institutionalized? How was the original program modified? | Implementation has continued over the last years. |
| Availability/accessibility of intervention over time, and importance of these factors to adoption and sustained use | The Philippines, with its affordable web based and crowd-sourced methods, sets an example for sustainable national monitoring. Government should engage with NGOs in related fields (e.g., public health, child nutrition, women and children welfare, and consumer rights) for more strategic and accessible monitoring. |
| Is the intervention still ongoing at ≥ 12 months? | Ongoing |
| Description of any long-term subsidies/incentives and plans for continuity or phase-out, and their effects on adoption/sustained use | Department of Health |
| Reference  Ching C, Zambrano P, Nguyen TT, Tharaney M, Zafimanjaka MG, Mathisen R. Old tricks, new opportunities: How companies violate the international code of marketing of breast-milk substitutes and undermine maternal and child health during the covid-19 pandemic. Int J Environ Res Public Health. 2021;18(5):1–29.  *KI 01P, 04P, 05P | |

1. ***United States of America***

**Supplementary table 11.** **Key elements linked to the adoption of the Special Supplemental Nutrition Program for Women, Infants and Children (WIC) in USA from a perspective of the RE-AIM**

| ***RE-AIM Element*** | ***Special Supplemental Nutrition Program for Women, Infants, and Children (WIC)*** |
| --- | --- |
| ***REACH*** | |
| Description of target population (geographic coverage, numbers targeted, demographic characteristics) | The program is available in all 50 States, 34 Indian Tribal Organizations, American Samoa, District of Columbia, Guam, Commonwealth of the Northern Mariana Islands, Puerto Rico, and the Virgin Islands. It covered low-income pregnant, breastfeeding, and non-breastfeeding postpartum women, and infants and children up to age five who are found to be at nutritional risk. |
| Setting characteristics (urban vs. rural, etc.) | Urban and rural setting. The services are provided at a variety of clinic locations including, but not limited to, county health departments, hospitals, schools, and Indian Health Service facilities. |
| What percent of potentially eligible participants a) were excluded, b) took part and c) how representative were they? | In the average month of 2018, an estimated 11.9 million people were eligible to receive benefits from the WIC program. Of that group, 6.7 million people participated in the program, meaning the share of eligible people who participated in WIC (the coverage rate) was 57 percent. (7) |
| Characteristics of participants reached compared to non-participants or to target population | The requirements for pregnant, postpartum and breastfeeding women, infants and children up to age 5 to be considered eligible are: income eligibility, State residency, and being at “nutrition risk” (determined by a health professional or a trained health official). |
| Other factors that affect the reach of the program, including policy context, program budget constraints, conflict, and cost. | Many states distribute waivers that extend program rules, change work requirements, and extend program timelines that affect eligibility and participation in WIC. |
| ***EFFECTIVENESS*** | |
| What were the goals of the program? | To safeguard the health of low-income women, infants, and children up to age 5 who are at nutritional risk, by providing nutritious foods to supplement diets, nutrition education (including breastfeeding promotion and support), and referrals to health and other social services. |
| What were the successes achieved toward stated goals of the program? | Breastfeeding initiation rates in WIC have increased from 42% in 1998 to 71% in 2016. **  The WIC food package helped children score higher on the healthy eating index.  The healthcare needs of children participating in both WIC are better met than low income children not participating in WIC. (5) |
| What impact did the intervention have on all participants who began the program? | Mothers´ participation in the Peer Counseling Program is positively associated with breastfeeding initiation and breastfeeding continuation through 12 months of age. |
| What was the effect on process intermediate, and primary outcomes? | -Women who participate in WIC give birth to healthier babies who are more likely to survive infancy.  -WIC supports more nutritious diets and better infant feeding practices. WIC participants buy and eat more fruits, vegetables, whole grains, and low-fat dairy products, following the introduction of improved WIC food packages more closely aligned to current dietary guidance.  -Low-income children participating in WIC are just as likely to be immunized as more affluent children, and are more likely to receive preventive medical care than other low-income children.  -Mothers participating in the Peer Counseling Program are positively associated with breastfeeding initiation and breastfeeding continuation through 12 months of age. |
| Possible negative or unintended consequences of the intervention on quality of life and related factors | - |
| Economic impact | WIC helps to lower healthcare costs and improve health outcomes for its participants. Preterm births cost the US over $26 billion a year, with average first-year medical costs for a premature/low birth-weight baby of $49,033 compared to $4,551 for a baby born without complications.  An increase of one pound at birth very low birth-weight babies, saves approximately $28,000 in  first year’s medical costs.  The annual cost of WIC participation per participant is approximately $856,47.(5) |
| ***ADOPTION*** | |
| What level was the program/intervention targeting (e.g., individual, community, policy)? How representative were the participating settings? | It is a national program targeted to low-income Pregnant and postpartum women that live in the US. |
| Description of regulations and legislation and how these affected adoption and continued use | The WIC program was launched as a two-year pilot program in 1972. In 1975 it was established as a permanent program (P.L. 94-105).  In the mid-1980s, the Food and Nutrition Service (FNS) of the United States Department of Agriculture (USDA) launched a three-year project to study the range of breastfeeding promotion and support efforts nationwide. In 1997, the Secretary of Agriculture officially launched the Loving Support campaign during the celebration of World Breastfeeding Week; and in 2004, FNS launched the  Loving Support Peer Counseling Program, an initiative that brought the importance of breastmilk for human babies to the forefront in WIC.  In 1992, WIC food package VII was created to encourage breastfeeding for fully breastfeeding women. (6) |
| Other factors important to adoption |  |
| ***IMPLEMENTATION*** | |
| Description of implementation strategy including underlying theory, if any, and how it may be integrated with any other interventions | WIC is implemented by state agencies among the country.  WIC provides four core services: nutrition education, breastfeeding support, healthy food packages, and referrals to health and social services. WIC helps ensure that kids enter kindergarten healthy and ready to learn. |
| Cost of intervention (time or money) from the implementer perspective | WIC is federally funded through the annual appropriations process; states are not required to contribute funds. Since 1997, Congress (on a bipartisan basis) has provided sufficient funding each year for WIC to serve all eligible applicants. The program receives approximately $6 billion annually.(4) |
| Consistency of implementation across staff/time/settings/subgroups (not about differential outcomes, but process) | Most Breastfeeding Peer Counseling programs provide both prenatal and postnatal support, while others offer postnatal support. Some programs include mailing. |
| Adaptations made to intervention during program/project roll out (i.e., was the intervention delivered as intended?) | Peer counselors are women from the community with a specific training to provide breastfeeding education and support in a culturally appropriate manner and when possible in a woman’s preferred language. |
| Other factors important to implementation, including policy and regulatory environment. | The WIC program is bipartisan, has the support of both big political parties in the US. * |
| ***MAINTENANCE*** | |
| To what extent were different intervention components continued or institutionalized? How was the original program modified? | The implementation of the four components of the WIC program has continued since its establishment. |
| Availability/accessibility of intervention over time, and importance of these factors to adoption and sustained use | - |
| Is the intervention still ongoing at ≥ 12 months? | Ongoing |
| Description of any long-term subsidies/incentives and plans for continuity or phase-out, and their effects on adoption/sustained use | The amount of money provided depends on an annual appropriation from Congress. (5)  The Agriculture appropriations bill designates funding for the Special Supplemental Nutrition Program for Women, Infants, and Children (WIC) program, including the breastfeeding peer counselor program.(8) |
| References:  1. McCoy MB, Geppert J, Dech L, Richardson M. Associations Between Peer Counseling and Breastfeeding Initiation and Duration: An Analysis of Minnesota Participants in the Special Supplemental Nutrition Program for Women, Infants, and Children (WIC). Matern Child Health J. 2018;22(1):71–81.  2. Reeder JA, Joyce T, Sibley K, Arnold D, Altindag O. Telephone peer counseling of breastfeeding among WIC participants: A randomized controlled trial. Pediatrics. 2014;134(3):e700–9.  3. Martinez-Brockman JL, Harari N, Segura-Pérez S, Goeschel L, Bozzi V, Pérez-Escamilla R. Impact of the Lactation Advice Through Texting Can Help (LATCH) Trial on Time to First Contact and Exclusive Breastfeeding among WIC Participants. J Nutr Educ Behav [Internet]. 2018;50(1):33-42.e1. Available from: <https://doi.org/10.1016/j.jneb.2017.09.001>  4. Center on Budget and Policy Priorities. Policy Basics: Special Supplemental Nutrition Program for Women, Infants, and Children. Special Supplemental Nutrition Program for Women, Infants, and Children. https://www.cbpp.org/research/food-assistance/special-supplemental-nutrition-program-for-women-infants-and-children. Published 2017. Accessed July 30, 2021.  5. National WIC Association. For a Stronger Healthier America. 2019;(March). Available from: <https://fns-prod.azureedge.net/sites/default//les/ops/WICNSACostStudy.pdf>.  6. National WIC Association. WIC’s promotion and support of breastfeeding. 2019;(June).  7. National WIC Association. WIC Eligibility and Coverage Rates - 2018 [Internet]. 2018 [cited 2021 Aug 6]. Available from: https://www.fns.usda.gov/wic/eligibility-and-coverage-rates-2018#1  8. USBC. USBC: Federal Appropriations for Breastfeeding [Internet]. U.S. Breastfeeding Committee. 2021 [cited 2021 Aug 9]. Available from: http://www.usbreastfeeding.org/page/federal-appropriations-for-breastfeeding  9. Carlson S, Neuberger Z. WIC Works: Addressing the Nutrition and Health Needs of Low-Income Families for More Than Four Decades What Works to Reduce Poverty. CBPP Policy Futur. 2021;1–33.  *KI 04U | |

***WIC targets a wide range of benefits and potential indicators of impact, including a strong peer counseling component to support and promote breastfeeding.**

**Supplementary Table 12. Key elements linked to the adoption of the Paid Family Leave on Breastfeeding in the US from a perspective of the RE-AIM.**

| ***RE-AIM Element*** | ***Paid Family Leave*** |
| --- | --- |
| ***REACH*** | |
| Description of target population (geographic coverage, numbers targeted, demographic characteristics) | Parents after the birth of a child living in certain states of the US (California, New Jersey, Rhode Island and New York) |
| Setting characteristics (urban vs. rural, etc.) | urban and rural setting |
| What percent of potentially eligible participants a) were excluded, b) took part and c) how representative were they? | The policy covers the working population of some states in the US. |
| Characteristics of participants reached compared to non-participants or to target population | Not applicable |
| Other factors that affect the reach of the program, including policy context, program budget constraints, conflict, and cost. | Mothers who did not take maternity leave tended to be younger, unmarried/not cohabitating, and have a poverty-income ratio < 185%.  Younger women and those with lower paying jobs tend to have less job security, they may be less likely to take maternity leave for fear of dismissal.(4) |
| ***EFFECTIVENESS*** | |
| What were the goals of the program? | To improve infant health outcomes, including reduced infant hospitalizations, low birthweight, premature birth, and infant mortality, as well as improved breastfeeding duration. |
| What were the successes achieved toward stated goals of the program? | Married women improved in exclusive breastfeeding at 3 months, any breastfeeding at 6 months, and exclusive breastfeeding duration.  Middle- and high-income women improved in ever breastfeeding, exclusive breastfeeding at 3 months, and breastfeeding duration. |
| What impact did the intervention have on all participants who began the program? | Paid family leave allowed mothers to modestly extend breastfeeding during infancy. |
| What was the effect on process intermediate, and primary outcomes? | The paid family leave policies increased the percentage of children exclusively breastfed at 6 months.  The paid family leave enactment allowed mothers to modestly extend breastfeeding during infancy, this practice is thought to influence infants’ risk of infection and other outcomes later in childhood. |
| Possible negative or unintended consequences of the intervention on quality of life and related factors | - |
| Economic impact | - |
| ***ADOPTION*** | |
| What level was the program/intervention targeting (e.g., individual, community, policy)? How representative were the participating settings? | US state-level paid family leave policies on  breastfeeding. |
| Description of regulations and legislation and how these affected adoption and continued use | California became the first state in the nation to pass a Paid Family Leave law when it was enacted on September 23, 2002.  The paid family leave policy was passed in California in 2002 but did not begin paying benefits to eligible parents until July 1, 2004. |
| Other factors important to adoption |  |
| ***IMPLEMENTATION*** | |
| Description of implementation strategy including underlying theory, if any, and how it may be integrated with any other interventions | The details vary by state, but paid family leave programs provide 6 to 12 weeks of partially paid leave per year to workers for bonding with a new child or caring for a sick child, spouse, parent, or domestic partner.  Rhode Island and New York require that leave-takers be able to return to their jobs. In California and New Jersey, the laws don’t provide job protection, so whether workers can return is up to their employer.  The policy offers coverage to mothers after pregnancy-related temporary disability insurance ends and to new mothers who do not have pregnancy-related disability insurance. It also provides coverage to new fathers and new parents of foster or adopted children. |
| Cost of intervention (time or money) from the implementer perspective | - |
| Consistency of implementation across staff/time/settings/subgroups (not about differential outcomes, but process) | - |
| Adaptations made to intervention during program/project roll out (i.e., was the intervention delivered as intended?) | - |
| Other factors important to implementation, including policy and regulatory environment. | Because the California and New Jersey policies provide only partially paid leave, the benefits may not be sufficient to support low-income workers who can ill afford any loss of wages, thereby exacerbating health disparities. |
| ***MAINTENANCE*** | |
| To what extent were different intervention components continued or institutionalized? How was the original program modified? | Implementation has continue over the last years |
| Availability/accessibility of intervention over time, and importance of these factors to adoption and sustained use | - |
| Is the intervention still ongoing at ≥ 12 months? | Ongoing |
| Description of any long-term subsidies/incentives and plans for continuity or phase-out, and their effects on adoption/sustained use | These states administer and fund paid leave through employer and/or employee payroll contributions.(6) |
| Reference:   1. Hamad R, Modrek S, White JS. Paid family leave effects on breastfeeding: A quasi-experimental study of US policies. Am J Public Health. 2019;109(1):164–6. 2. Huang R, Yang M. Paid maternity leave and breastfeeding practice before and after California’s implementation of the nation’s first paid family leave program. Econ Hum Biol. 2015;16:45-59. doi:10.1016/j.ehb.2013.12.009 3. Montoya-Williams D, Passarella M, Lorch SA. The impact of paid family leave in the United States on birth outcomes and mortality in the first year of life. Health Serv Res. 2020;55(S2):807-814. doi:10.1111/1475-6773.13288 4. Mirkovic KR, Perrine CG, Scanlon KS. Paid Maternity Leave and Breastfeeding Outcomes. Birth. 2016;43(3):233–9. 5. US-DOL. What’s the Difference? Paid Sick Leave, FMLA, and Paid Family and Medical Leave. United States Dep Labor [Internet]. 2016;13706(September 2016):1–3. Available from: https://www.dol.gov/sites/default/files/PaidLeaveFinalRuleComparison.pdf 6. KFF. Paid Family and Sick Leave in the U.S. [Internet]. Women´s Health Policy. 2020 [cited 2021 Aug 10]. Available from: https://www.kff.org/womens-health-policy/fact-sheet/paid-family-leave-and-sick-days-in-the-u-s/ | |

**Supplementary table 13. Key elements linked to the Baby-friendly hospital initiative in the US from a perspective of the RE-AIM.**

| ***RE-AIM Element*** | ***Baby-friendly hospital initiative*** |
| --- | --- |
| ***REACH*** | |
| Description of target population (geographic coverage, numbers targeted, demographic characteristics) | Women giving births at a health facility |
| Setting characteristics (urban vs. rural, etc.) | Urban and rural setting |
| What percent of potentially eligible participants a) were excluded, b) took part and c) how representative were they? | The program covers the whole population where the Baby friendly hospitals are in. |
| Characteristics of participants reached compared to non-participants or to target population | - |
| Other factors that affect the reach of the program, including policy context, program budget constraints, conflict, and cost. | One common objection to adopting the BFHI principles is the perceived costs associated with adhering to Step 5, the step requiring that the hospital pay fair market price for formula used during the hospital stay. (2) |
| ***EFFECTIVENESS*** | |
| What were the goals of the program? | To improve breastfeeding outcomes, including increased rates of breastfeeding initiation, breastfeeding exclusivity, and longer breastfeeding duration. |
| What were the successes achieved toward stated goals of the program? | Exclusive breastfeeding rates were significantly higher for hospitals with the Baby-friendly (BF) designation (62%) as compared with those without BF designation (48%). |
| What impact did the intervention have on all participants who began the program? | Relationship between BF hospital practices that mothers experience and their achievement of exclusive breastfeeding goals. |
| What was the effect on process intermediate, and primary outcomes? | Mean difference in exclusive breastfeeding rates between hospitals with the BF designation versus without the designation. |
| Possible negative or unintended consequences of the intervention on quality of life and related factors | Disparities in breastfeeding rates related to race/ethnicity, education, income, and poverty status. |
| Economic impact | Added costs for a new Baby-Friendly Hospital will approximate $148 per birth, but these costs sharply decrease over time as breastfeeding rates increase in a Baby-Friendly environment.(3) |
| ***ADOPTION*** | |
| What level was the program/intervention targeting (e.g., individual, community, policy)? How representative were the participating settings? | US state-level |
| Description of regulations and legislation and how these affected adoption and continued use | - |
| Other factors important to adoption | The political support matched by large public investment and targeted training and technical assistance may have accelerated  adoption and reach of the Ten Steps. (4) |
| ***IMPLEMENTATION*** | |
| Description of implementation strategy including underlying theory, if any, and how it may be integrated with any other interventions | The 4-D Pathway is a tool for facilities to use to measure their progress along the journey that leads to BF designation. To achieve the BF designation, facilities first register with Baby-Friendly USA. They then complete all of the requirements in each of the four phases of the process (Discovery, development, dissemination, designation). Ultimately, the facility demonstrates that they have attained the gold standard of maternity practices that support optimal infant nutrition during an on-site assessment. At the on-site assessment, the facility must demonstrate to Baby-Friendly USA assessors that they have correctly integrated all of the Ten Steps to Successful Breastfeeding and the International Code of Marketing of Breast-milk Substitutes into their maternity practices.  To maintain the designation the hospital required: annual quality improvement and redesignation. |
| Cost of intervention (time or money) from the implementer perspective | It is a long process. It takes about four years and then on-site assessment and designation. * |
| Consistency of implementation across staff/time/settings/subgroups (not about differential outcomes, but process) | - |
| Adaptations made to intervention during program/project roll out (i.e., was the intervention delivered as intended?) | No important changes took place during implementation. |
| Other factors important to implementation, including policy and regulatory environment. | Baby-Friendly designation necessitates multiple transition stages in order to become fully implemented, requiring a hospital to invest or redirect limited resources, such as  personnel time, information technology, and financial capital. (3) |
| ***MAINTENANCE*** | |
| To what extent were different intervention components continued or institutionalized? How was the original program modified? | Implementation has continued over the last years |
| Availability/accessibility of intervention over time, and importance of these factors to adoption and sustained use | - |
| Is the intervention still ongoing at ≥ 12 months? | Ongoing |
| Description of any long-term subsidies/incentives and plans for continuity or phase-out, and their effects on adoption/sustained use | - |
| References:  1. Patterson JA, Keuler NS, Olson BH. The effect of Baby-friendly status on exclusive breastfeeding in U.S. hospitals. Matern Child Nutr. 2018;14(3):1–6.  2. Vergie H. The Baby-Friendly Hospital Initiative in US Hospitals. Infant, Child, Adolesc Nutr [Internet]. 2015;7(4). Available from: http://www.sagepub.com/journalsPermissions.nav.  3. Dellifraine J, Langabeer J, Delgado R, Williams JF, Gong A. A transition strategy for becoming a Baby-Friendly Hospital: Exploring the costs, benefits, and challenges. Breastfeed Med. 2013;8(2):170–5.  *KI 01U | |

**Supplementary table 14. Key elements linked to the Report cards on Breastfeeding in the US from a perspective of the RE-AIM.**

| ***RE-AIM Element*** | ***Report cards*** |
| --- | --- |
| ***REACH*** | |
| Description of target population (geographic coverage, numbers targeted, demographic characteristics) | National (hospitals) |
| Setting characteristics (urban vs. rural, etc.) | National |
| What percent of potentially eligible participants a) were excluded, b) took part and c) how representative were they? | Not Applicable |
| Characteristics of participants reached compared to non-participants or to target population | Not applicable |
| Other factors that affect the reach of the program, including policy context, program budget constraints, conflict, and cost. | - |
| ***EFFECTIVENESS*** | |
| What were the goals of the program? | To provide data on breastfeeding practices and support in all states, the District of Columbia (DC), Puerto Rico, Guam, and the US Virgin Islands. |
| What were the successes achieved toward stated goals of the program? | The states and local coalitions expect the data every year, to use it. To know what they are doing to support breastfeeding.  High response rates of 70 percent. The report card gets a lot of information from the hospitals on the practices and provides individualized reports back to every single hospital. * |
| What impact did the intervention have on all participants who began the program? | CDC’s national survey of Maternity Practices in Infant Nutrition and Care (mPINC) assesses maternity care practices and provides feedback to encourage hospitals to make improvements that better support breastfeeding. |
| What was the effect on process intermediate, and primary outcomes? | - |
| Possible negative or unintended consequences of the intervention on quality of life and related factors | - |
| Economic impact |  |
| ***ADOPTION*** | |
| What level was the program/intervention targeting (e.g., individual, community, policy)? How representative were the participating settings? | Community level. |
| Description of regulations and legislation and how these affected adoption and continued use | - |
| Other factors important to adoption | - |
| ***IMPLEMENTATION*** | |
| Description of implementation strategy including underlying theory, if any, and how it may be integrated with any other interventions | CDC releases annual breastfeeding rates.  In addition, every 2 years, all maternity care hospitals in the United States and US territories are invited to participate. Hospitals are asking about early postpartum care practices, feeding practices, education and support of mothers and caregivers, staff and provider responsibilities and training, and hospital policies and procedures. These policies and practices are organized into six main areas of care called subdomains that are scored and comprise each state’s total maternity Practices in infant nutrition and care (mPINC) score. Data can be used to monitor and improve evidence-based maternity care practices and policies. |
| Cost of intervention (time or money) from the implementer perspective | - |
| Consistency of implementation across staff/time/settings/subgroups (not about differential outcomes, but process) | - |
| Adaptations made to intervention during program/project roll out (i.e., was the intervention delivered as intended?) | No important change has been made during the implementation. |
| Other factors important to implementation, including policy and regulatory environment. | Consistent funding from CDC. * |
| ***MAINTENANCE*** | |
| To what extent were different intervention components continued or institutionalized? How was the original program modified? | Implementation has continued over the last 13 years. |
| Availability/accessibility of intervention over time, and importance of these factors to adoption and sustained use | - |
| Is the intervention still ongoing at ≥ 12 months? | Ongoing |
| Description of any long-term subsidies/incentives and plans for continuity or phase-out, and their effects on adoption/sustained use | The CDC has a government budget. |
| References:  1. Center for Disease Control and Prevention. Breastfeeding Report Card United States, 2020. Breastfeed Rep Card United States, 2020 [Internet]. 2020;(37):6. Available from: <https://www.cdc.gov/breastfeeding/pdf/2020-Breastfeeding-Report-Card-H.pdf>  *KI 05U | |
